# Long-term spatio-temporal trends in burden of fungal skin diseases in middle-aged and elderly people from 1990 to 2021

**DOI:** 10.1371/journal.pntd.0014157

**Published:** 2026-04-01

**Authors:** Qinglian Qin, Yuyuan Huang, Zedan Yang, Xiaoting Wei, Tongxue Qin, Jinming Su, Jie Liu, Rongfeng Chen, Wudi Wei, Zongxiang Yuan, Jingzhen Lai, Li Ye, Hao Liang, Junjun Jiang

**Affiliations:** 1 Guangxi Key Laboratory of AIDS Prevention and Treatment, School of Public Health, Guangxi Medical University, Nanning, Guangxi, China; 2 Joint Laboratory for Emerging Infectious Diseases in China (Guangxi)-ASEAN, Life Sciences Institute, Guangxi Medical University, Nanning, Guangxi, China; Universidad de Antioquía: Universidad de Antioquia, COLOMBIA

## Abstract

**Background:**

As a high incidence group, the elderly face the burden of fungal skin diseases, which has remained poorly quantified. This study aims to analyse the spatiotemporal trends in the burden of fungal skin diseases in middle-aged and elderly people from 1990 to 2021.

**Methods:**

Data were obtained from the Global Burden of Disease (GBD) Study 2021. This study analysed incident cases, prevalent cases, disability-adjusted life years (DALYs), and their corresponding rates for fungal skin diseases in middle-aged and elderly people stratified by sex, age, socio-demographic index (SDI), GBD regions, and countries. Average annual percent change (AAPC) was calculated to assess temporal trends in the burden of fungal skin diseases.

**Results:**

From 1990 to 2021, the global incidence, prevalence, and DALYs rates of fungal skin diseases in middle-aged and elderly people were consistently higher than those in the entire population. Globally, incident cases, prevalent cases, and DALYs of fungal skin diseases in middle-aged and elderly people increased by 124.09%, 124.13%, and 123.26%, respectively. In 2021, the incidence, prevalence, and DALYs rates were 38255.44 (95% uncertainty interval [UI]: 32832.61–44492.76), 12186.46 (95% UI: 10721.21–14030.06), and 64.66 (95% UI: 26.34–133.26) per 100,000 population, respectively. From 1990 to 2021, the incidence, prevalence, and DALYs rates exhibited overall upward trends, with AAPCs of 4.12% (95% confidence interval [CI]: 3.04%–5.20%), 4.18% (95% CI: 3.11%–5.24%), and 2.89% (95% CI: 1.89%–3.89%), respectively. Geographically, the highest burden was concentrated in Andean Latin America, Australasia, and Western Europe.

**Conclusions:**

The global burden of fungal skin diseases in middle-aged and elderly people has increased over the past three decades, with significant disparities across sexes, SDI levels, regions, and countries. Targeted public health interventions and resource allocation are required to reduce the burden of fungal skin diseases in this vulnerable population.

## Author summary

Fungal skin diseases pose a significant public health threat, yet the disease burden in the elderly population has remained poorly quantified. Using data from the GBD 2021 study, we analysed temporal trends in the incidence, prevalence, and DALYs in middle-aged and elderly people from 1990 to 2021. Our findings reveal that the incidence, prevalence, and DALYs rates of fungal skin diseases in this population were consistently higher than those in the entire population. Geographically, both low and high SDI regions exhibited the highest disease burden, with the most notable concentrations in Andean Latin America, Australasia, and Western Europe. Notably, the global incidence rate, prevalence rate, and DALYs rate demonstrated a significant upward trend, with AAPC of 4.12%, 4.18%, and 2.89%, respectively. These results underscore the urgent need for targeted interventions, strategic resource allocation, and age-specific healthcare strategies to mitigate the growing impact of fungal skin diseases in this aging population.

## Introduction

Fungal skin diseases are the most common infectious skin diseases, causing both superficial and deep infections [1]. Although most fungal skin infections are typically mild, they can significantly impair the quality of life for affected individuals, potentially interfering with social activities. Chronic, refractory, or recurrent fungal infections not only prolong the duration of treatment and increase healthcare costs but also have the potential to induce psychological complications such as anxiety and depression [2]. Notably, in immunocompromised individuals, including patients with HIV/AIDS, organ transplantation, cancer, or diabetes mellitus, fungal infections may become severe and resistant to treatment [3–5]. In recent years, the global incidence of fungal skin infections has increased due to multiple interrelated factors. Climate change and rapid urbanization have led to increased ambient temperature and humidity, creating more favorable environments for fungal growth and transmission [6,7]. The widespread use of immunosuppressants, particularly during the COVID-19 pandemic, has increased the susceptibility of the population [8]. Furthermore, increased global travel and migration have facilitated the cross-regional spread of fungal pathogens [9]. According to the Global Burden of Disease (GBD) 2021 study, approximately 1.73 billion people worldwide suffered from fungal skin diseases in 2021, marking a 67.93% increase since 1990 [10].

Fungal skin diseases can affect individuals across all age groups. Research has shown that elderly individuals are at increased risk of fungal skin infections due to age-related physiological decline, including immunosenescence, as well as a higher prevalence of chronic comorbidities and potentially poor lifestyle habits [11,12]. Studies based on the GBD 2021 data have demonstrated that the burden of fungal skin diseases remains relatively stable before 55 years but increased rapidly thereafter [10,13,14]. The aging population may contribute to increased incidence of fungal infections. According to the World Health Organization (WHO), the global population of older adults is projected to reach 1.4 billion by 2030 and 2.1 billion by 2050 [15]. With increasing life expectancy and rapidly ageing population, the growing burden of fungal skin diseases in the elderly will pose a serious challenge.

A comprehensive understanding of the epidemiological trends of fungal skin diseases is essential for the effective allocation of healthcare resources and the development of age-specific management strategies. Previous studies have reported the global burden of fungal skin diseases in the entire population [10,13,16], but the unique vulnerabilities and epidemiological patterns in middle-aged and elderly people have received limited attention. In low-and middle-income countries (LMICs), the true incidence of fungal skin diseases is likely substantially underestimated due to weak surveillance systems and limited public health resources [13,17,18]. Therefore, this study aims to systematically analyse the temporal trends in the incidence, prevalence, and disability-adjusted life years (DALYs) of fungal skin diseases in middle-aged and elderly people from 1990 to 2021. We also aim to assess variations in disease burden across age groups, sexes, socio-demographic index (SDI) levels, GBD regions, and countries.

## Materials and methods

### Data source

Data on the burden of fungal skin diseases from 1990 to 2021 were obtained from the Global Burden of Disease (GBD) 2021 study, conducted by the Institute for Health Metrics and Evaluation (IHME), using the Global Health Data Exchange (GHDx) results tool (https://gbd2021.healthdata.org/gbd-results/). The GBD 2021 study provides comparable and systematic estimates of 371 diseases, impairments, and injuries across 204 countries and territories from 1990 to 2021 [19]. Data sources include vital registration systems, household surveys, censuses, hospital records, and disease-specific registries. To address missing data and inconsistencies in country-level reporting, the GBD study employed multiple modeling techniques, such as DisMod-MR 2.1, the Cause of Death Ensemble Model (CODEm), and spatiotemporal Gaussian process regression (STGPR) [20]. All estimates are presented with 95% uncertainty intervals (UI), accounting for statistical uncertainty and variability in data quality. Further details on methodological approaches of the GBD 2021 study have been published elsewhere [21,22].

In this study, we focused on adults aged 55 years and older. As justified in the Introduction section, a threshold of 55 years was selected due to documented increases in cutaneous vulnerability beginning at this age. The population was divided into seven age groups: 55–59, 60–64, 65–69, 70–74, 75–79, 80–84, and 85 + years. We extracted annual incident cases, prevalent cases, disability-adjusted life years (DALYs), incidence rate, prevalence rate, DALYs rate, and corresponding 95% UI for fungal skin diseases from 1990 to 2021. DALYs are calculated by summing the years of life lost (YLLs) and years lived with disability (YLDs). Since the GBD study does not model deaths caused by fungal skin diseases, the DALYs for these conditions are comprised entirely of YLDs [13].

SDI, as a composite indicator, was defined as the weighted geometric mean of three components including total fertility rate, income per capita, and mean education in people aged 15 years and older [23]. SDI ranges from 0 to 1, with higher values reflecting greater socioeconomic status. The 204 countries and territories were categorized into five groups based on country-level SDI estimates: low, low-middle, middle, high-middle, and high SDI regions (S1 Table). In addition, based on geographical contiguity and socioeconomic similarity, the 204 countries and territories were further grouped into 21 GBD regions, including Southeast Asia, High-income Asia Pacific, Western Europe, and others (S2 Table).

### Statistical analyses

We described the incident cases, prevalent cases, DALYs, and their corresponding rates for fungal skin diseases in middle-aged and elderly people, stratified by sex, age, SDI, GBD regions, and 204 countries and territories. The average annual percent change (AAPC) was calculated using Joinpoint regression analysis to evaluate temporal trends in fungal skin diseases from 1990 to 2021. To prevent overfitting, each model was limited to a maximum of five joinpoints (six line segments). Model selection was based on the Weighted Bayesian Information Criterion (Weighted BIC) and the recommendations of the Joinpoint Regression Program. To evaluate the robustness of our findings, we varied the maximum number of joinpoints, which did not change the optimal model. The application of alternative selection criteria, including the permutation test and the Modified BIC, also produced consistent results. An upward trend was observed if the lower bound of the estimated AAPC and its 95% confidence intervals (CI) were greater than zero. Conversely, a downward trend was identified if the upper bound of both the projected AAPC and its 95% CI were less than zero. If neither condition was met, the trend was considered stable during the observed period.

Joinpoint regression analysis was performed using the Joinpoint Regression Program (Version 5.0.1, National Cancer Institute, Rockville, MD, USA). All other statistical analyses and mapping were conducted using R software (Version 4.4.1, the R foundation for statistical computing, Vienna, Austria). A two-tailed *P* value less than 0.05 was considered statistically significant.

## Results

### Global burden and trend of fungal skin diseases in middle-aged and elderly people

From 1990 to 2021, the incident cases, prevalent cases, and DALYs for fungal skin diseases in middle-aged and elderly people worldwide increased by 124.09%, 124.13%, and 123.26%, respectively (Tables 1, S3 and S4). Specifically, the incident cases rose from 253679277 (95% UI: 216416895–298135974) in 1990 to 568470554 (95% UI: 487888004–661156203) in 2021 (Table 1). The prevalent cases increased from 80796031(95% UI: 70744457–93330520) in 1990 to 181089158 (95% UI: 159315707–208484706) in 2021 (S3 Table). DALYs also increased from 430374(95% UI: 174296–886604) in 1990 to 960854 (95% UI: 391476–1980242) in 2021(S4 Table).

**Table 1:** Incident cases and incidence rate of fungal skin diseases in middle-aged and elderly people in 1990 and 2021, and temporal trends from 1990 to 2021.

Characteristic	1990		2021		1990-2021	
	Incident cases,	Incidence rate per 100000	Incident cases,	Incidence rate per 100000	Cases change	AAPC
	No. (95% UI)	No. (95% UI)	No. (95% UI)	No. (95% UI)	% (95% UI)	% (95% CI)
Global	253679277(216416895,298135974)	37782.19(32232.45,44403.43)	568470554(487888004,661156203)	38255.44(32832.61,44492.76)	124.09(121.47,127.32)	4.12(3.04,5.20)*
Sex						
Male	112830785(95648114,133708798)	36223.69(30707.29,42926.46)	259629347(221880140,306456923)	37115.47(31719.01,43809.74)	130.11(126.45,134.20)	8.03(6.87,9.18)*
Female	140848492(120034177,163817472)	39130.86(33348.18,45512.16)	308841206(266970383,357511184)	39269.38(33945.47,45457.80)	119.27(116.71,122.62)	1.03(-0.16,2.22)
Age groups						
55–59 years	44899018(36186259,54575230)	24243.54(19539.02,29468.27)	94532641(76257337,115156382)	23888.29(19270.14,29099.88)	110.55(109.14,112.01)	-4.71(-5.99,-3.43)*
60–64 years	42812596(31202604,57603281)	26656.37(19427.65,35865.48)	83953524(61257555,113027393)	26231.55(19140.12,35315.78)	96.10(94.72,97.38)	-5.22(-7.09,-3.35)*
65–69 years	42272684(33770573,52717536)	34198.58(27320.38,42648.47)	89646919(71312792,112216509)	32499.35(25852.75,40681.42)	112.07(110.35,113.67)	-16.78(-19.02,-14.54)*
70–74 years	38375668(30921017,48193197)	45328.54(36523.26,56924.81)	90184664(72564997,113044246)	43813.16(35253.24,54918.71)	135.00(133.93,136.18)	-10.64(-12.98,-8.30)*
75–79 years	35392854(25687153,47034044)	57497.45(41730.06,76409.15)	71082869(51322788,94373594)	53897.86(38914.98,71557.81)	100.84(98.99,102.78)	-20.79(-22.82,-18.76)*
80–84 years	27937144(22767708,34471354)	78972.19(64359.33,97442.97)	65662246(53382313,81056011)	74971.40(60950.50,92547.59)	135.04(132.97,137.08)	-16.50(-18.53,-14.46)*
85 + years	21989313(17929410,26477324)	107715.00(87827.50,129699.59)	73407690(59407160,88297963)	106293.26(86020.70,127854.16)	233.83(228.86,238.37)	-4.16(-5.45,-2.87)*
SDI regions						
Low SDI	17138699(14443279,20126730)	45938.38(38713.61,53947.47)	37826526(32053612,44461934)	46097.31(39062.15,54183.56)	120.71(119.13,122.35)	1.15(-0.52,2.82)
Low-middle SDI	36005824(30597231,42586009)	35719.76(30354.14,42247.67)	89069439(75833331,105168643)	36945.55(31455.28,43623.41)	147.38(144.49,150.77)	11.02(10.18,11.86)*
Middle SDI	56831996(48269251,67260590)	32744.77(27811.19,38753.39)	162576611(138588994,192028276)	34601.19(29495.90,40869.38)	186.07(181.80,190.56)	17.85(16.21,19.49)*
High-middle SDI	65108415(55018640,76563998)	37738.60(31890.29,44378.57)	126671865(108335706,147513425)	36538.84(31249.73,42550.65)	94.56(91.36,98.39)	-10.43(-12.57,-8.28)*
High SDI	78237157(67442335,90981498)	41958.46(36169.22,48793.23)	151680733(131486456,175212389)	43963.77(38110.57,50784.28)	93.87(89.74,98.75)	14.83(12.44,17.21)*
GBD regions						
Andean Latin America	2147755(1824694,2548671)	63999.61(54372.91,75946.27)	6498238(5518712,7657153)	65596.14(55708.36,77294.75)	202.56(199.54,205.68)	7.99(7.29,8.70)*
Australasia	2340195(2035390,2705407)	59403.20(51666.07,68673.68)	5496122(4806751,6364957)	62213.79(54410.41,72048.65)	134.86(131.52,138.71)	14.64(12.64,16.64)*
Caribbean	2509488(2107725,2978258)	58228.48(48906.24,69105.50)	5479500(4644745,6478711)	59183.71(50167.58,69976.12)	118.35(115.86,121.65)	5.25(4.15,6.36)*
Central Asia	3378803(2847139,3941597)	42245.34(35597.93,49281.99)	5804062(4889392,6812658)	39891.05(33604.57,46823.08)	71.78(67.82,75.28)	-19.23(-25.27,-13.18)*
Central Europe	11480357(9705200,13344496)	43288.84(36595.28,50317.92)	18239904(15497886,21164187)	49259.75(41854.50,57157.24)	58.88(54.22,64.18)	42.01(39.94,44.08)*
Central Latin America	5429453(4643570,6329124)	40010.38(34219.10,46640.18)	18830614(16140979,21967317)	44031.51(37742.35,51366.05)	246.82(234.68,257.44)	30.37(28.55,32.18)*
Central Sub-Saharan Africa	1592082(1332138,1910364)	42339.21(35426.38,50803.50)	3857393(3246149,4620751)	42748.20(35974.30,51207.85)	142.29(140.11,144.61)	3.06(1.48,4.64)*
East Asia	34188945(28706488,40861500)	22952.84(19272.18,27432.48)	94965362(80753747,113068774)	24218.39(20594.10,28835.19)	177.77(171.07,186.03)	17.32(15.52,19.11)*
Eastern Europe	21400044(18062747,25062976)	43769.29(36943.55,51261.05)	29073636(24657095,33642268)	46833.28(39718.90,54192.67)	35.86(33.60,38.51)	21.02(16.02,26.03)*
Eastern Sub-Saharan Africa	7088454(5956628,8346347)	58266.22(48962.75,68605.95)	15782786(13319709,18527607)	58373.51(49263.68,68525.38)	122.65(120.20,125.59)	0.59(-0.60,1.78)
High-income Asia Pacific	16087867(13606536,19096881)	46007.10(38911.14,54612.09)	41329425(35028756,48357548)	58620.50(49683.80,68588.99)	156.90(143.31,172.63)	79.09(75.47,82.71)*
High-income North America	12930589(11404593,14458391)	22321.80(19687.50,24959.21)	25408224(22504685,28055225)	22578.21(19998.07,24930.38)	96.50(91.73,101.89)	3.43(1.95,4.91)*
North Africa and Middle East	7408366(6265358,8738900)	26211.49(22167.42,30919.04)	20408830(17265945,23981183)	26771.36(22648.67,31457.41)	175.48(173.20,178.05)	7.06(5.50,8.61)*
Oceania	137941(115647,164565)	28672.69(24038.61,34206.78)	359198(302258,424462)	29104.54(24490.91,34392.69)	160.40(157.99,162.94)	4.97(2.36,7.59)*
South Asia	29896611(25079063,35285352)	31489.25(26415.07,37165.06)	81866781(69539886,96270700)	32971.58(28006.97,38772.72)	173.83(168.93,180.09)	14.94(13.83,16.05)*
Southeast Asia	17019653(14462912,20054663)	40196.25(34157.86,47364.20)	46273287(39315045,54487984)	40394.07(34319.90,47565.05)	171.88(171.05,172.86)	1.53(0.41,2.64)*
Southern Latin America	3636461(3089084,4251551)	45906.38(38996.34,53671.23)	7233593(6180919,8450183)	49154.29(42001.08,57421.36)	98.92(95.37,102.96)	22.08(21.18,22.97)*
Southern Sub-Saharan Africa	2512078(2122504,2962381)	56772.99(47968.61,66949.85)	5409851(4558586,6407590)	55569.50(46825.39,65818.19)	115.35(113.62,117.05)	-6.98(-9.00,-4.96)*
Tropical Latin America	8542947(7148184,10032220)	56421.33(47209.71,66257.13)	26315711(22230893,30665088)	59406.29(50185.03,69224.77)	208.04(203.21,213.77)	16.65(16.24,17.07)*
Western Europe	55143073(46439308,65097605)	56782.84(47820.25,67033.38)	90856452(76850001,106266282)	60922.50(51530.68,71255.34)	64.76(61.40,68.82)	22.51(21.08,23.94)*
Western Sub-Saharan Africa	8808115(7447298,10380901)	61017.07(51590.19,71912.34)	18981584(16083050,22365561)	59053.33(50035.75,69581.17)	115.50(112.9,118.18)	-10.56(-11.76,-9.36)*

Abbreviation: UI, uncertainty interval; AAPC, average annual percent change; CI, confidence interval; SDI, socio-demographic index.

Note: * indicates statistically significant.

**Fig 1:**
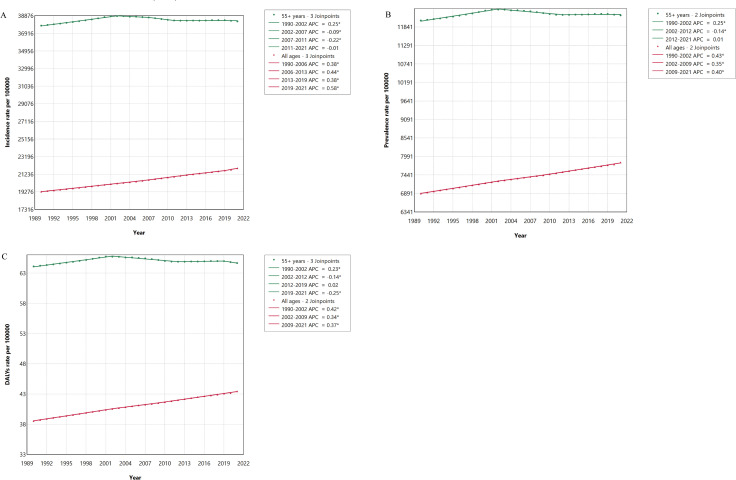
Burden of fungal skin diseases in middle-aged and elderly people and the entire population from 1990 to 2021. (A) Incidence rate; (B) Prevalence rate; (C) Disability adjusted life years rate.

From 1990 to 2021, the global incidence, prevalence, and DALYs rates in middle-aged and elderly people were considerably higher than those observed in the entire population (Fig 1). In 2021, the incidence, prevalence, and DALYs rates of fungal skin diseases in the entire population were 21912.90, 7812.76, and 43.46 per 100000 population, respectively (S5 Table). In middle-aged and elderly people, the incidence rate, prevalence rate, and DALYs rate were 38255.44 (95% UI: 32832.61–44492.76), 12186.46 (95% UI: 10721.21–14030.06), and 64.66 (95% UI: 26.34–133.26) per 100000 population, respectively (Tables 1, S3 and S4). From 1990 to 2021, the incidence, prevalence, and DALYs rates of fungal skin diseases in middle-aged and elderly people exhibited an upward trend, with AAPCs of 4.12% (95% CI: 3.04%–5.20%), 4.18% (95% CI: 3.11%–5.24%), and 2.89% (95% CI: 1.89%–3.89%), respectively (Tables 1, S3 and S4).

### Burden of fungal skin diseases in middle-aged and elderly people by sex and age groups

In 2021, the incident cases of fungal skin diseases in middle-aged and elderly people were 259629347 (95% UI: 221880140–306456923) for males and 308841206 (95% UI: 266970383–357511184) for females (Table 1). For males, the incidence, prevalence, and DALYs rates were 37115.47 (95% UI: 31719.01–43809.74), 12006.91 (95% UI: 10529.95–13937.88), and 64.17 (95% UI: 26.18–131.58) per 100000 population, respectively (Tables 1, S3 and S4). The corresponding rates for females were 39269.38 (95% UI: 33945.47–45457.80), 12346.17 (95% UI: 10928.76–14196.50), and 65.10 (95% UI: 26.45–134.79) per 100000 population, respectively (Tables 1, S3 and S4). While females showed marginally higher values across all three metrics, the overlapping 95% uncertainty intervals suggest that these differences are not statistically significant.

**Fig 2:**
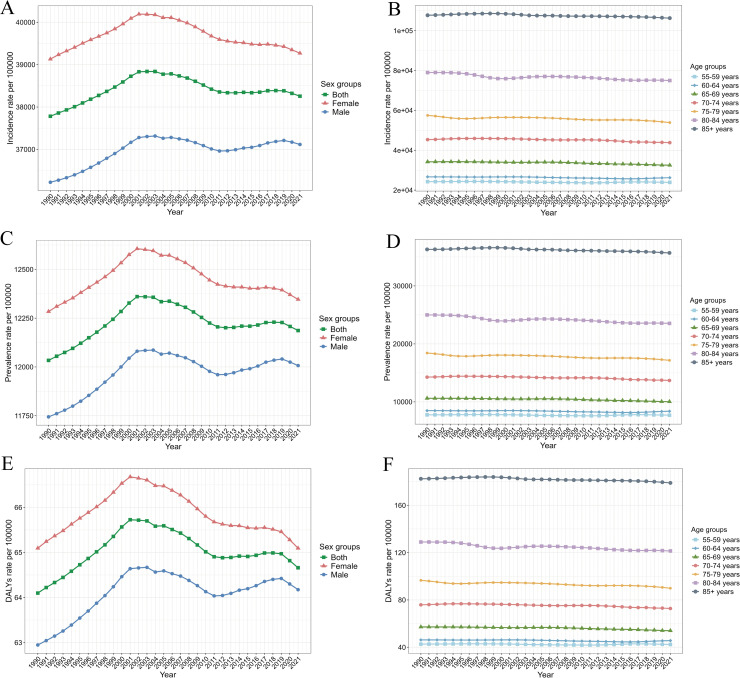
Burden of fungal skin diseases in middle-aged and elderly people grouped by sex and age groups from 1990 to 2021. (A) Incidence rate by sex groups; (B) Incidence rate by age groups; (C) Prevalence rate by sex groups; (D) Prevalence rate by age groups; (E) DALYs rate by sex groups; (F) DALYs rate by age groups. Abbreviations: DALYs, disability adjusted life years.

Across age groups, the incidence, prevalence, and DALYs rates consistently increased with age, with the highest rates observed in individuals aged 85 years and older (Fig 2). In 2021, the incidence, prevalence, and DALYs rates of fungal skin diseases in this age group were 106293.26 (95% UI: 86020.70–127854.16), 35689.94 (95% UI: 30019.04–41697.19), and 178.96 (95% UI: 70.52–372.25) per 100000 population, respectively (Tables 1, S3 and S4). From 1990 to 2021, the incident cases, prevalent cases, and DALYs increased across all age groups.

### Burden of fungal skin diseases in middle-aged and elderly people by SDI level

**Fig 3:**
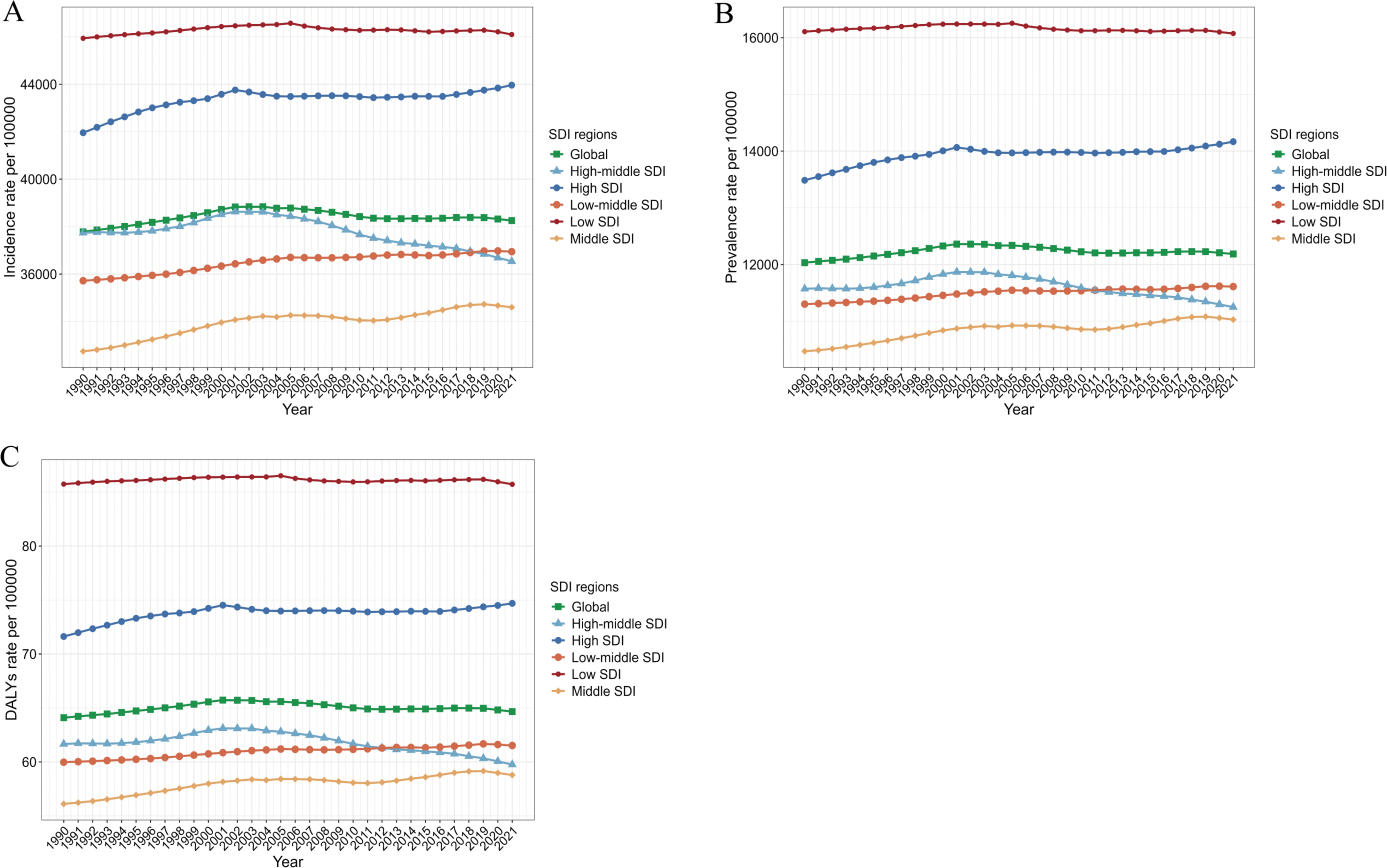
Burden of fungal skin diseases in middle-aged and elderly people by SDI regions from 1990 to 2021. (A) Incidence rate; (B) Prevalence rate; (C) DALYs rate. Abbreviations: DALYs, disability adjusted life years; SDI, Socio-demographic index.

At the SDI level, the incident cases, prevalent cases, and DALYs due to fungal skin diseases in middle-aged and elderly people increased across all SDI regions from 1990 to 2021. In 2021, the middle SDI region reported the highest incident cases, prevalent cases, and DALYs. From 1990 to 2021, the highest incidence, prevalence, and DALYs rates were observed in low SDI region, followed by high SDI region (Fig 3). In 2021, the incidence, prevalence, and DALYs rates in low SDI region were 46097.31 (95% UI: 39062.15–54183.56), 16074.65 (95% UI: 13852.01–18639.65), and 85.72 (95% UI: 34.91–177.09) per 100000 population, respectively (Tables 1, S3 and S4). From 1990 to 2021, the incidence rate of fungal skin diseases in middle-aged and elderly people showed an upward trend in all SDI regions except for high-middle SDI region, with the highest increase observed in middle SDI region (AAPC = 17.85%, 95% CI: 16.21%–19.49%) (Table 1). Similarly, the prevalence and DALYs rates exhibited an upward trend in all SDI regions except low and high-middle SDI regions (S3 and S4 Tables). At the SDI level, the 95% UI for prevalence estimates narrowed progressively (S1 Fig). In low-SDI regions, prevalence estimates were associated with wider 95% UI, reflecting greater uncertainty.

### Burden of fungal skin disease in middle-aged and elderly people at regional level

At the regional level in 2021, the incident cases of fungal skin diseases in middle-aged and elderly people were highest in East Asia (94965362, 95% UI: 80753747–113068774), followed by Western Europe (90856452, 95% UI: 76850001–106266282) and South Asia (81866781, 95% UI: 69539886–96270700) (Table 1). The highest numbers of prevalent cases and DALYs were observed in Western Europe, at 30499166 (95% UI: 26629527–35180924) and 161114 (95% UI: 65884–336220), respectively (S3 and S4 Tables). From 1990 to 2021, the incident cases, prevalent cases, and DALYs increased in all GBD regions, with the largest increases in Central Latin America, Tropical Latin America and Andean Latin America (Tables 1, S3 and S4).

**Fig 4:**
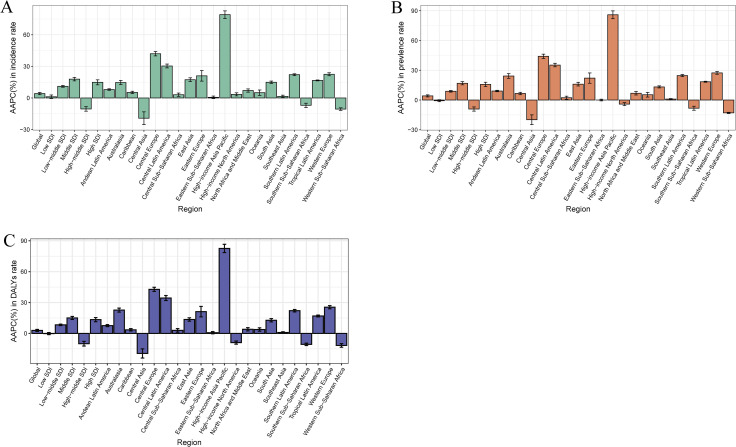
The AAPC of fungal skin diseases in middle-aged and elderly people from 1990 to 2021. (A) Incidence rate; (B) Prevalence rate; (C) DALYs rate. Abbreviations: AAPC, average annual percent change; DALYs, disability adjusted life years.

In 2021, the highest incidence rate of fungal skin disease in middle-aged and elderly people was reported in Andean Latin America (65596.14 per 100000 population, 95% UI: 55708.36–77294.75), followed by Australasia (62213.79 per 100000 population, 95% UI: 54410.41–72048.65) and Western Europe (60922.50 per 100000 population, 95% UI: 51530.68–71255.34) (Table 1). Andean Latin America also had the highest prevalence and DALYs rates, at 26373.42 (95% UI: 22752.45–30602.08) and 140.94 (95% UI: 57.39–299.34) per 100000 population, respectively. In contrast, High-income North America reported the lowest incidence, prevalence, and DALYs rates, at 22578.21 (95% UI: 19998.07–24930.38), 6729.21 (95% UI: 6366.14–7199.79), and 34.90 (95% UI: 14.60–69.53) per 100000 population, respectively. From 1990 to 2021, the incidence rate increased in all GBD regions except for Southern Sub-Saharan Africa, Western Sub-Saharan Africa and Central Asia, with the greatest increase in High-income Asia Pacific (AAPC = 79.09%, 95% CI: 75.47%–82.71%) (Table 1, Fig 4). Similarly, the prevalence and DALYs rates increased in 17 GBD regions and decreased in 4 GBD regions, with the highest upward trend in High-income Asia Pacific (Fig 4).

### Burden of fungal skin diseases in middle-aged and elderly people at national level

**Fig 5:**
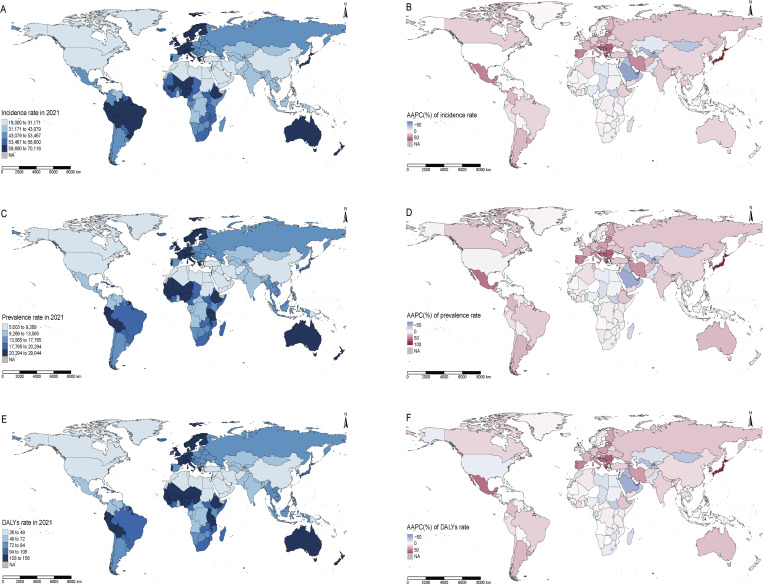
Burden of fungal skin diseases in middle-aged and elderly people across 204 countries and territories. (A) Incidence rate in 2021; (B) AAPC of incidence rate from 1990 to 2021; (C) Prevalence rate in 2021; (D) AAPC of prevalence rate from 1990 to 2021; (E) DALYs rate in 2021; (F) AAPC of DALYs rate from 1990 to 2021. Abbreviations: AAPC, average annual percent change; DALYs, disability adjusted life years. The terms of free use for these shapefiles are available here: https://www.naturalearthdata.com/about/terms-of-use/, Base map shapefile source: https://www.naturalearthdata.com/downloads/50m-cultural-vectors/50m-admin-0-countries-2/.

At the national level in 2021, the highest incident cases of fungal skin diseases in middle-aged and elderly people were reported in China (91947079, 95% UI: 78103337–109515584), India (65935474, 95% UI: 55720147–77370165), and Japan (32810601, 95% UI: 27826074–38582593) (Fig 5, S6 Table). These three countries also had the highest prevalent cases and DALYs (Fig 5, S7 and S8 Tables). From 1990 to 2021, the incident cases, prevalent cases, and DALYs increased in all countries and territories except Niue.

In 2021, the highest incidence rate of fungal skin diseases in this population was observed in Ethiopia (67415.23 per 100000 population, 95% UI: 56960.49–79718.72), followed by Israel (66649.87 per 100000 population, 95% UI: 56367.77–78607.82) and Puerto Rico (66570.78 per 100000 population, 95% UI: 56491.73–77865.81) (Fig 5, S6 Table). From 1990 to 2021, 124 countries and territories exhibited an upward trend in incidence rate, with the largest increases observed in Japan (AAPC = 95.04%, 95% CI: 89.82%–100.26%), Lebanon (AAPC = 67.07%, 95% CI: 63.33%–70.81%), and Croatia (AAPC = 60.42%, 95% CI: 56.11%–64.73%) (Fig 5, S6 Table). These three countries also experienced the greatest increase in prevalence and DALYs rates over the study period (Fig 5, S7 and S8 Tables).

## Discussion

This study comprehensively assessed the global epidemiology, long-term trends, and regional disparities in the burden of fungal skin diseases in middle-aged and elderly population. Our study found that the burden of fungal skin diseases in middle-aged and elderly people was significantly higher than in the entire population and increased globally. Substantial variations were observed across age groups, sexes, SDI levels, GBD regions, and countries. These findings highlight the urgent need for targeted public health interventions and strategic resource allocation to address the growing challenges of fungal skin diseases, particularly in aging population worldwide.

Globally, the burden of fungal skin diseases in middle-aged and elderly people exhibited an overall upward trend, paralleling the pattern observed in the entire population [10]. First, climate change particularly global warming has led to higher ambient temperatures and humidity levels, creating a more favorable environment for fungal proliferation and transmission [24]. The elderly are particularly vulnerable to fungal infections due to age-related physiological changes, including impaired skin barrier function and reduced immune defense. Second, rising antifungal resistance poses a growing public health challenge [25,26]. Resistant infections are more likely to persist or recur, often necessitating prolonged therapy and contributing to higher healthcare burden. Third, the increase in global life expectancy and the rapid aging of the population have resulted in a growing number of elderly individuals. This demographic shift means that the absolute number of elderly people suffering from fungal skin diseases has also risen, contributing to the overall increase in disease burden.

Our study revealed that the burden of fungal skin diseases in middle-aged and elderly people were consistently higher than that in the entire population. First, physiological changes in the skin of the elderly, such as decreased stratum corneum lipid levels and thinning of the epidermis and dermis, impair skin barrier function and weaken defense against external stimuli [27]. Second, with advancing age, the immune system of the elderly gradually declines, and the skin immune barrier function weakens. This compromised immune response makes it difficult to effectively resist fungal invasion, facilitating fungal colonization, proliferation, and subsequent infection [28]. Third, the elderly commonly suffer from multiple chronic comorbidities that significantly increase the risk of fungal skin infections. For example, diabetic patients often present with wounds characterized by elevated glucose levels, poor vascularization, and compromised immune function, which collectively promote fungal colonization and growth [29,30]. Malignancies, particularly hematological cancers, often involve immunosuppression either from the disease itself or from chemotherapy, reducing the host’s ability to mount effective antifungal responses [31]. The long-term use of corticosteroids and immunosuppressants for managing these chronic conditions further compromises both systemic and local immune defenses, exacerbating vulnerability to fungal infections [31]. Fourth, lifestyle and behavioral factors, including reduced mobility, poor hygiene practices, and limited access to care can further exacerbate the risk of fungal infections in elderly populations [32]. Health education campaigns should be implemented to raise awareness of fungal skin infections among older adults and their caregivers, with an emphasis on proper personal hygiene, early symptom recognition, and timely medical consultation. Additionally, in healthcare settings, routine skin assessments and fungal screening should be integrated into geriatric care protocols, especially for patients with comorbidities such as diabetes or those residing in long-term care facilities.

In our study, the incidence, prevalence, and DALYs rates of fungal skin diseases in middle-aged and elderly people were highest in both low and high SDI regions. The geographical variations may be attributed to the population structure, environmental factors, and medical diagnostic capabilities [33,34]. In low SDI region, limited access to clean water and sanitation increases the risk of skin exposure to fungal pathogens, thereby raising infection rates [35]. Additionally, environmental factors such as high humidity, poor ventilation, overcrowded living conditions, and frequent close contact among individuals create a favorable environment for fungal growth and transmission [36]. In low- and middle-income countries, recurrent, chronic, and extensive fungal skin diseases were most common [7]. In high SDI region, the higher proportion of elderly individuals, who experience declines in immune and skin barrier functions along with increased prevalence of chronic diseases, contributes to elevated infection risks [37]. Environmental and climatic factors in these regions may also contribute to fungal proliferation. Furthermore, the widespread availability of advanced diagnostic technologies in high SDI countries facilitates earlier and more accurate detection and reporting of fungal skin diseases, potentially contributing to the observed higher rates.

The burden of fungal skin diseases demonstrated geographic disparities across regions and countries. Our study found that East Asia, Western Europe, and South Asia recorded the highest absolute numbers of incident cases, prevalent cases, and DALYs. The monsoon climate in Asia results in consistently high humidity, which fosters an environment conducive to fungal growth and reproduction. Notably, China, Japan, and India ranked among the top countries globally in all three metrics. Relatively higher proportion of the elderly population in these regions provides a larger susceptible population for the incidence of fungal skin diseases [38,39]. Notably, our study revealed that the burden of fungal skin diseases in middle-aged and elderly people has increased in most countries from 1990 to 2021. These findings underscore the growing public health significance of fungal skin diseases, particularly among older adults. To address this growing burden, strengthening international collaboration on cross-border disease surveillance and climate-sensitive infectious disease modeling will be critical to effectively responding to the emerging threats posed by fungal skin diseases in the context of global demographic and environmental shifts. Furthermore, it is essential to establish national screening protocols in high-prevalence countries and to integrate fungal disease surveillance into existing public health monitoring systems.

While this study provides important insights into the global burden of fungal skin diseases, several limitations should be acknowledged. First, the GBD 2021 study primarily relies on model estimations rather than real-world data. These estimates may be influenced by inherent biases resulting from variations in data quality, reporting systems, and collection methods across different regions and countries [40]. Second, the GBD study does not distinguish between different clinical forms of fungal skin infections (e.g., tinea corporis, tinea pedis, candidiasis), nor does it provide species-level identification. The epidemiology, transmission routes, and drug resistance patterns can vary significantly between fungal species. For instance, onychomycosis shows a higher incidence in developed regions, while tinea capitis remains predominantly a disease of developing countries [41]. Third, in low- and middle-income countries, the lack of standardized diagnostic criteria and inadequate reporting mechanisms may lead to an underestimation of the true burden of fungal skin diseases [21].

## Conclusion

In conclusion, the burden of fungal skin diseases in middle-aged and elderly people was significantly higher than that in the entire population and increased globally from 1990 to 2021. Significant disparities in the burden of fungal skin diseases were observed across sex, age, and geographic regions, with older individuals experiencing a disproportionately higher burden. The incidence, prevalence, and DALYs rates of fungal skin diseases were highest in low and high SDI regions. With global warming and aging population, fungal skin diseases in middle-aged and elderly people should be paid attention to. Urgent attention is needed for targeted public health interventions and resource allocation to alleviate the growing disease burden within this vulnerable group. Our findings provide a foundation for future research efforts, including community surveys and economic-epidemiological modeling.

## Supporting information

S1 Table The SDI regions and the countries within each SDI regions in 2021. (DOCX)

S2 Table The GBD regions and the countries within each GBD regions. (DOCX)

S3 Table Prevent cases and prevalence rate of fungal skin diseases in middle-aged and elderly people in 1990 and 2021, and temporal trends from 1990 to 2021. (DOCX)

S4 Table Number of DALYs and DALYs rate of fungal skin diseases in middle-aged and elderly people in 1990 and 2021, and temporal trends from 1990 to 2021. (DOCX)

S5 Table The burden of fungal skin diseases among middle-aged and elderly people and the entire population from 1990 to 2021. (DOCX)

S6 Table Incident cases and incidence rate of fungal skin diseases in middle-aged and elderly people across 204 countries and territories in 1990 and 2021, and temporal trends from 1990 to 2021. (DOCX)

S7 Table Prevalent cases and prevalence rate of fungal skin diseases in middle-aged and elderly people across 204 countries and territories in 1990 and 2021, and temporal trends from 1990 to 2021. (DOCX)

S8 Table Number of DALYs and DALYs rate of fungal skin diseases in middle-aged and elderly people across 204 countries and territories in 1990 and 2021, and temporal trends from 1990 to 2021. (DOCX)

S1 Fig Prevalence rate of fungal skin diseases in middle-aged and elderly people at global and SDI levels from 1990 to 2021. (TIF)
