# Supplementary material for: Long-term spatio-temporal trends in burden of fungal skin diseases in middle-aged and elderly people from 1990 to 2021
Source: PLoS Negl Trop Dis. 2026 Apr 1;20(4):e0014157. doi: 10.1371/journal.pntd.0014157 (PMC13065042; doi:10.1371/journal.pntd.0014157)
Supplement: S1 Table — (DOCX) [file pntd.0014157.s001.docx]

**S1 Table. The SDI regions and the countries within each SDI regions in 2021.**

| SDI regions | Country | SDI-value in 2021 |
| --- | --- | --- |
| High SDI region |  |  |
|  | Switzerland | 0.934 |
|  | Norway | 0.917 |
|  | Monaco | 0.910 |
|  | Germany | 0.904 |
|  | Denmark | 0.897 |
|  | Netherlands | 0.888 |
|  | San Marino | 0.888 |
|  | Sweden | 0.887 |
|  | Republic of Korea | 0.887 |
|  | Luxembourg | 0.885 |
|  | Taiwan (Province of China) | 0.875 |
|  | Iceland | 0.875 |
|  | Ireland | 0.874 |
|  | Canada | 0.873 |
|  | Japan | 0.871 |
|  | Andorra | 0.870 |
|  | United States of America | 0.863 |
|  | Finland | 0.860 |
|  | United Kingdom | 0.858 |
|  | Lithuania | 0.858 |
|  | Singapore | 0.856 |
|  | Austria | 0.855 |
|  | Belgium | 0.854 |
|  | New Zealand | 0.850 |
|  | United Arab Emirates | 0.850 |
|  | Kuwait | 0.847 |
|  | Qatar | 0.847 |
|  | Estonia | 0.846 |
|  | Australia | 0.844 |
|  | Slovenia | 0.843 |
|  | France | 0.838 |
|  | Cyprus | 0.836 |
|  | Greenland | 0.836 |
|  | Latvia | 0.831 |
|  | Czechia | 0.829 |
|  | Puerto Rico | 0.825 |
|  | United States Virgin Islands | 0.823 |
|  | Bermuda | 0.821 |
|  | Saudi Arabia | 0.815 |
|  | Poland | 0.812 |
| High-middle SDl region |  |  |
|  | Brunei Darussalam | 0.810 |
|  | Russian Federation | 0.809 |
|  | Israel | 0.809 |
|  | Slovakia | 0.808 |
|  | Italy | 0.806 |
|  | Bahamas | 0.805 |
|  | Guam | 0.802 |
|  | Malta | 0.802 |
|  | Croatia | 0.799 |
|  | Montenegro | 0.797 |
|  | Serbia | 0.792 |
|  | Greece | 0.792 |
|  | Hungary | 0.791 |
|  | Belarus | 0.784 |
|  | Cook Islands | 0.778 |
|  | Northern Mariana Islands | 0.778 |
|  | Oman | 0.774 |
|  | Chile | 0.770 |
|  | Spain | 0.769 |
|  | Trinidad and Tobago | 0.769 |
|  | Romania | 0.766 |
|  | Bulgaria | 0.765 |
|  | Ukraine | 0.761 |
|  | Saint Kitts and Nevis | 0.756 |
|  | Palau | 0.755 |
|  | Bahrain | 0.752 |
|  | North Macedonia | 0.751 |
|  | Antigua and Barbuda | 0.750 |
|  | Dominica | 0.747 |
|  | Barbados | 0.747 |
|  | Portugal | 0.745 |
|  | Malaysia | 0.743 |
|  | Lebanon | 0.741 |
|  | Libya | 0.735 |
|  | Argentina | 0.734 |
|  | Georgia | 0.733 |
|  | Republic of Moldova | 0.732 |
|  | Seychelles | 0.728 |
|  | American Samoa | 0.726 |
|  | Niue | 0.726 |
|  | Jordan | 0.725 |
|  | Bosnia and Herzegovina | 0.723 |
|  | Uruguay | 0.722 |
|  | China | 0.719 |
|  | Kazakhstan | 0.718 |
|  | Mauritius | 0.718 |
|  | Turkey | 0.713 |
| Middle SDI region |  |  |
|  | Albania | 0.707 |
|  | Panama | 0.707 |
|  | Costa Rica | 0.704 |
|  | Armenia | 0.702 |
|  | Sri Lanka | 0.701 |
|  | Iran (Islamic Republic of) | 0.697 |
|  | Azerbaijan | 0.695 |
|  | Tokelau | 0.687 |
|  | Jamaica | 0.683 |
|  | Turkmenistan | 0.683 |
|  | Thailand | 0.683 |
|  | Tunisia | 0.682 |
|  | South Africa | 0.681 |
|  | Saint Lucia | 0.673 |
|  | Grenada | 0.669 |
|  | Cuba | 0.669 |
|  | Fiji | 0.669 |
|  | Ecuador | 0.666 |
|  | Mexico | 0.665 |
|  | Uzbekistan | 0.665 |
|  | Equatorial Guinea | 0.664 |
|  | Iraq | 0.663 |
|  | Peru | 0.662 |
|  | Algeria | 0.660 |
|  | Indonesia | 0.658 |
|  | Maldives | 0.658 |
|  | Colombia | 0.657 |
|  | Philippines | 0.652 |
|  | Guyana | 0.651 |
|  | Paraguay | 0.650 |
|  | Brazil | 0.649 |
|  | Botswana | 0.643 |
|  | Suriname | 0.641 |
|  | Saint Vincent and the Grenadines | 0.641 |
|  | Gabon | 0.639 |
|  | Palestine | 0.629 |
|  | Tonga | 0.629 |
|  | Nauru | 0.628 |
|  | Syrian Arab Republic | 0.623 |
|  | Viet Nam | 0.622 |
|  | Dominican Republic | 0.619 |
| Low-middle SDI region |  |  |
|  | Mongolia | 0.619 |
|  | Namibia | 0.618 |
|  | Belize | 0.611 |
|  | Kyrgyzstan | 0.609 |
|  | Bolivia (Plurinational State of) | 0.604 |
|  | Egypt | 0.604 |
|  | Venezuela (Bolivarian Republic of) | 0.597 |
|  | Samoa | 0.592 |
|  | Micronesia (Federated States of) | 0.588 |
|  | Congo | 0.587 |
|  | Eswatini | 0.586 |
|  | Tuvalu | 0.579 |
|  | India | 0.578 |
|  | Marshall Islands | 0.574 |
|  | Democratic People's Republic of Korea | 0.569 |
|  | El Salvador | 0.566 |
|  | Ghana | 0.563 |
|  | Morocco | 0.562 |
|  | Sudan | 0.543 |
|  | Guatemala | 0.540 |
|  | Tajikistan | 0.537 |
|  | Cabo Verde | 0.534 |
|  | Myanmar | 0.528 |
|  | Kiribati | 0.526 |
|  | Kenya | 0.525 |
|  | Nicaragua | 0.524 |
|  | Honduras | 0.514 |
|  | Lesotho | 0.512 |
|  | Zambia | 0.510 |
|  | Pakistan | 0.504 |
|  | Nigeria | 0.504 |
|  | Sao Tome and Principe | 0.503 |
|  | Mauritania | 0.495 |
|  | Bangladesh | 0.493 |
|  | Lao People's Democratic Republic | 0.489 |
|  | Djibouti | 0.489 |
|  | Angola | 0.483 |
|  | Cameroon | 0.480 |
|  | Comoros | 0.477 |
|  | Bhutan | 0.477 |
|  | Zimbabwe | 0.476 |
|  | Cambodia | 0.474 |
|  | Vanuatu | 0.473 |
| Low SDI region |  |  |
|  | Yemen | 0.454 |
|  | Timor-Leste | 0.451 |
|  | Haiti | 0.449 |
|  | United Republic of Tanzania | 0.449 |
|  | Rwanda | 0.436 |
|  | Nepal | 0.434 |
|  | Solomon Islands | 0.430 |
|  | Uganda | 0.427 |
|  | Côte d'Ivoire | 0.425 |
|  | Papua New Guinea | 0.418 |
|  | Gambia | 0.410 |
|  | Togo | 0.410 |
|  | Senegal | 0.409 |
|  | Eritrea | 0.405 |
|  | Madagascar | 0.401 |
|  | Democratic Republic of the Congo | 0.390 |
|  | Malawi | 0.382 |
|  | Benin | 0.375 |
|  | Ethiopia | 0.361 |
|  | Sierra Leone | 0.359 |
|  | Guinea-Bissau | 0.353 |
|  | Liberia | 0.353 |
|  | Guinea | 0.337 |
|  | Afghanistan | 0.335 |
|  | Mozambique | 0.327 |
|  | Central African Republic | 0.311 |
|  | Burundi | 0.291 |
|  | Burkina Faso | 0.284 |
|  | South Sudan | 0.278 |
|  | Mali | 0.271 |
|  | Chad | 0.244 |
|  | Niger | 0.170 |
|  | Somalia | 0.077 |

Abbreviation: SDI, socio-demographic index.
