# Supplementary material for: Long-term spatio-temporal trends in burden of fungal skin diseases in middle-aged and elderly people from 1990 to 2021
Source: PLoS Negl Trop Dis. 2026 Apr 1;20(4):e0014157. doi: 10.1371/journal.pntd.0014157 (PMC13065042; doi:10.1371/journal.pntd.0014157)
Supplement: S3 Table — (DOCX) [file pntd.0014157.s003.docx]

**S3 Table. Prevent cases and prevalence rate of fungal skin diseases in middle-aged and elderly people in 1990 and 2021, and temporal trends from 1990 to 2021.**

| Characteristic | 1990 | |  | 2021 | |  | 1990-2021 | |
| --- | --- | --- | --- | --- | --- | --- | --- | --- |
|  | Prevent cases | Prevalence rate per 100000 |  | Prevent cases | Prevalence rate per 100000 |  | Cases change | AAPC |
|  | No. (95% UI) | No. (95% UI) |  | No. (95% UI) | No. (95% UI) |  | % (95% UI) | % (95% CI) |
| Global | 80796031(70744457,93330520) | 12033.5(10536.46,13900.35) |  | 181089158(159315707,208484706) | 12186.46(10721.21,14030.06) |  | 124.13(121.41,127.27) | 4.18(3.11,5.24)* |
| Sex |  |  |  |  |  |  |  |  |
| Male | 36581287(31862864,42781185) | 11744.22(10229.39,13734.66) |  | 83990475(73658893,97497970) | 12006.91(10529.95,13937.88) |  | 129.6(125.99,133.6) | 7.26(6.13,8.39)* |
| Female | 44214744(38801899,51296405) | 12283.85(10780.04,14251.29) |  | 97098683(85951206,111651008) | 12346.17(10928.76,14196.5) |  | 119.61(116.77,122.99) | 1.66(0.7,2.62)* |
| Age groups |  |  |  |  |  |  |  |  |
| 55-59 years | 14373224(11850614,17640628) | 7760.92(6398.82,9525.18) |  | 30502018(25218636,37375840) | 7707.82(6372.72,9444.83) |  | 112.21(110.8,113.59) | -2.22(-4.1,-0.34)* |
| 60-64 years | 13628317(10388319,18047661) | 8485.39(6468.07,11237) |  | 26822786(20447217,35433145) | 8380.87(6388.8,11071.2) |  | 96.82(95.58,98.09) | -4.11(-5.41,-2.8)* |
| 65-69 years | 13121756(10500695,16394301) | 10615.49(8495.06,13262.98) |  | 27683322(22059955,34485085) | 10035.93(7997.31,12501.75) |  | 110.97(109.24,112.88) | -18.12(-20.62,-15.61)* |
| 70-74 years | 12078651(9798073,14745987) | 14267.05(11573.28,17417.65) |  | 28182763(22857973,34500040) | 13691.64(11104.77,16760.67) |  | 133.33(132.24,134.47) | -13.03(-15.47,-10.59)* |
| 75-79 years | 11342502(8526500,14494799) | 18426.46(13851.72,23547.52) |  | 22635198(16973048,28967187) | 17162.91(12869.64,21964.07) |  | 99.56(97.64,101.84) | -22.71(-24.66,-20.76)* |
| 80-84 years | 8842446(7276317,10742333) | 24995.66(20568.55,30366.22) |  | 20615072(16918110,25085794) | 23537.74(19316.65,28642.29) |  | 133.14(130.89,135.19) | -19.48(-21.58,-17.37)* |
| 85+ years | 7409134(6278162,8597181) | 36293.76(30753.68,42113.43) |  | 24647999(20731594,28796696) | 35689.94(30019.04,41697.19) |  | 232.67(228.21,237.55) | -5.07(-6.28,-3.87)* |
| SDI regions |  |  |  |  |  |  |  |  |
| Low SDI | 6009287(5151227,6992603) | 16107.23(13807.29,18742.9) |  | 13190537(11366677,15295323) | 16074.65(13852.01,18639.65) |  | 119.5(117.74,121.23) | -0.69(-1.69,0.31) |
| Low-middle SDI | 11392313(9870030,13309880) | 11301.8(9791.61,13204.14) |  | 27992652(24290860,32760559) | 11611.21(10075.72,13588.91) |  | 145.72(143.23,148.48) | 8.83(8.03,9.63)* |
| Middle SDI | 18170879(15700747,21300049) | 10469.48(9046.27,12272.4) |  | 51820286(45018368,60426483) | 11028.91(9581.26,12860.57) |  | 185.18(181.67,189.51) | 16.91(15.31,18.5)* |
| High-middle SDI | 19964327(17371177,23281436) | 11571.86(10068.8,13494.55) |  | 38999386(34157461,45318243) | 11249.48(9852.81,13072.17) |  | 95.35(91.9,99.05) | -9.07(-11.18,-6.96)* |
| High SDI | 25146023(22254320,28926409) | 13485.77(11934.96,15513.19) |  | 48878358(43689847,55304767) | 14167.1(12663.24,16029.76) |  | 94.38(90.28,98.65) | 15.75(13.56,17.94)* |
| GBD regions |  |  |  |  |  |  |  |  |
| Andean Latin America | 860683(741510,999920) | 25646.96(22095.79,29796) |  | 2612665(2253957,3031575) | 26373.42(22752.45,30602.08) |  | 203.56(200.74,206.39) | 9.09(8.34,9.84)* |
| Australasia | 895176(849066,949825) | 22723.02(21552.58,24110.23) |  | 2166675(2057958,2285294) | 24525.86(23295.22,25868.57) |  | 142.04(140.2,143.92) | 24.21(21.86,26.57)* |
| Caribbean | 839522(718619,971123) | 19479.69(16674.35,22533.29) |  | 1840586(1588734,2122312) | 19880.04(17159.81,22922.95) |  | 119.24(116.31,122.25) | 6.58(5.35,7.81)* |
| Central Asia | 1014700(877289,1196865) | 12686.85(10968.79,14964.47) |  | 1740019(1494260,2055775) | 11959.07(10269.98,14129.24) |  | 71.48(67.59,75.3) | -19.82(-24.73,-14.9)* |
| Central Europe | 3446775(2956686,4074792) | 12996.71(11148.74,15364.77) |  | 5510063(4768378,6494792) | 14880.8(12877.76,17540.22) |  | 59.86(54.99,64.9) | 44.03(41.84,46.23)* |
| Central Latin America | 1575679(1354430,1850715) | 11611.39(9980.98,13638.17) |  | 5547998(4815434,6480329) | 12972.85(11259.9,15152.91) |  | 252.1(239.27,263.85) | 35.24(33.47,37.01)* |
| Central Sub-Saharan Africa | 479778(409326,565879) | 12759.05(10885.46,15048.76) |  | 1159994(993646,1361088) | 12855.22(11011.74,15083.77) |  | 141.78(139.49,144.51) | 2.31(0.71,3.91)* |
| East Asia | 10182244(8730137,12118948) | 6835.88(5861,8136.09) |  | 28168115(24339607,33129671) | 7183.53(6207.17,8448.84) |  | 176.64(170.32,183.84) | 15.99(14.15,17.84)* |
| Eastern Europe | 6444147(5548557,7696447) | 13180.15(11348.41,15741.46) |  | 8786548(7590364,10389207) | 14153.82(12226.94,16735.46) |  | 36.35(34.15,39.06) | 22.1(16.87,27.32)* |
| Eastern Sub-Saharan Africa | 2616293(2219720,3067578) | 21505.61(18245.83,25215.11) |  | 5819397(4959670,6792806) | 21523.36(18343.61,25123.57) |  | 122.43(119.71,125.09) | 0.02(-0.86,0.89) |
| High-income Asia Pacific | 4916987(4253110,5753489) | 14061.3(12162.78,16453.47) |  | 12898593(11210379,14880081) | 18295(15900.49,21105.49) |  | 162.33(147.17,177.27) | 85.94(82.01,89.87)* |
| High-income North America | 3945337(3708710,4256138) | 6810.75(6402.27,7347.28) |  | 7572667(7164093,8102231) | 6729.21(6366.14,7199.79) |  | 91.94(88.9,95.06) | -4.08(-5.56,-2.6)* |
| North Africa and Middle East | 2164733(1852811,2540886) | 7659.03(6555.42,8989.89) |  | 5960543(5122434,7000517) | 7818.77(6719.37,9182.96) |  | 175.35(173.06,177.78) | 6.88(5.11,8.65)* |
| Oceania | 41260(34937,49061) | 8576.33(7262.15,10197.89) |  | 107577(91807,126987) | 8716.57(7438.83,10289.32) |  | 160.73(158.15,163.74) | 5.33(2.95,7.71)* |
| South Asia | 8932354(7621402,10566900) | 9408.19(8027.41,11129.82) |  | 24333489(20879517,28540837) | 9800.23(8409.16,11494.73) |  | 172.42(167.43,178.38) | 13.22(12.16,14.28)* |
| Southeast Asia | 6027796(5151053,7120306) | 14236.18(12165.53,16816.42) |  | 16356700(14014249,19308551) | 14278.51(12233.68,16855.32) |  | 171.35(170.58,172.32) | 0.96(0.45,1.48)* |
| Southern Latin America | 1109446(953411,1289365) | 14005.56(12035.78,16276.84) |  | 2224431(1919345,2566707) | 15115.63(13042.49,17441.49) |  | 100.5(96.76,104.2) | 24.63(23.56,25.7)* |
| Southern Sub-Saharan Africa | 820607(697267,954265) | 18545.72(15758.24,21566.4) |  | 1760358(1491989,2050544) | 18082.23(15325.57,21063.01) |  | 114.52(112.8,116.44) | -8.23(-10.25,-6.22)* |
| Tropical Latin America | 2803868(2394619,3275080) | 18517.96(15815.1,21630.05) |  | 8682955(7483118,10046683) | 19601.3(16892.73,22679.84) |  | 209.68(204.54,215.23) | 18.43(17.93,18.94)* |
| Western Europe | 18241762(15770166,21421186) | 18784.21(16239.12,22058.18) |  | 30499166(26629527,35180924) | 20450.78(17856.05,23590.07) |  | 67.19(63.45,70.85) | 27.35(25.94,28.76)* |
| Western Sub-Saharan Africa | 3436885(2941175,3993037) | 23808.57(20374.61,27661.25) |  | 7340620(6300200,8549687) | 22837.3(19600.46,26598.81) |  | 113.58(111.2,116) | -12.95(-13.59,-12.31)* |

Abbreviation: UI, uncertainty interval; AAPC, average annual percent change; CI, confidence interval; SDI, socio-demographic index.

Note: * indicates statistically significant.
