# Supplementary material for: Long-term spatio-temporal trends in burden of fungal skin diseases in middle-aged and elderly people from 1990 to 2021
Source: PLoS Negl Trop Dis. 2026 Apr 1;20(4):e0014157. doi: 10.1371/journal.pntd.0014157 (PMC13065042; doi:10.1371/journal.pntd.0014157)
Supplement: S4 Table — (DOCX) [file pntd.0014157.s004.docx]

**S4 Table. Number of DALYs and DALYs rate of fungal skin diseases in middle-aged and elderly people in 1990 and 2021, and temporal trends from 1990 to 2021.**

| Characteristic | 1990 | |  | 2021 | |  | 1990-2021 | |
| --- | --- | --- | --- | --- | --- | --- | --- | --- |
|  | DALYs | DALYs rate per 100000 |  | DALYs | DALYs rate per 100000 |  | Case change | AAPC |
|  | No. (95% UI) | No. (95% UI) |  | No. (95% UI) | No. (95% UI) |  | % (95% UI) | % (95% CI) |
| Global | 430374(174296,886604) | 64.1(25.96,132.05) |  | 960854(391476,1980242) | 64.66(26.34,133.26) |  | 123.26(120.61,126.24) | 2.89(1.89,3.89)* |
| Sex |  |  |  |  |  |  |  |  |
| Male | 196069(79491,401441) | 62.95(25.52,128.88) |  | 448907(183136,920409) | 64.17(26.18,131.58) |  | 128.95(125.47,132.77) | 6.27(4.91,7.63)* |
| Female | 234305(95188,485163) | 65.1(26.45,134.79) |  | 511947(207775,1059833) | 65.10(26.42,134.76) |  | 118.5(115.72,121.72) | -0.04(-1.01,0.93) |
| Age groups |  |  |  |  |  |  |  |  |
| 55-59 years | 78980(30989,170656) | 42.65(16.73,92.15) |  | 167668(66271,360694) | 42.37(16.75,91.15) |  | 112.29(110.59,113.92) | -2.07(-3.85,-0.29)* |
| 60-64 years | 74189(28890,162487) | 46.19(17.99,101.17) |  | 145977(57221,319015) | 45.61(17.88,99.68) |  | 96.77(95.25,98.25) | -4.04(-5.53,-2.54)* |
| 65-69 years | 70665(28219,152841) | 57.17(22.83,123.65) |  | 148996(59282,320893) | 54.01(21.49,116.33) |  | 110.85(108.93,112.94) | -18.26(-20.76,-15.76)* |
| 70-74 years | 64233(26853,137015) | 75.87(31.72,161.84) |  | 149772(62528,319527) | 72.76(30.38,155.23) |  | 133.17(131.91,134.56) | -13.12(-15.6,-10.64)* |
| 75-79 years | 59448(23514,127238) | 96.58(38.2,206.7) |  | 118523(46819,252032) | 89.87(35.5,191.1) |  | 99.37(97.31,101.63) | -23.39(-25.74,-21.04)* |
| 80-84 years | 45620(18700,94361) | 128.96(52.86,266.74) |  | 106328(43539,220759) | 121.4(49.71,252.06) |  | 133.07(130.77,135.19) | -19.64(-21.97,-17.3)* |
| 85+ years | 37239(14749,77110) | 182.42(72.25,377.73) |  | 123589(48704,257080) | 178.96(70.52,372.25) |  | 231.88(227.31,236.54) | -5.82(-7.31,-4.34)* |
| SDI regions |  |  |  |  |  |  |  |  |
| Low SDI | 31987(13077,66471) | 85.74(35.05,178.17) |  | 70340(28648,145313) | 85.72(34.91,177.09) |  | 119.9(117.98,121.69) | -0.18(-1.19,0.84) |
| Low-middle SDI | 60458(24184,125133) | 59.98(23.99,124.14) |  | 148322(59428,308975) | 61.52(24.65,128.16) |  | 145.33(142.65,148.16) | 8.29(7.58,9)* |
| Middle SDI | 97399(39205,201028) | 56.12(22.59,115.83) |  | 276233(111826,569119) | 58.79(23.8,121.13) |  | 183.61(180.32,187.8) | 15.01(13.52,16.5)* |
| High-middle SDI | 106378(43433,219156) | 61.66(25.18,127.03) |  | 207165(84348,428545) | 59.76(24.33,123.62) |  | 94.74(91.1,98.48) | -10(-12.28,-7.72)* |
| High SDI | 133549(54406,275090) | 71.62(29.18,147.53) |  | 257692(105383,528199) | 74.69(30.54,153.1) |  | 92.96(88.64,97.39) | 13.38(11.37,15.39)* |
| GBD regions |  |  |  |  |  |  |  |  |
| Andean Latin America | 4621(1872,9873) | 137.71(55.79,294.2) |  | 13962(5685,29654) | 140.94(57.39,299.34) |  | 202.13(198.6,205.61) | 7.57(6.58,8.57)* |
| Australasia | 4743(1966,9649) | 120.38(49.9,244.92) |  | 11423(4735,23042) | 129.3(53.6,260.83) |  | 140.86(138.61,143.51) | 22.63(20.66,24.59)* |
| Caribbean | 4506(1831,9596) | 104.55(42.49,222.66) |  | 9784(3966,20732) | 105.67(42.84,223.92) |  | 117.13(114.42,120.43) | 3.46(2.29,4.62)* |
| Central Asia | 5432(2239,11142) | 67.91(27.99,139.31) |  | 9319(3818,18997) | 64.05(26.24,130.57) |  | 71.57(67.61,75.54) | -19.44(-23.9,-14.98)* |
| Central Europe | 18220(7470,37258) | 68.7(28.17,140.49) |  | 29031(12049,60056) | 78.4(32.54,162.19) |  | 59.33(54.26,64.33) | 42.8(40.82,44.79)* |
| Central Latin America | 8359(3446,18125) | 61.6(25.39,133.56) |  | 29378(11954,61648) | 68.69(27.95,144.15) |  | 251.44(238.88,263.7) | 34.36(31.93,36.79)* |
| Central Sub-Saharan Africa | 2553(1040,5258) | 67.89(27.65,139.84) |  | 6184(2504,12745) | 68.53(27.75,141.25) |  | 142.22(138.55,145.97) | 2.93(1.2,4.67)* |
| East Asia | 55123(22102,114521) | 37.01(14.84,76.88) |  | 151246(61594,313871) | 38.57(15.71,80.04) |  | 174.38(168.36,181.61) | 13.51(11.9,15.13)* |
| Eastern Europe | 34063(13969,70198) | 69.67(28.57,143.57) |  | 46256(18977,96000) | 74.51(30.57,154.64) |  | 35.8(33.57,38.34) | 21.14(16.08,26.2)* |
| Eastern Sub-Saharan Africa | 13974(5738,29496) | 114.87(47.17,242.46) |  | 31131(12689,64882) | 115.14(46.93,239.97) |  | 122.77(119.76,125.41) | 0.7(-0.37,1.78) |
| High-income Asia Pacific | 26324(10740,54148) | 75.28(30.71,154.85) |  | 68325(28324,141250) | 96.91(40.17,200.34) |  | 159.56(144.89,174.51) | 82.53(78.51,86.55)* |
| High-income North America | 20771(8593,41926) | 35.86(14.83,72.38) |  | 39277(16430,78250) | 34.9(14.6,69.53) |  | 89.09(85.88,92.72) | -8.96(-10.47,-7.45)* |
| North Africa and Middle East | 11555(4665,24447) | 40.88(16.51,86.49) |  | 31533(12795,66912) | 41.36(16.78,87.77) |  | 172.9(170.23,175.75) | 3.99(2.41,5.56)* |
| Oceania | 222(88,469) | 46.12(18.33,97.51) |  | 576(228,1206) | 46.69(18.44,97.74) |  | 159.71(155.55,164.33) | 3.79(2.16,5.43)* |
| South Asia | 47092(18836,98090) | 49.6(19.84,103.32) |  | 128135(51528,269492) | 51.61(20.75,108.54) |  | 172.1(167.35,177.86) | 12.76(11.17,14.36)* |
| Southeast Asia | 32463(12877,69269) | 76.67(30.41,163.6) |  | 88045(35002,187073) | 76.86(30.55,163.3) |  | 171.22(169.71,172.79) | 0.9(0.18,1.63)* |
| Southern Latin America | 5929(2425,12384) | 74.85(30.62,156.34) |  | 11791(4803,24754) | 80.12(32.64,168.21) |  | 98.86(94.87,102.74) | 22.06(20.85,23.26)* |
| Southern Sub-Saharan Africa | 4371(1778,9067) | 98.79(40.19,204.91) |  | 9307(3796,19371) | 95.6(38.99,198.97) |  | 112.9(110.88,114.97) | -10.65(-11.82,-9.47)* |
| Tropical Latin America | 14816(6058,31346) | 97.85(40.01,207.02) |  | 45686(18507,96712) | 103.13(41.78,218.32) |  | 208.36(203.21,213.61) | 16.94(16.03,17.85)* |
| Western Europe | 96908(39563,203403) | 99.79(40.74,209.45) |  | 161114(65884,336220) | 108.03(44.18,225.45) |  | 66.25(62.56,70.08) | 25.44(23.91,26.97)* |
| Western Sub-Saharan Africa | 18329(7545,38249) | 126.97(52.27,264.97) |  | 39351(16199,82282) | 122.42(50.4,255.99) |  | 114.69(112.29,117.02) | -11.69(-13.44,-9.93)* |

Abbreviation: DALYs, disability-adjusted life-years; UI, uncertainty interval; AAPC, average annual percent change; CI, confidence interval; SDI, socio-demographic index.

Note: * indicates statistically significant.
