# Supplementary material for: Long-term spatio-temporal trends in burden of fungal skin diseases in middle-aged and elderly people from 1990 to 2021
Source: PLoS Negl Trop Dis. 2026 Apr 1;20(4):e0014157. doi: 10.1371/journal.pntd.0014157 (PMC13065042; doi:10.1371/journal.pntd.0014157)
Supplement: S5 Table — (DOCX) [file pntd.0014157.s005.docx]

**S5 Table. The burden of fungal skin diseases in middle-aged and elderly people and the entire population from 1990 to 2021.**

| Year | Incidence rate per 100000  No. (95% UI) | |  | Prevalence rate per 100000  No. (95% UI) | |  | DALYs rate per 100000  No. (95% UI) | |
| --- | --- | --- | --- | --- | --- | --- | --- | --- |
|  | Entire population | 55+ years |  | Entire population | 55+ years |  | Entire population | 55+ years |
| 1990 | 19306.3(17381.29,21232.55) | 37782.19(32232.45,44403.43) |  | 6891.78(6215.55,7674.33) | 12033.5(10536.46,13900.35) |  | 38.56(15.8,79.66) | 64.1(25.96,132.05) |
| 1991 | 19376.22(17443.28,21308.32) | 37858.48(32295.09,44486.11) |  | 6922.91(6244.61,7706.43) | 12054.66(10557.35,13923.04) |  | 38.73(15.87,80.01) | 64.22(26.02,132.35) |
| 1992 | 19449.13(17508.42,21384.8) | 37930.61(32355.12,44563.09) |  | 6954.99(6274.33,7736.55) | 12074.16(10576.6,13944.73) |  | 38.91(15.94,80.3) | 64.33(26.06,132.51) |
| 1993 | 19522.7(17574.54,21459.89) | 38006.37(32417.12,44645.64) |  | 6986.51(6303.66,7765.47) | 12095.19(10596.73,13968.86) |  | 39.09(16.01,80.6) | 64.45(26.11,132.73) |
| 1994 | 19595.82(17641.89,21531.07) | 38095.05(32487.25,44744.06) |  | 7015.47(6330.55,7791.35) | 12121.61(10620.77,13999.81) |  | 39.25(16.07,80.89) | 64.58(26.17,133.05) |
| 1995 | 19668.66(17708.66,21600.31) | 38183.53(32557.07,44843.82) |  | 7043.05(6356.31,7815.86) | 12149.53(10645.47,14032.83) |  | 39.4(16.12,81.16) | 64.73(26.21,133.32) |
| 1996 | 19746.64(17782.9,21678.17) | 38272.45(32621.54,44951.65) |  | 7073.48(6383.81,7847.1) | 12178.41(10670.92,14067.1) |  | 39.56(16.19,81.45) | 64.87(26.26,133.63) |
| 1997 | 19827.94(17865.43,21766.47) | 38367.6(32689.28,45068.29) |  | 7105.2(6410.87,7879.55) | 12209.88(10698.45,14104.56) |  | 39.74(16.27,81.8) | 65.01(26.32,133.94) |
| 1998 | 19909.01(17948.78,21853.9) | 38470.28(32764.67,45190.76) |  | 7136.08(6437.31,7911.06) | 12244.19(10728.77,14153.12) |  | 39.9(16.34,82.14) | 65.17(26.39,134.26) |
| 1999 | 19991.29(18033.9,21936.92) | 38591.05(32858.93,45305.12) |  | 7166.79(6463.98,7942.54) | 12284.23(10764.33,14214.78) |  | 40.07(16.41,82.46) | 65.36(26.48,134.71) |
| 2000 | 20075.08(18121.59,22024.9) | 38725.12(32966.1,45416) |  | 7198.12(6492.19,7975.11) | 12327.41(10803.09,14281.1) |  | 40.24(16.47,82.77) | 65.57(26.59,135.29) |
| 2001 | 20154.29(18206.19,22106.5) | 38830.88(33056.53,45491.01) |  | 7228.79(6521.46,8005) | 12360.44(10825.56,14334.44) |  | 40.4(16.54,83.12) | 65.73(26.7,135.57) |
| 2002 | 20224.19(18282.49,22176.59) | 38838.31(33069.67,45480.97) |  | 7257.19(6548.6,8032.08) | 12359.79(10820.01,14343.98) |  | 40.56(16.6,83.35) | 65.72(26.72,135.58) |
| 2003 | 20289.04(18354.72,22240.72) | 38837.8(33081.62,45470.23) |  | 7283.86(6574.32,8057.05) | 12357.11(10813.34,14349.98) |  | 40.7(16.66,83.69) | 65.7(26.73,135.63) |
| 2004 | 20357.37(18425.5,22310.13) | 38774.77(33051.12,45357.1) |  | 7310.58(6600.63,8082.2) | 12334.63(10791.16,14325.64) |  | 40.85(16.71,83.95) | 65.58(26.7,135.31) |
| 2005 | 20435.93(18499.78,22392.38) | 38782.89(33078.83,45313.25) |  | 7338.33(6629.13,8108.89) | 12337.05(10797.7,14322.54) |  | 41(16.77,84.22) | 65.59(26.73,135.34) |
| 2006 | 20523.43(18578,22481.9) | 38733.58(33056.3,45220.44) |  | 7364.54(6655.72,8140.89) | 12321.36(10790.99,14296.41) |  | 41.14(16.83,84.46) | 65.51(26.71,135.09) |
| 2007 | 20613.87(18657.95,22576.63) | 38683.66(33028.09,45149.21) |  | 7387.95(6679.15,8170.06) | 12305.59(10782.19,14271.5) |  | 41.26(16.89,84.75) | 65.43(26.68,134.93) |
| 2008 | 20703.68(18736,22671.9) | 38607.44(32981.9,45035.42) |  | 7410.1(6701.56,8197.42) | 12281.67(10767.02,14236.27) |  | 41.38(16.94,85.01) | 65.31(26.64,134.77) |
| 2009 | 20794.52(18815.77,22772.17) | 38517.91(32918.17,44892.97) |  | 7434.26(6723.95,8226.63) | 12254.03(10748.5,14194.56) |  | 41.51(17,85.26) | 65.17(26.59,134.35) |
| 2010 | 20886.74(18896.79,22880.29) | 38420.92(32857.22,44740.28) |  | 7462.99(6752.23,8258.08) | 12224.95(10731.37,14139.08) |  | 41.67(17.07,85.6) | 65.01(26.53,134.01) |
| 2011 | 20978.11(18974.99,22985.49) | 38354.66(32807.54,44630.71) |  | 7494.82(6784.16,8293.05) | 12205.3(10719.66,14099.24) |  | 41.84(17.14,85.96) | 64.91(26.49,133.86) |
| 2012 | 21069.22(19052.97,23083.62) | 38336.44(32795.92,44600.77) |  | 7527.64(6814.34,8324.35) | 12201.03(10719.22,14083.53) |  | 42.01(17.22,86.34) | 64.88(26.47,133.77) |
| 2013 | 21156.52(19127.32,23177.5) | 38334.31(32798.82,44600.15) |  | 7559.99(6844.25,8355.25) | 12202.71(10723.46,14076.02) |  | 42.18(17.28,86.66) | 64.89(26.47,133.82) |
| 2014 | 21242.64(19209.46,23269.99) | 38345.88(32823.72,44620.68) |  | 7591.9(6872.37,8386.07) | 12209.04(10730.48,14077.35) |  | 42.35(17.36,87.06) | 64.92(26.47,133.91) |
| 2015 | 21324.55(19289.78,23357.66) | 38336.42(32838.3,44622) |  | 7621.49(6899.02,8415.14) | 12208.9(10733.16,14068.05) |  | 42.51(17.44,87.41) | 64.91(26.46,133.88) |
| 2016 | 21403.96(19369.97,23442.5) | 38350.79(32869.78,44640.94) |  | 7649.18(6927.08,8445.13) | 12215.14(10739.45,14069.49) |  | 42.65(17.49,87.71) | 64.94(26.45,133.95) |
| 2017 | 21482.67(19449.24,23523.98) | 38384.66(32913.2,44669.52) |  | 7676.15(6956.68,8467.72) | 12227.45(10750.17,14082.75) |  | 42.79(17.56,88) | 64.99(26.48,134) |
| 2018 | 21565.1(19521.44,23608.13) | 38387.72(32930.25,44659.38) |  | 7703.46(6986.61,8490.7) | 12229.38(10752.94,14082.67) |  | 42.93(17.61,88.28) | 64.99(26.49,133.99) |
| 2019 | 21661.54(19597.59,23714.85) | 38382.12(32936.94,44630.4) |  | 7733.74(7013.41,8518.36) | 12227.71(10751.88,14080.71) |  | 43.08(17.68,88.58) | 64.97(26.48,133.88) |
| 2020 | 21751.01(19662.44,23828.41) | 38322.35(32893.23,44561.6) |  | 7756.22(7025.97,8551.7) | 12207.49(10735.92,14053.48) |  | 43.17(17.74,88.7) | 64.82(26.42,133.56) |
| 2021 | 21912.9(19802.81,24009.39) | 38255.44(32832.61,44492.76) |  | 7812.76(7080.21,8607.84) | 12186.46(10721.21,14030.06) |  | 43.46(17.84,89.27) | 64.66(26.34,133.26) |

Abbreviation: UI, uncertainty interval.
