# Supplementary material for: Long-term spatio-temporal trends in burden of fungal skin diseases in middle-aged and elderly people from 1990 to 2021
Source: PLoS Negl Trop Dis. 2026 Apr 1;20(4):e0014157. doi: 10.1371/journal.pntd.0014157 (PMC13065042; doi:10.1371/journal.pntd.0014157)
Supplement: S6 Table — (DOCX) [file pntd.0014157.s006.docx]

**S6 Table. Incident cases and incidence rate of fungal skin diseases in middle-aged and elderly people across 204 countries and territories in 1990 and 2021, and temporal trends from 1990 to 2021.**

| Characteristic | 1990 | |  | 2021 | |  | 1990-2021 | |
| --- | --- | --- | --- | --- | --- | --- | --- | --- |
|  | Incident cases | Incidence rate per 100000 |  | Incident cases | Incidence rate per 100000 |  | Cases change | AAPC |
|  | No. (95% UI) | No. (95% UI) |  | No. (95% UI) | No. (95% UI) |  | % (95% UI) | % (95% CI) |
| Afghanistan | 314962(264604,371605) | 25918.82(21774.78,30580.08) |  | 342860(289580,403858) | 27903.09(23566.99,32867.37) |  | 8.86(4.73,12.81) | 24.42(18.49,30.35)* |
| Albania | 144658(121610,168793) | 41685.12(35043.57,48640.19) |  | 354963(298537,415530) | 45143.83(37967.59,52846.7) |  | 145.38(139.96,151.72) | 25.91(24.42,27.4)* |
| Algeria | 546313(457450,648230) | 26076.61(21835,30941.32) |  | 1643041(1389515,1943066) | 27053.19(22878.82,31993.21) |  | 200.75(195.33,208.25) | 12.13(10.9,13.35)* |
| American Samoa | 1073(903,1269) | 29329.71(24685.3,34697.2) |  | 2548(2158,2993) | 30173(25550.74,35438.12) |  | 137.61(134.67,140.83) | 9.63(8.41,10.85)* |
| Andorra | 5143(4305,6109) | 52116.69(43626.58,61914.51) |  | 14657(12422,17244) | 55391.18(46943.59,65165.86) |  | 185.02(177.87,194.65) | 19.35(10.93,27.79)* |
| Angola | 262597(219635,315901) | 42156.32(35259.49,50713.69) |  | 821143(690341,984920) | 42466.37(35701.81,50936.33) |  | 212.7(210.76,214.83) | 2.49(0.65,4.34)* |
| Antigua and Barbuda | 5525(4668,6514) | 63946.55(54021.79,75384.46) |  | 10459(8788,12541) | 55431.54(46579.4,66468.04) |  | 89.28(80.91,97.57) | -46.11(-47.1,-45.11)* |
| Argentina | 2552448(2167679,2984092) | 45869.32(38954.76,53626.27) |  | 4621543(3941597,5408900) | 49516.65(42231.51,57952.65) |  | 81.06(77.5,85) | 24.72(23.55,25.9)* |
| Armenia | 196692(166092,230244) | 40407.8(34121.35,47300.63) |  | 352833(299033,410787) | 44854.32(38014.93,52221.79) |  | 79.38(74.94,84.77) | 32.8(26.48,39.13)* |
| Australia | 1932824(1682938,2264547) | 58899.97(51285.07,69008.74) |  | 4596029(3990713,5364993) | 62042.88(53871.58,72423.3) |  | 137.79(133.9,142) | 16.29(14.4,18.18)* |
| Austria | 1127256(947281,1343379) | 57929.39(48680.54,69035.91) |  | 1767214(1497574,2088632) | 59800.68(50676.35,70677.15) |  | 56.77(54.17,60.01) | 10.33(6.69,13.98)* |
| Azerbaijan | 355891(300519,415216) | 40803.66(34455.17,47605.43) |  | 729560(614672,861553) | 38081.24(32084.36,44970.96) |  | 105(98.18,110.81) | -22.61(-26.95,-18.27)* |
| Bahamas | 14033(11801,16721) | 57150.22(48060.42,68096.73) |  | 39046(32882,46637) | 54261.22(45695.43,64810.97) |  | 178.23(171.61,184.4) | -16.82(-19.36,-14.29)* |
| Bahrain | 6572(5421,7807) | 23424.03(19319.66,27826.65) |  | 36126(29769,43168) | 22248.81(18333.87,26585.55) |  | 449.68(432.73,465.66) | -16.14(-21.31,-10.97)* |
| Bangladesh | 2581625(2206814,3041316) | 34082.38(29134.15,40151.18) |  | 8032641(6852450,9465145) | 34377.26(29326.4,40507.94) |  | 211.15(208.6,214.15) | 3.14(1.3,4.98)* |
| Barbados | 30520(25580,36168) | 65095.93(54559.67,77142.02) |  | 54161(45706,64101) | 59440.05(50160.6,70349.17) |  | 77.46(70.98,83.83) | -29.87(-31.98,-27.75)* |
| Belarus | 1008497(850478,1175013) | 43564.83(36738.77,50757.96) |  | 1309378(1112147,1521886) | 45518.22(38661.84,52905.67) |  | 29.83(28.55,31.25) | 14.37(10.32,18.43)* |
| Belgium | 1476786(1241929,1757025) | 56324.58(47367.12,67012.9) |  | 2282622(1933093,2693798) | 60374.48(51129.57,71249.92) |  | 54.57(51.44,58.4) | 22.41(19.84,24.98)* |
| Belize | 9120(7713,10790) | 59251.65(50110.98,70097.92) |  | 27571(23261,33006) | 55047.58(46441.96,65898.91) |  | 202.31(194.8,209.47) | -23.82(-24.38,-23.27)* |
| Benin | 193706(164250,228607) | 60547.72(51340.48,71456.84) |  | 477755(405982,565510) | 57765.33(49087.26,68375.83) |  | 146.64(141.61,151.23) | -13.42(-15.27,-11.57)* |
| Bermuda | 6076(5102,7241) | 57432.28(48227.01,68446.14) |  | 14489(12294,17123) | 62210.66(52783.85,73517.03) |  | 138.49(133.28,144.06) | 25.82(23.81,27.83)* |
| Bhutan | 12457(10495,14804) | 31389.25(26444.92,37304.56) |  | 35196(30079,41194) | 35341.27(30202.31,41363.68) |  | 182.54(171.57,196.98) | 38.38(37.27,39.48)* |
| Bolivia (Plurinational State of) | 328259(274600,390819) | 62605.33(52371.41,74536.57) |  | 977332(816731,1161014) | 63238.43(52846.69,75123.63) |  | 197.73(196.28,199.29) | 3.44(1.79,5.09)* |
| Bosnia and Herzegovina | 289503(243685,339485) | 39466.44(33220.26,46280.22) |  | 508988(430819,592020) | 46471.49(39334.58,54052.5) |  | 75.81(68.48,84.95) | 53.26(49.46,57.06)* |
| Botswana | 48190(40353,57712) | 51834.63(43405.01,62076.03) |  | 126689(106403,151206) | 52259.35(43891.34,62372.61) |  | 162.89(160.46,166.29) | 3.69(-0.35,7.73) |
| Brazil | 8334766(6971180,9795343) | 56397.64(47170.86,66280.71) |  | 25753361(21750368,30029440) | 59465.94(50222.81,69339.65) |  | 208.99(204.01,214.92) | 17.14(16.7,17.58)* |
| Brunei Darussalam | 6791(5785,7930) | 43374.8(36949.22,50651.41) |  | 23630(19983,28044) | 39466.17(33375.1,46837.68) |  | 247.96(237.24,257.39) | -31.45(-36.09,-26.8)* |
| Bulgaria | 944457(787495,1116806) | 41729.41(34794.27,49344.36) |  | 1173237(985642,1377826) | 49359.8(41467.39,57967.15) |  | 24.22(18.96,30.32) | 54.64(51.83,57.46)* |
| Burkina Faso | 412624(345894,490863) | 57052.29(47825.69,67870.1) |  | 869296(736118,1030427) | 57821.43(48963.08,68539.14) |  | 110.68(109.29,112.43) | 4.6(2.94,6.26)* |
| Burundi | 220569(184561,259279) | 58607.85(49040.08,68893.43) |  | 446010(371465,529245) | 55714.49(46402.51,66112.01) |  | 102.21(94.91,108.5) | -16.69(-18.56,-14.81)* |
| Cabo Verde | 25484(21625,29896) | 64566.1(54789.96,75746.15) |  | 45574(38554,53449) | 59459.99(50301.65,69735.14) |  | 78.84(70.71,86.66) | -27.03(-31.46,-22.6)* |
| Cambodia | 287731(240900,345945) | 38834.24(32513.6,46691.16) |  | 842532(707233,1010756) | 38758.29(32534.26,46496.94) |  | 192.82(191.74,193.85) | -0.66(-1.31,-0.02)* |
| Cameroon | 399951(333780,476163) | 54015.02(45078.39,64307.76) |  | 1098694(917785,1307823) | 53977.21(45089.42,64251.42) |  | 174.71(174.22,175.19) | -0.11(-3.09,2.86) |
| Canada | 1258240(1072030,1477913) | 23048.85(19637.79,27072.88) |  | 2991441(2538122,3486261) | 24377.77(20683.59,28410.14) |  | 137.75(134.18,142.14) | 17.7(14.8,20.61)* |
| Central African Republic | 78359(65282,94619) | 41501.11(34575.01,50112.9) |  | 145869(121718,176618) | 40779.93(34028.17,49376.21) |  | 86.15(84.31,87.68) | -5.68(-7.87,-3.5)* |
| Chad | 278668(235883,329881) | 60134.88(50902.08,71186.21) |  | 526220(442955,624076) | 56672.81(47705.37,67211.71) |  | 88.83(83.55,93.65) | -19.08(-24.38,-13.77)* |
| Chile | 759927(646115,886441) | 45187.56(38419.92,52710.46) |  | 2133974(1826774,2484931) | 47658(40797.31,55495.91) |  | 180.81(176.64,185.98) | 17.26(15.91,18.61)* |
| China | 33038718(27730723,39489405) | 23020.61(19322.12,27515.29) |  | 91947079(78103337,109515584) | 24263.32(20610.18,28899.35) |  | 178.3(171.64,186.51) | 17(15,19)* |
| Colombia | 1176955(1004456,1374285) | 40880.82(34889.19,47734.97) |  | 4214707(3625899,4939892) | 44070.75(37913.92,51653.59) |  | 258.1(250.82,266) | 24.32(23.69,24.94)* |
| Comoros | 18338(15255,21679) | 56504.04(47003.27,66796.63) |  | 47137(39442,55368) | 58316.13(48796.23,68498.85) |  | 157.04(153.23,161.3) | 11.04(6.71,15.38)* |
| Congo | 77734(65124,92942) | 43667.75(36583.69,52210.78) |  | 187394(157159,224196) | 43125.81(36167.82,51595.28) |  | 141.07(137.65,144.89) | -4.01(-6.39,-1.62)* |
| Cook Islands | 649(551,761) | 30396.27(25798.02,35654.54) |  | 1512(1293,1763) | 32072.85(27434.45,37415.37) |  | 133.03(127.72,139.05) | 16.7(12.77,20.64)* |
| Costa Rica | 123128(105266,143729) | 43294.76(37013.82,50538.57) |  | 418085(358734,489870) | 43665.91(37467.15,51163.29) |  | 239.55(236.25,243.16) | 2.58(1.63,3.52)* |
| Croatia | 461424(388504,539531) | 41450.93(34900.32,48467.47) |  | 743817(628950,865161) | 49876.22(42173.88,58012.86) |  | 61.2(53.09,70.67) | 60.42(56.11,64.73)* |
| Cuba | 1012950(855637,1202552) | 59771.65(50488.99,70959.61) |  | 2051547(1742576,2421554) | 60038.6(50996.56,70866.87) |  | 102.53(99.78,106.06) | 1.58(-0.81,3.96) |
| Cyprus | 72921(60572,87734) | 52201.8(43361.68,62806.62) |  | 194685(163412,231161) | 55703.59(46755.67,66140.29) |  | 166.98(160.77,175.76) | 20.97(19.73,22.21)* |
| Czechia | 1081167(906865,1260690) | 45660.43(38299.22,53242.15) |  | 1754796(1475243,2060255) | 50230.83(42228.67,58974.57) |  | 62.31(58.07,66.99) | 30.51(27.64,33.38)* |
| CÔTE D'IVOIRE | 344202(287481,408427) | 53703.41(44853.57,63723.91) |  | 990109(833100,1172916) | 55215.2(46459.27,65409.76) |  | 187.65(184.5,191.67) | 9.42(8.1,10.74)* |
| Democratic People's Republic of Korea | 572820(485283,669520) | 21523.45(18234.28,25156.91) |  | 1288847(1099225,1507515) | 22869.05(19504.44,26749.06) |  | 125(118.35,132.33) | 19.67(17.96,21.38)* |
| Democratic Republic of the Congo | 1113730(931077,1338149) | 42174.91(35258.17,50673.22) |  | 2589010(2179929,3102851) | 42865.71(36092.64,51373.28) |  | 132.46(129.92,135.2) | 5.63(3.2,8.05)* |
| Denmark | 772107(650061,917391) | 59181.91(49827.16,70317.95) |  | 1147976(967745,1362481) | 59628.24(50266.67,70770.03) |  | 48.68(47.8,49.64) | 2.84(-1.2,6.89) |
| Djibouti | 11507(9587,13730) | 53636.16(44684.18,63998.21) |  | 56394(46943,67197) | 54380.3(45266.71,64796.67) |  | 390.08(387.11,392.96) | 4.71(3.47,5.95)* |
| Dominica | 5977(5025,7105) | 60881.63(51180.74,72365.46) |  | 8499(7160,10165) | 56350.62(47473.72,67392.87) |  | 42.19(37.32,47.05) | -24.96(-25.86,-24.07)* |
| Dominican Republic | 340358(285733,407785) | 55668.84(46734.39,66697.19) |  | 976454(826421,1157219) | 58434.46(49455.95,69252.11) |  | 186.89(182.79,191.6) | 15.61(13.48,17.74)* |
| Ecuador | 555946(468806,658146) | 64656.96(54522.53,76542.9) |  | 1824245(1539501,2154848) | 65916.24(55627.47,77862.09) |  | 228.13(225.14,231.23) | 6.92(4.34,9.49)* |
| Egypt | 1109406(920344,1320867) | 24811.14(20582.88,29540.32) |  | 2668618(2209091,3199995) | 24115.8(19963.14,28917.76) |  | 140.54(138.16,142.69) | -8.51(-10.63,-6.39)* |
| El Salvador | 212181(181722,248135) | 43394.47(37165.08,50747.68) |  | 475325(408684,555160) | 46482.76(39965.83,54289.97) |  | 124.02(119.86,128.47) | 22.15(19.22,25.08)* |
| Equatorial Guinea | 13898(11652,16638) | 43537.73(36503.66,52122.35) |  | 35323(29716,42040) | 44657.37(37569.25,53149.37) |  | 154.16(151.67,156.81) | 8.38(5.53,11.22)* |
| Eritrea | 90079(74955,108723) | 50972.75(42414.23,61522.42) |  | 237125(198349,284247) | 53817.54(45017,64512.37) |  | 163.24(157.05,171.02) | 17.61(14.79,20.44)* |
| Estonia | 160233(134964,186319) | 44698.99(37649.93,51975.93) |  | 227375(192669,265137) | 51974.45(44041,60606.27) |  | 41.9(37.2,47.01) | 49.05(45.48,52.62)* |
| Eswatini | 23840(20024,28281) | 52795.75(44345.96,62631.79) |  | 46734(39103,56047) | 51617.33(43189.31,61903.93) |  | 96.03(93.86,98.15) | -6.92(-7.56,-6.29)* |
| Ethiopia | 2041651(1696757,2454899) | 63543.81(52809.42,76405.65) |  | 4614961(3899275,5457206) | 67415.23(56960.49,79718.72) |  | 126.04(118.01,133.97) | 18.85(17.14,20.56)* |
| Fiji | 16526(13923,19473) | 29319.68(24701.83,34548.78) |  | 39830(33492,47037) | 29128.22(24493.43,34398.66) |  | 141.02(139.81,142.15) | -1.99(-2.99,-1)* |
| Finland | 670681(562732,800921) | 56485.42(47393.81,67454.37) |  | 1241227(1049871,1472626) | 61670.74(52163.17,73167.89) |  | 85.07(80.78,90.11) | 27(23.58,30.43)* |
| France | 8013319(6744677,9530185) | 57581.16(48465.1,68480.87) |  | 13719491(11666768,16190068) | 62050.72(52766.64,73224.69) |  | 71.21(67.75,75.32) | 24.31(23.09,25.54)* |
| Gabon | 45764(38237,53720) | 46744.5(39056.12,54870.99) |  | 78655(65788,92883) | 44002.06(36804.33,51961.93) |  | 71.87(67.35,75.68) | -19.13(-24.25,-14)* |
| Gambia | 31781(26616,37737) | 57389.1(48063.03,68143.98) |  | 90896(77138,107472) | 58778.98(49881.9,69497.94) |  | 186.01(182.97,189.44) | 7.64(5.29,9.99)* |
| Georgia | 475136(400459,554477) | 42776.27(36053.12,49919.32) |  | 500053(426729,579112) | 47630.07(40645.94,55160.4) |  | 5.24(2.63,8.08) | 34.8(30.86,38.74)* |
| Germany | 12033155(10119742,14336830) | 57346.63(48227.84,68325.29) |  | 19197581(16287914,22665492) | 60917.82(51684.85,71922.2) |  | 59.54(56.52,63.35) | 18.98(14.31,23.64)* |
| Ghana | 390451(331470,453425) | 38234.46(32458.8,44401.09) |  | 1054191(895204,1222455) | 38655.11(32825.36,44825.01) |  | 169.99(168.62,171.46) | 3.91(2.69,5.13)* |
| Greece | 1446493(1214543,1716927) | 54101.42(45426.1,64216.13) |  | 2350523(2000548,2768748) | 63951.98(54430.03,75330.86) |  | 62.5(55.52,70.23) | 54.52(52.05,56.99)* |
| Greenland | 1010(848,1197) | 18323.32(15393.48,21716.75) |  | 2509(2109,2983) | 18477.56(15532.04,21969.9) |  | 148.45(146.33,150.02) | 2.31(-3.74,8.37) |
| Grenada | 7711(6534,9086) | 63943.18(54181.7,75346.59) |  | 10977(9183,13167) | 54648.11(45714.35,65547.24) |  | 42.36(35.57,48.89) | -50(-53.16,-46.85)* |
| Guam | 3702(3106,4404) | 28712.2(24088.58,34155) |  | 12405(10595,14460) | 32916.35(28114.72,38370.45) |  | 235.08(219.11,254.44) | 44.29(41.65,46.94)* |
| Guatemala | 222282(187538,260503) | 38651.8(32610.39,45298.02) |  | 778135(665689,908506) | 42503.1(36361.1,49624.19) |  | 250.07(239.92,260.93) | 30.61(28.38,32.85)* |
| Guinea | 328782(278100,389765) | 59411.9(50253.44,70431.76) |  | 534674(453958,631682) | 58526.53(49691.24,69145.25) |  | 62.62(61.22,63.91) | -4.51(-5.91,-3.11)* |
| Guinea-Bissau | 35838(29913,42615) | 56143.27(46860.95,66759.74) |  | 62477(52167,74307) | 54389.24(45413.62,64687.86) |  | 74.33(71.18,77.17) | -10.32(-12.86,-7.78)* |
| Guyana | 33964(28469,40727) | 55408.14(46443.78,66440.92) |  | 60231(50522,72035) | 53482.83(44861.77,63964.78) |  | 77.34(74.9,79.59) | -11.11(-13.07,-9.15)* |
| Haiti | 281639(234930,338682) | 52217.82(43557.65,62794.02) |  | 617160(516275,741329) | 52077.15(43564.25,62554.79) |  | 119.13(118.03,120.26) | -0.77(-2.91,1.37) |
| Honduras | 136038(116177,158846) | 40813.46(34854.98,47656.27) |  | 427230(363213,499694) | 40624.01(34536.81,47514.39) |  | 214.05(212.2,215.5) | -1.83(-6.81,3.14) |
| Hungary | 1152168(965374,1347446) | 44732.01(37479.86,52313.48) |  | 1600213(1349613,1868669) | 50319.19(42439.02,58760.89) |  | 38.89(35.25,43.01) | 38.1(34.3,41.91)* |
| Iceland | 27131(22865,32131) | 56860.26(47920.37,67340.29) |  | 55999(47253,66136) | 57502.36(48520.81,67911.28) |  | 106.41(104.97,108.23) | 3.69(2.06,5.32)* |
| India | 23659161(19801040,27949269) | 30858.78(25826.61,36454.4) |  | 65935474(55720147,77370165) | 32796.32(27715.21,38483.93) |  | 178.69(172.24,187.07) | 20.05(17.94,22.17)* |
| Indonesia | 6490555(5493352,7702860) | 40175.01(34002.56,47678.89) |  | 16657790(14049100,19769170) | 39775.47(33546.44,47204.83) |  | 156.65(155.41,157.66) | -3.11(-4.45,-1.77)* |
| Iran (Islamic Republic of) | 1148695(960227,1370646) | 24917.81(20829.52,29732.44) |  | 3679750(3112043,4303303) | 28325.51(23955.48,33125.41) |  | 220.34(207.11,235.58) | 42.82(39.07,46.57)* |
| Iraq | 367335(313099,430704) | 29100.77(24804.19,34121) |  | 998860(838003,1180709) | 25709.41(21569.15,30389.98) |  | 171.92(163.17,180.76) | -39.69(-41.71,-37.67)* |
| Ireland | 381000(318805,454854) | 55969.85(46833.29,66819.2) |  | 749037(631326,888037) | 57075.18(48105.82,67666.79) |  | 96.6(93.67,100.32) | 5.94(4.42,7.46)* |
| Israel | 518941(436627,616064) | 64023.68(53868.29,76006.12) |  | 1332027(1126535,1571012) | 66649.87(56367.77,78607.82) |  | 156.68(152.64,161.87) | 13.33(11.02,15.64)* |
| Italy | 8764280(7381551,10261369) | 57587.42(48501.93,67424.34) |  | 14693548(12362110,17283685) | 64681.43(54418.37,76083.29) |  | 67.65(62.05,73.88) | 37.13(35.47,38.79)* |
| Jamaica | 182625(154195,216360) | 62005.94(52353.19,73459.99) |  | 318289(271305,376483) | 60180.4(51296.83,71183.25) |  | 74.29(69.49,79.1) | -9.54(-12.29,-6.79)* |
| Japan | 13895255(11733702,16506157) | 46922.42(39623.15,55739.09) |  | 32810601(27826074,38582593) | 62854.46(53305.73,73911.73) |  | 136.13(121.86,152.94) | 95.04(89.82,100.26)* |
| Jordan | 54685(45917,64375) | 25433.69(21355.72,29940.32) |  | 318839(268115,376061) | 25360.17(21325.62,29911.59) |  | 483.04(477.11,488.69) | -0.7(-4.72,3.32) |
| Kazakhstan | 883542(745026,1031978) | 42247.32(35624.04,49344.89) |  | 1279371(1075970,1500956) | 40314.88(33905.41,47297.37) |  | 44.8(42.55,46.8) | -15.43(-19.91,-10.94)* |
| Kenya | 715739(610493,837420) | 53703.11(45806.32,62833.04) |  | 1940987(1636279,2303605) | 51887.32(43741.74,61580.99) |  | 171.19(155.23,190.16) | -10.73(-11.73,-9.73)* |
| Kiribati | 1748(1471,2074) | 29339.66(24682.55,34812) |  | 3576(3002,4245) | 28430.25(23870.48,33749.45) |  | 104.56(99.95,107.97) | -10.16(-11.36,-8.97)* |
| Kuwait | 23081(19425,27222) | 25242.41(21244.2,29771.28) |  | 119616(101244,140740) | 25656.3(21715.83,30187.28) |  | 418.24(411.23,427.54) | 9.39(-15.02,33.87) |
| Kyrgyzstan | 218202(184003,254496) | 42376.19(35734.57,49424.72) |  | 333772(281772,392934) | 39464.65(33316.22,46459.8) |  | 52.96(48.56,56.74) | -23.11(-28.96,-17.26)* |
| Lao People's Democratic Republic | 133339(111676,160483) | 38881.86(32564.76,46796.84) |  | 303695(255310,363359) | 39280.22(33022.06,46997.31) |  | 127.76(125.57,130.86) | 3.45(2.61,4.29)* |
| Latvia | 281566(237793,326905) | 45033.47(38032.33,52284.93) |  | 336168(284964,391492) | 51087.78(43306.38,59495.47) |  | 19.39(16.01,23.32) | 40.21(38.26,42.15)* |
| Lebanon | 100431(84716,118520) | 26489.56(22344.54,31260.72) |  | 318601(271735,373869) | 32509.35(27727.29,38148.83) |  | 217.23(199.4,235.67) | 67.07(63.33,70.81)* |
| Lesotho | 77450(65163,91360) | 55385.26(46598.73,65331.96) |  | 95046(79582,113609) | 52641.37(44076.41,62922.54) |  | 22.72(20.18,25.1) | -16.1(-18.56,-13.63)* |
| Liberia | 111879(93961,133077) | 59371.3(49862.87,70620.73) |  | 185427(156797,219251) | 56169.2(47496.72,66415.21) |  | 65.74(60.38,70.64) | -18.75(-23.36,-14.13)* |
| Libya | 86863(73852,102073) | 27927.39(23744.13,32817.62) |  | 224118(190434,263883) | 26798.18(22770.51,31552.99) |  | 158.01(156.1,160.04) | -13.2(-16.93,-9.47)* |
| Lithuania | 356308(301600,413893) | 44962.91(38059.18,52229.6) |  | 489613(415939,569076) | 50591.34(42978.62,58802.18) |  | 37.41(33.65,41.83) | 38.25(34.19,42.31)* |
| Luxembourg | 51664(43408,61412) | 55322.89(46482.65,65761.16) |  | 100989(85446,119295) | 56830.79(48083.78,67131.83) |  | 95.47(92.23,99.64) | 8.58(6.36,10.81)* |
| Madagascar | 481117(400655,567307) | 56943.07(47419.86,67144.23) |  | 985371(819701,1175522) | 53359.05(44387.83,63655.95) |  | 104.81(99.07,109.91) | -20.7(-21.96,-19.44)* |
| Malawi | 354260(294157,420234) | 56323.01(46767.42,66812.13) |  | 668043(554960,788514) | 57140.28(47467.89,67444.62) |  | 88.57(86.96,90.35) | 4.98(2.92,7.04)* |
| Malaysia | 607476(513077,720779) | 41160.07(34763.98,48836.97) |  | 1973152(1663883,2361818) | 40245.49(33937.47,48172.95) |  | 224.81(220.43,228.25) | -7.17(-8.67,-5.68)* |
| Maldives | 5808(4829,7069) | 37888.51(31498.78,46112.32) |  | 21908(18534,26049) | 40274.42(34071.43,47886.88) |  | 277.19(265.7,291.24) | 19.9(18.02,21.79)* |
| Mali | 411678(342791,492718) | 61567.8(51265.57,73687.67) |  | 901269(752662,1074581) | 62269.28(52001.97,74243.52) |  | 118.93(117.55,120.44) | 3.65(2.34,4.96)* |
| Malta | 38818(32543,46102) | 53581.97(44920.7,63636.6) |  | 95022(80043,113053) | 60215.49(50723.17,71641.62) |  | 144.79(137.19,153.3) | 37.68(35.1,40.26)* |
| Marshall Islands | 769(649,909) | 30140.36(25438.58,35643.08) |  | 1649(1384,1973) | 28146.99(23614.56,33667.7) |  | 114.53(104.99,122.38) | -22.49(-25.09,-19.89)* |
| Mauritania | 98336(83153,116535) | 59234.4(50088.43,70196.74) |  | 210160(178199,248525) | 58579.95(49671.06,69273.85) |  | 113.72(110.38,116.89) | -4.47(-6.12,-2.82)* |
| Mauritius | 48099(40250,57670) | 39543.26(33090.37,47411.66) |  | 137809(115834,164187) | 40732(34236.66,48528.24) |  | 186.51(183.01,190.76) | 9.51(8.51,10.52)* |
| Mexico | 2700966(2291157,3161967) | 38847.51(32953.3,45478.01) |  | 9642120(8151297,11391889) | 44742.09(37824.27,52861.51) |  | 256.99(232.86,277.73) | 45.86(39.88,51.85)* |
| Micronesia (Federated States of) | 2442(2066,2875) | 30678.52(25953.21,36107.8) |  | 3738(3138,4446) | 28405.11(23844.89,33785.87) |  | 53.04(45.23,59.25) | -25.12(-27.32,-22.92)* |
| Monaco | 6844(5772,8139) | 61756.43(52080.85,73447.87) |  | 9562(8087,11319) | 61115.4(51688.99,72347.92) |  | 39.71(38.6,41.1) | -3.35(-5,-1.7)* |
| Mongolia | 73029(61306,85534) | 42144.49(35378.95,49361.24) |  | 149741(125532,176689) | 37927.82(31795.85,44753.47) |  | 105.04(96.29,112.27) | -34.18(-37.93,-30.43)* |
| Montenegro | 47021(39634,54710) | 43161.31(36380.66,50219.57) |  | 77145(64777,90407) | 44241.06(37148.69,51846.85) |  | 64.07(61.25,66.71) | 8.36(6.27,10.46)* |
| Morocco | 659967(560923,778199) | 28145.5(23921.57,33187.69) |  | 1629309(1379758,1925168) | 27119.06(22965.41,32043.5) |  | 146.88(144.14,149.39) | -11.76(-12.59,-10.92)* |
| Mozambique | 550529(458538,651270) | 56338.12(46924.24,66647.39) |  | 996666(830866,1185378) | 55706.6(46439.56,66254.29) |  | 81.04(80.08,81.95) | -3.56(-4.69,-2.44)* |
| Myanmar | 1524127(1275937,1832430) | 38935.39(32595.13,46811.31) |  | 3352733(2815395,4001320) | 39714.33(33349.38,47397.08) |  | 119.98(117.4,122.82) | 6.37(5.19,7.56)* |
| Namibia | 56475(47235,67654) | 52046.11(43530.32,62348.39) |  | 119723(100633,142165) | 52987.25(44538.26,62919.72) |  | 111.99(109.35,114.77) | 5.9(5.01,6.79)* |
| Nauru | 208(175,249) | 28802.92(24177.72,34474.93) |  | 269(226,319) | 29102.71(24478.31,34509.75) |  | 29.21(26.74,31.71) | 3.08(1.51,4.65)* |
| Nepal | 491294(414661,584333) | 31916.3(26937.92,37960.45) |  | 1326025(1124097,1568648) | 33503.61(28401.67,39633.79) |  | 169.9(164.1,176.93) | 15.82(15.53,16.12)* |
| Netherlands | 1881096(1580469,2241778) | 56814.06(47734.34,67707.61) |  | 3411747(2877265,4058178) | 58311.76(49176.69,69360.22) |  | 81.37(79.79,83.33) | 8.5(5.71,11.3)* |
| New Zealand | 407371(344192,475389) | 61912.99(52310.95,72250.46) |  | 900093(767109,1052544) | 63101.4(53778.52,73789.07) |  | 120.95(118.58,124.29) | 5.88(3.43,8.33)* |
| Nicaragua | 102265(87460,119421) | 41156.96(35198.58,48061.68) |  | 340260(291439,398419) | 41799.24(35801.79,48943.76) |  | 232.72(230.76,234.75) | 5.27(3.59,6.95)* |
| Niger | 265067(222693,323697) | 60311.28(50669.76,73651.44) |  | 836225(704510,1014968) | 60064.39(50603.51,72903.12) |  | 215.48(213.21,217.73) | -0.76(-8.62,7.11) |
| Nigeria | 4853910(4070444,5759588) | 66628.57(55874.1,79060.61) |  | 9646896(8139890,11532420) | 64306.15(54260.46,76875.05) |  | 98.74(95.19,102.38) | -11.34(-15.09,-7.58)* |
| Niue | 126(108,147) | 34684.48(29775.49,40436.54) |  | 123(105,144) | 31556.67(26831.78,36833.65) |  | -2.25(-6.02,1.11) | -30.97(-34.73,-27.21)* |
| North Macedonia | 136920(115106,159668) | 41621.18(34990.17,48536.37) |  | 251404(210539,297331) | 42402.02(35509.72,50148.05) |  | 83.61(78.32,88.84) | 6.74(1.17,12.31)* |
| Northern Mariana Islands | 692(583,823) | 28357.21(23871.05,33708.88) |  | 2774(2331,3289) | 28540.65(23979.14,33840.24) |  | 300.73(295.72,305.98) | 1.6(-2.82,6.03) |
| Norway | 676600(568492,802463) | 62479.02(52496.05,74101.57) |  | 993042(837951,1166923) | 61233.32(51670.02,71955.21) |  | 46.77(44.55,49.09) | -6.8(-8.17,-5.43)* |
| Oman | 26970(22771,31959) | 26833.08(22655.65,31797.48) |  | 76459(63878,90498) | 24478.22(20450.46,28972.84) |  | 183.5(175.71,190.65) | -30.22(-36.71,-23.71)* |
| Pakistan | 3152074(2669673,3763917) | 34564.22(29274.43,41273.41) |  | 6537445(5523747,7792215) | 32974.1(27861.13,39303.02) |  | 107.4(102.03,111.55) | -15.09(-16.21,-13.97)* |
| Palau | 487(411,575) | 30410.63(25689.88,35920.7) |  | 1199(1006,1423) | 28643.01(24031.64,33980.16) |  | 146.46(137.02,154.14) | -19.29(-23.07,-15.51)* |
| Palestine | 39808(33802,46969) | 27426.66(23288.44,32360.36) |  | 108893(91408,128516) | 25308.56(21244.87,29869.36) |  | 173.55(167.21,179.41) | -25.26(-28.05,-22.48)* |
| Panama | 104477(89298,122013) | 43120.41(36855.7,50358.07) |  | 333615(286871,389955) | 44866.94(38580.45,52443.99) |  | 219.32(215.24,223.77) | 12.55(10.97,14.13)* |
| Papua New Guinea | 83705(70059,100201) | 28290.76(23678.63,33866.06) |  | 236563(198932,280392) | 28803.34(24221.53,34139.87) |  | 182.61(179.44,186.12) | 5.79(2.8,8.78)* |
| Paraguay | 208181(175694,248051) | 57386.26(48431,68376.64) |  | 562351(475398,672164) | 56797.06(48014.94,67888.19) |  | 170.13(168.19,171.79) | -3.61(-4.6,-2.63)* |
| Peru | 1263549(1070975,1504077) | 64083.73(54316.91,76282.59) |  | 3696661(3134709,4378129) | 66089.19(56042.56,78272.52) |  | 192.56(189,196.23) | 9.98(9.13,10.83)* |
| Philippines | 1978203(1683634,2355925) | 41436.82(35266.57,49348.86) |  | 5774069(4895141,6894985) | 41405.8(35103.02,49443.88) |  | 191.88(188.98,194.71) | -0.21(-0.87,0.45) |
| Poland | 3446563(2903016,4021317) | 44725.83(37672.26,52184.37) |  | 5991351(5096459,6955860) | 49452.55(42066.12,57413.6) |  | 73.84(70.21,78.07) | 32.75(31.05,34.45)* |
| Portugal | 1311434(1098239,1561474) | 53792.08(45047.31,64048.19) |  | 2410615(2042818,2856010) | 61959.3(52505.92,73407.15) |  | 83.82(76.69,91.76) | 45.77(44.33,47.22)* |
| Puerto Rico | 362731(305823,431180) | 59781.07(50402.11,71061.99) |  | 777123(659464,908978) | 66570.78(56491.73,77865.81) |  | 114.24(107.78,121.4) | 34.61(33.17,36.04)* |
| Qatar | 3636(3012,4312) | 22256.86(18440.47,26396.18) |  | 32128(26290,38578) | 20973.6(17162.4,25184.02) |  | 783.68(761.49,802.22) | -18.39(-23.83,-12.95)* |
| Republic of Korea | 2030011(1722090,2384028) | 40789.88(34602.69,47903.3) |  | 7800856(6636751,9078678) | 46645.35(39684.57,54286.11) |  | 284.28(271.81,299.57) | 43.44(39.13,47.76)* |
| Republic of Moldova | 317359(265844,372436) | 41107.89(34435.16,48242.11) |  | 481935(409048,560054) | 45645(38741.74,53043.78) |  | 51.86(47.74,56.39) | 33.68(29.59,37.77)* |
| Romania | 2104339(1762401,2467396) | 42054.41(35220.91,49309.97) |  | 3033779(2561038,3536628) | 50517.94(42645.94,58891.29) |  | 44.17(38.19,51.23) | 59.46(56.13,62.8)* |
| Russian Federation | 13704818(11543298,16075377) | 43420.89(36572.56,50931.51) |  | 19773965(16709423,22873963) | 46521.26(39311.46,53814.48) |  | 44.28(41.79,47.29) | 21.27(15.04,27.51)* |
| Rwanda | 260815(217086,310529) | 55735.74(46390.97,66359.68) |  | 590941(492913,703743) | 55882.73(46612.65,66549.94) |  | 126.58(125.13,128.29) | 0.96(-3.57,5.49) |
| Saint Kitts and Nevis | 3948(3289,4725) | 61735.51(51436.73,73895.61) |  | 6645(5558,8000) | 51597.07(43151.19,62111.73) |  | 68.34(55.39,81.84) | -58.13(-61.31,-54.95)* |
| Saint Lucia | 8573(7155,10205) | 58598.27(48905.16,69755.31) |  | 24276(20543,28868) | 57686.86(48817.01,68599.35) |  | 183.17(176.76,189.92) | -5.04(-5.99,-4.09)* |
| Saint Vincent and the Grenadines | 7103(5946,8444) | 59118.64(49487.9,70281.52) |  | 14505(12215,17307) | 57046.82(48041.09,68069.04) |  | 104.21(99.67,108.18) | -10.82(-12.55,-9.09)* |
| Samoa | 4359(3674,5133) | 30243.97(25495.26,35619.53) |  | 7539(6391,8841) | 30618.7(25957.6,35906.27) |  | 72.96(71.46,74.47) | 4.02(3.13,4.9)* |
| San Marino | 3356(2823,3991) | 57146.67(48070.14,67959.98) |  | 7326(6254,8594) | 61940.43(52879.53,72661.44) |  | 118.27(112.33,125.65) | 25.99(24.4,27.58)* |
| Sao Tome and Principe | 6667(5665,7865) | 59541.41(50591.29,70234.43) |  | 10403(8805,12313) | 56897.04(48157.74,67343.72) |  | 56.03(53.25,58.61) | -14.57(-18.13,-11.01)* |
| Saudi Arabia | 240707(203799,284351) | 27018.28(22875.55,31917.15) |  | 691732(575158,818802) | 22975.76(19103.78,27196.37) |  | 187.38(172.5,201.71) | -52.37(-56.84,-47.9)* |
| Senegal | 307930(259800,364973) | 58272.36(49164.28,69067.16) |  | 747041(634307,883965) | 57857.88(49126.7,68462.59) |  | 142.6(140.83,144.52) | -1.91(-4.57,0.75) |
| Serbia | 831807(697955,980320) | 39995.34(33559.37,47136.19) |  | 1348683(1135225,1572995) | 48077.63(40468.31,56073.87) |  | 62.14(53.14,73.41) | 60.13(56.51,63.75)* |
| Seychelles | 4096(3455,4838) | 42703.91(36016.6,50435.71) |  | 8325(7014,9906) | 40134.42(33813.34,47754.06) |  | 103.24(95.48,109.95) | -19.83(-21.93,-17.72)* |
| Sierra Leone | 200234(169139,237138) | 60410.39(51029.03,71544.34) |  | 351230(297442,415906) | 58142.63(49238.6,68849.08) |  | 75.41(72.33,78.33) | -11.84(-13.95,-9.73)* |
| Singapore | 155811(132646,182421) | 42974.05(36584.93,50313.55) |  | 694338(592672,811541) | 45715.38(39021.7,53432.07) |  | 345.63(338.87,354.03) | 20.54(13.11,27.98)* |
| Slovakia | 463950(389892,541678) | 44720.04(37581.61,52212.18) |  | 764373(644893,890111) | 46600.24(39316.09,54265.9) |  | 64.75(63.24,66.53) | 13.49(10.43,16.56)* |
| Slovenia | 192787(162847,224008) | 44760.53(37809.23,52009.37) |  | 371668(315275,431186) | 50761.17(43059.29,58890.04) |  | 92.79(87.59,98.46) | 41.54(38.36,44.72)* |
| Solomon Islands | 6468(5419,7726) | 28606.67(23969.08,34170.46) |  | 15998(13498,18860) | 29491.55(24883.3,34768.15) |  | 147.35(142.67,152.6) | 9.87(9.23,10.51)* |
| Somalia | 192633(160793,229783) | 53498.84(44656.08,63816.31) |  | 503968(418713,605777) | 53439.66(44399.36,64235.19) |  | 161.62(157.27,165.07) | -0.03(-1.3,1.24) |
| South Africa | 1952760(1656483,2289838) | 57851.43(49074.11,67837.54) |  | 4451017(3762249,5225352) | 56423.75(47692.51,66239.69) |  | 127.93(126.05,129.8) | -8.04(-10.33,-5.75)* |
| South Sudan | 248537(208142,291326) | 59931.5(50190.91,70249.67) |  | 344816(287223,408919) | 55372.31(46123.75,65666.33) |  | 38.74(33.87,43.37) | -25.44(-27.64,-23.24)* |
| Spain | 4425114(3767488,5190835) | 46284.9(39406.4,54294.03) |  | 8190413(7073604,9510114) | 52926.47(45709.65,61454.38) |  | 85.09(78.74,92.12) | 43.21(38.75,47.67)* |
| Sri Lanka | 726498(616133,866543) | 40819.61(34618.53,48688.29) |  | 1999343(1692991,2375864) | 41511.97(35151.25,49329.6) |  | 175.2(171.7,179.02) | 5.46(4.5,6.42)* |
| Sudan | 407666(342119,482343) | 27414.55(23006.67,32436.4) |  | 837583(710166,987904) | 26898.74(22806.8,31726.27) |  | 105.46(101.36,109.79) | -6.06(-7.53,-4.58)* |
| Suriname | 24191(20461,28933) | 55590.58(47017.15,66486.36) |  | 62741(53101,75047) | 56167.67(47537.9,67184.66) |  | 159.35(157.05,162.08) | 3.29(1.82,4.76)* |
| Sweden | 1493225(1256067,1772397) | 62897.17(52907.64,74656.34) |  | 2155102(1812795,2547251) | 64016.63(53848.52,75665.32) |  | 44.33(42.82,46.15) | 5.7(4.48,6.91)* |
| Switzerland | 992210(836492,1177261) | 58739.66(49521.01,69694.78) |  | 1760363(1490518,2078386) | 60063.53(50856.44,70914.48) |  | 77.42(75.32,80.06) | 7.31(6.57,8.05)* |
| Syrian Arab Republic | 234921(198812,276419) | 26644.89(22549.36,31351.64) |  | 604496(507836,713747) | 25584.93(21493.87,30208.9) |  | 157.32(153.04,160.73) | -12.99(-14.98,-11)* |
| Taiwan (Province of China) | 577407(486536,678877) | 20817.79(17541.52,24476.17) |  | 1729436(1497127,2020799) | 22967.48(19882.34,26836.88) |  | 199.52(188.53,212.67) | 31.62(30.02,33.22)* |
| Tajikistan | 197483(166822,230122) | 42168.65(35621.66,49138.06) |  | 385816(323940,456428) | 37562(31537.86,44436.58) |  | 95.37(86.74,102.7) | -37.99(-44.34,-31.63)* |
| Thailand | 2346436(1977739,2797707) | 39540.35(33327.35,47144.82) |  | 8150903(6873679,9646895) | 41932.86(35362.09,49629.09) |  | 247.37(238.74,258.29) | 19(18.63,19.36)* |
| Timor-Leste | 16660(13965,20194) | 38331.83(32132.51,46464.47) |  | 57620(48197,69437) | 40568.26(33934.01,48888.86) |  | 245.86(229.21,266.06) | 18.14(11.8,24.49)* |
| Togo | 110635(93362,131274) | 56714.24(47859.41,67294.27) |  | 342826(287847,407560) | 54879.62(46078.57,65242.19) |  | 209.87(205.12,214.43) | -10.68(-13.7,-7.66)* |
| Tokelau | 74(63,87) | 31408.16(26613.93,36929.34) |  | 83(71,98) | 33029.51(28223.43,38676.96) |  | 12.31(9.58,15.21) | 16.55(11.19,21.92)* |
| Tonga | 2879(2436,3380) | 30339.82(25666.65,35618.39) |  | 4336(3703,5057) | 32283.91(27574.95,37653.54) |  | 50.6(47,54.84) | 20.17(19.08,21.26)* |
| Trinidad and Tobago | 79866(66746,95045) | 58169.33(48613.36,69224.66) |  | 200205(168538,238682) | 57474.15(48383.24,68520.09) |  | 150.67(146.58,154.96) | -3.94(-5.84,-2.03)* |
| Tunisia | 228848(192603,270755) | 26282.54(22120,31095.45) |  | 654903(556641,772116) | 28021.62(23817.26,33036.88) |  | 186.17(180.51,192.6) | 21.07(18.79,23.34)* |
| Turkey | 1587471(1343878,1865798) | 26570.67(22493.48,31229.24) |  | 4674644(3980895,5497368) | 28294.73(24095.6,33274.52) |  | 194.47(188.39,201.17) | 20.46(17.94,22.98)* |
| Turkmenistan | 131449(110735,153931) | 40474.52(34096.42,47396.82) |  | 279953(236474,329444) | 39558.08(33414.34,46551.27) |  | 112.97(108.38,116.91) | -7.4(-9.11,-5.69)* |
| Tuvalu | 337(282,403) | 28982.78(24299.42,34673.55) |  | 551(465,650) | 30259.49(25535.74,35679.04) |  | 63.75(60.52,67.94) | 13.97(12.71,15.22)* |
| Uganda | 606257(504763,712835) | 57964.24(48260.36,68154.13) |  | 1345978(1125418,1588790) | 56756.57(47456.1,66995.38) |  | 122.01(119.08,124.69) | -6.96(-10.24,-3.69)* |
| Ukraine | 5571262(4701877,6529440) | 44688.12(37714.62,52373.84) |  | 6455202(5486130,7498524) | 47541.38(40404.34,55225.25) |  | 15.87(14.03,17.87) | 19.41(14.95,23.88)* |
| United Arab Emirates | 13892(11638,16410) | 24750.86(20735.14,29238.74) |  | 140339(112703,170842) | 19543.64(15695.13,23791.57) |  | 910.24(811.04,1002.8) | -77.76(-82.37,-73.15)* |
| United Kingdom | 8908180(7502614,10511558) | 59975.46(50512.31,70770.41) |  | 12895688(10886791,15181932) | 61319.89(51767.45,72191.14) |  | 44.76(42.96,47.04) | 7.54(4.26,10.81)* |
| United Republic of Tanzania | 1029824(859088,1233333) | 57080.17(47616.73,68360.06) |  | 2382727(1995757,2835595) | 57802.58(48415.08,68788.71) |  | 131.37(129.33,134.06) | 4.13(1.28,6.97)* |
| United States of America | 11671043(10293385,13040475) | 22246.56(19620.56,24856.89) |  | 22413877(19842364,24715643) | 22358.48(19793.32,24654.56) |  | 92.05(86.77,97.99) | 1.39(-0.33,3.11) |
| United States Virgin Islands | 7604(6350,9134) | 54438.61(45466.04,65395.05) |  | 19689(16561,23407) | 61166.73(51448.54,72717.52) |  | 158.95(149.12,170.11) | 37.94(35.09,40.8)* |
| Uruguay | 323913(275276,379232) | 48003.56(40795.62,56201.76) |  | 477677(406085,558719) | 52823.26(44906.39,61785.21) |  | 47.47(43.88,51.55) | 31.01(29.87,32.15)* |
| Uzbekistan | 847378(718324,984872) | 43328.93(36730.03,50359.38) |  | 1792963(1508128,2112716) | 38569.54(32442.26,45447.94) |  | 111.59(103.12,119.11) | -38.04(-42.73,-33.34)* |
| Vanuatu | 2898(2433,3456) | 29072.8(24410.48,34672.97) |  | 8377(7047,9903) | 29214.53(24574.42,34536.18) |  | 189.11(186.3,191.74) | 1.73(0.47,3)* |
| Venezuela (Bolivarian Republic of) | 651162(557173,760407) | 41585(35582.6,48561.65) |  | 2201137(1881224,2576557) | 42069.14(35954.82,49244.35) |  | 238.03(236.12,240.25) | 4.09(2.1,6.08)* |
| Viet Nam | 2826004(2373275,3363830) | 40533.09(34039.64,48247.07) |  | 6928869(5810667,8268677) | 39664.72(33263.51,47334.54) |  | 145.18(141.22,148.48) | -7.08(-8.67,-5.5)* |
| Yemen | 202086(168425,239289) | 25197.09(21000.14,29835.83) |  | 588883(495907,695905) | 26185.08(22050.83,30943.88) |  | 191.4(187.51,195.57) | 12.49(11.03,13.94)* |
| Zambia | 261532(218862,308174) | 56814.61(47544.96,66946.9) |  | 607921(507980,718638) | 56280.74(47028.32,66530.76) |  | 132.45(130.94,134.02) | -2.71(-4.41,-1.01)* |
| Zimbabwe | 353363(295882,417914) | 53311.22(44639.27,63050.04) |  | 570642(477670,685598) | 51535.84(43139.36,61917.79) |  | 61.49(58.86,64.23) | -10.81(-13.12,-8.5)* |

Abbreviation: UI, uncertainty interval; AAPC, average annual percent change; CI, confidence interval.

Note: * indicates statistically significant.
