# Supplementary material for: Long-term spatio-temporal trends in burden of fungal skin diseases in middle-aged and elderly people from 1990 to 2021
Source: PLoS Negl Trop Dis. 2026 Apr 1;20(4):e0014157. doi: 10.1371/journal.pntd.0014157 (PMC13065042; doi:10.1371/journal.pntd.0014157)
Supplement: S7 Table — (DOCX) [file pntd.0014157.s007.docx]

**S7 Table. Prevalent cases and prevalence rate of fungal skin diseases in middle-aged and elderly people across 204 countries and territories in 1990 and 2021, and temporal trends from 1990 to 2021.**

| Characteristic | 1990 | |  | 2021 | |  | 1990-2021 | |
| --- | --- | --- | --- | --- | --- | --- | --- | --- |
|  | Prevent cases, | Prevalence rate per 100000 |  | Prevent cases, | Prevalence rate per 100000 |  | Change of number | AAPC |
|  | No. (95% UI) | No. (95% UI) |  | No. (95% UI) | No. (95% UI) |  | % (95% UI) | (95% CI) |
| Afghanistan | 91846(78453,108798) | 7558.2(6456.04,8953.21) |  | 99824(86054,118765) | 8124.03(7003.36,9665.48) |  | 8.69(4.53,12.75) | 23.9(18.12,29.68)* |
| Albania | 43450(37368,51386) | 12520.81(10768.19,14807.57) |  | 106736(91510,126723) | 13574.57(11638.13,16116.54) |  | 145.65(139.21,151.53) | 26.39(25.11,27.67)* |
| Algeria | 158715(133723,190505) | 7575.77(6382.88,9093.17) |  | 477286(409116,566484) | 7858.67(6736.23,9327.34) |  | 200.72(194.82,207.93) | 12.1(10.93,13.27)* |
| American Samoa | 320(273,378) | 8760.23(7458.1,10326.33) |  | 761(652,890) | 9007.34(7724.01,10542.63) |  | 137.48(134.35,141.08) | 9.58(8.36,10.81)* |
| Andorra | 1727(1481,2034) | 17506.63(15013.59,20611.14) |  | 4995(4340,5812) | 18878.13(16401.18,21965.28) |  | 189.18(180.91,197.71) | 25.37(18.39,32.36)* |
| Angola | 79238(67617,93474) | 12720.55(10855.02,15005.95) |  | 246974(211130,290395) | 12772.57(10918.84,15018.12) |  | 211.69(208.91,214.32) | 1.46(-0.32,3.24) |
| Antigua and Barbuda | 1863(1608,2163) | 21557.73(18613.24,25038.09) |  | 3480(2967,4062) | 18445.4(15727.49,21530.63) |  | 86.83(78.68,95.63) | -50.56(-52.38,-48.75)* |
| Argentina | 778621(668546,905286) | 13992.38(12014.25,16268.63) |  | 1420923(1224759,1641118) | 15224.21(13122.45,17583.45) |  | 82.49(78.8,86.27) | 27.55(26.17,28.92)* |
| Armenia | 59068(50847,69635) | 12134.72(10445.83,14305.65) |  | 106148(91943,125272) | 13494.16(11688.33,15925.32) |  | 79.7(75.69,83.87) | 33.3(26.85,39.76)* |
| Australia | 744497(711023,784890) | 22687.46(21667.37,23918.38) |  | 1828600(1749887,1918818) | 24684.71(23622.14,25902.58) |  | 145.62(143.82,147.36) | 26.97(24.77,29.16)* |
| Austria | 377310(324658,442917) | 19389.88(16684.09,22761.4) |  | 600731(523049,700328) | 20328.12(17699.44,23698.36) |  | 59.21(55.92,62.08) | 15.42(11.77,19.07)* |
| Azerbaijan | 106745(92105,125717) | 12238.58(10560.09,14413.76) |  | 218531(186420,259007) | 11406.76(9730.69,13519.55) |  | 104.72(98.22,111.26) | -23.14(-27.62,-18.66)* |
| Bahamas | 4679(4003,5425) | 19053.64(16300.19,22092.81) |  | 12969(11084,15044) | 18022.65(15402.76,20906.07) |  | 177.19(170.62,184.04) | -18.21(-19.71,-16.7)* |
| Bahrain | 1911(1616,2293) | 6809.75(5758.29,8172.04) |  | 10491(8780,12739) | 6461.12(5407.38,7845.67) |  | 449.09(431.86,465.63) | -16.6(-21.89,-11.31)* |
| Bangladesh | 766287(657542,897758) | 10116.45(8680.82,11852.12) |  | 2362810(2025227,2777692) | 10112.11(8667.36,11887.68) |  | 208.35(204.75,211.7) | 0.17(-1.56,1.9) |
| Barbados | 10293(8853,12076) | 21953.74(18881.51,25756.09) |  | 18149(15595,20966) | 19918.03(17114.84,23009.57) |  | 76.33(69.82,83.66) | -32.01(-34.43,-29.6)* |
| Belarus | 302834(261098,358700) | 13081.77(11278.85,15495.04) |  | 393856(340419,464866) | 13691.71(11834.05,16160.25) |  | 30.06(28.91,31.36) | 14.75(9.74,19.77)* |
| Belgium | 495669(426026,581604) | 18904.79(16248.61,22182.35) |  | 778229(678916,906211) | 20583.87(17957.06,23968.93) |  | 57.01(53.13,60.59) | 27.21(25.16,29.26)* |
| Belize | 3064(2638,3530) | 19906.03(17135.24,22931.63) |  | 9201(7888,10647) | 18370.99(15749.09,21257.72) |  | 200.3(193.24,207.83) | -26.04(-26.67,-25.4)* |
| Benin | 69595(59811,80844) | 21753.63(18695.38,25269.95) |  | 170576(145945,198886) | 20624.35(17646.16,24047.24) |  | 145.1(140.86,149.96) | -15.06(-17.07,-13.05)* |
| Bermuda | 2024(1728,2345) | 19130.87(16336.3,22168.53) |  | 4881(4211,5645) | 20956.94(18080.1,24238.27) |  | 141.19(135.8,146.69) | 29.45(27.32,31.57)* |
| Bhutan | 3697(3126,4409) | 9315.79(7877.86,11109.52) |  | 10362(8935,12144) | 10404.45(8971.45,12194.18) |  | 180.28(169.14,191.87) | 35.68(34.56,36.8)* |
| Bolivia (Plurinational State of) | 130442(110168,152568) | 24877.7(21011.18,29097.57) |  | 389065(328645,454522) | 25174.54(21265.01,29409.95) |  | 198.27(197.01,199.58) | 3.76(1.79,5.73)* |
| Bosnia and Herzegovina | 86718(74435,102415) | 11821.76(10147.29,13961.7) |  | 153156(131963,181299) | 13983.43(12048.43,16552.89) |  | 76.61(69.7,84.19) | 54.74(50.78,58.71)* |
| Botswana | 15771(13436,18681) | 16963.58(14451.94,20093.6) |  | 41572(35612,48972) | 17148.36(14690.06,20201.07) |  | 163.6(160.74,166.56) | 4.6(0.4,8.8)* |
| Brazil | 2734273(2336302,3201098) | 18501.61(15808.71,21660.4) |  | 8495074(7319919,9843263) | 19615.6(16902.1,22728.64) |  | 210.69(205.37,216.43) | 18.86(18.31,19.41)* |
| Brunei Darussalam | 2077(1783,2422) | 13262.93(11389.37,15470.9) |  | 7162(6093,8553) | 11961.7(10175.95,14285.11) |  | 244.9(233.86,255.37) | -33.9(-41.5,-26.3)* |
| Bulgaria | 282075(239978,337149) | 12463.06(10603.06,14896.43) |  | 352950(303141,415051) | 14849.12(12753.59,17461.8) |  | 25.13(19.49,31.05) | 56.99(53.14,60.86)* |
| Burkina Faso | 146761(124839,172333) | 20292.16(17261.14,23828.04) |  | 310182(264882,362447) | 20631.84(17618.72,24108.28) |  | 111.35(109.76,113.11) | 5.63(3.7,7.56)* |
| Burundi | 79226(67277,92255) | 21051.41(17876.41,24513.31) |  | 160203(135503,188428) | 20012.16(16926.75,23537.9) |  | 102.21(95.5,109.22) | -16.55(-18.35,-14.74)* |
| Cabo Verde | 9223(8018,10661) | 23366.92(20313.4,27010.31) |  | 16323(14063,18916) | 21296.96(18348.55,24679.63) |  | 76.99(69.89,85.58) | -29.59(-33.66,-25.51)* |
| Cambodia | 102857(86732,123102) | 13882.28(11706.01,16614.72) |  | 301240(254277,360335) | 13857.68(11697.29,16576.19) |  | 192.87(191.64,194.05) | -0.51(-0.83,-0.2)* |
| Cameroon | 136208(117459,160744) | 18395.5(15863.28,21709.07) |  | 374278(322279,441707) | 18387.71(15833.09,21700.4) |  | 174.78(174.12,175.37) | 0.1(-3.12,3.32) |
| Canada | 349913(298630,416022) | 6409.83(5470.39,7620.82) |  | 833608(718952,986740) | 6793.22(5858.87,8041.12) |  | 138.23(134.56,142.91) | 18.37(15.44,21.3)* |
| Central African Republic | 23668(20182,28053) | 12535.08(10688.75,14857.73) |  | 44083(37525,52219) | 12324.06(10490.56,14598.52) |  | 86.26(84.53,87.96) | -5.5(-7.58,-3.42)* |
| Chad | 99837(85690,116239) | 21544.28(18491.3,25083.64) |  | 187825(160161,220367) | 20228.37(17249.03,23733.1) |  | 88.13(83.49,92.99) | -20.49(-24.98,-16)* |
| Chile | 231681(198926,269667) | 13776.47(11828.76,16035.22) |  | 655626(565881,755564) | 14642.08(12637.81,16874) |  | 182.99(178.58,187.6) | 19.83(18.76,20.89)* |
| China | 9854671(8445123,11721292) | 6866.5(5884.36,8167.12) |  | 27311376(23584469,32102137) | 7207.02(6223.55,8471.22) |  | 177.14(170.83,184.36) | 15.62(13.76,17.48)* |
| Colombia | 340896(292806,405284) | 11840.82(10170.43,14077.3) |  | 1224594(1064151,1428365) | 12804.87(11127.21,14935.59) |  | 259.23(251.94,267.98) | 25.35(24.7,25.99)* |
| Comoros | 6571(5549,7699) | 20246.42(17097.02,23722.79) |  | 16909(14426,19621) | 20919.4(17846.86,24274.66) |  | 157.34(152.88,161.72) | 11.21(6.61,15.82)* |
| Congo | 23462(19963,27560) | 13180.03(11214.62,15481.91) |  | 56385(48455,66162) | 12976.21(11151.15,15226.24) |  | 140.32(137.08,143.97) | -5.41(-7.31,-3.51)* |
| Cook Islands | 193(166,227) | 9053.38(7770.87,10643.71) |  | 449(389,529) | 9519.96(8244.82,11224.3) |  | 132.23(127.48,138.43) | 15.67(12.17,19.17)* |
| Costa Rica | 35676(30772,41845) | 12544.65(10819.98,14713.58) |  | 121388(105413,141779) | 12678.06(11009.61,14807.81) |  | 240.25(236.97,244.08) | 3.22(2.29,4.14)* |
| Croatia | 138202(118968,163215) | 12415.06(10687.23,14662.04) |  | 224319(193478,262636) | 15041.61(12973.54,17610.9) |  | 62.31(54.16,70.49) | 62.67(58.34,67.01)* |
| Cuba | 339821(291408,394690) | 20051.99(17195.27,23289.69) |  | 690310(597362,796925) | 20201.94(17481.81,23322.03) |  | 103.14(100.08,106.27) | 2.6(-0.33,5.54) |
| Cyprus | 24299(20646,28905) | 17394.88(14780.01,20692.23) |  | 65536(56098,77242) | 18751.27(16050.96,22100.69) |  | 169.71(163.06,177.4) | 24.23(23.24,25.23)* |
| Czechia | 324434(278231,385285) | 13701.68(11750.42,16271.56) |  | 529680(455863,620058) | 15162.02(13049.02,17749.09) |  | 63.26(59.04,67.31) | 32.7(30.28,35.12)* |
| CÔTE D'IVOIRE | 121884(103197,144198) | 19016.7(16101.03,22498.23) |  | 351693(299430,413898) | 19612.79(16698.23,23081.77) |  | 188.55(184.83,192.19) | 10.59(9.12,12.05)* |
| Democratic People's Republic of Korea | 164699(139975,194294) | 6188.51(5259.5,7300.51) |  | 369695(317276,435995) | 6559.8(5629.68,7736.22) |  | 124.47(117.96,131.66) | 18.84(16.99,20.69)* |
| Democratic Republic of the Congo | 335399(285911,396089) | 12700.93(10826.93,14999.16) |  | 778295(667693,913730) | 12886.08(11054.85,15128.45) |  | 132.05(129.36,135.34) | 4.77(3.25,6.29)* |
| Denmark | 260478(224826,304863) | 19965.61(17232.89,23367.71) |  | 390100(337911,454907) | 20262.59(17551.77,23628.82) |  | 49.76(48.67,50.85) | 5.17(1.06,9.27)* |
| Djibouti | 4103(3457,4882) | 19125.03(16114.41,22756.21) |  | 20216(17036,23926) | 19493.5(16427.75,23071.34) |  | 392.69(389.43,395.58) | 6.46(5.04,7.88)* |
| Dominica | 2000(1708,2337) | 20367.99(17395.4,23807.48) |  | 2833(2421,3288) | 18782.32(16051.24,21797.56) |  | 41.67(36.89,46.86) | -26.14(-27.21,-25.06)* |
| Dominican Republic | 113472(96750,132582) | 18559.49(15824.46,21685.03) |  | 327501(281806,377962) | 19598.81(16864.25,22618.58) |  | 188.62(184.44,193.3) | 17.56(15.52,19.61)* |
| Ecuador | 222317(189287,258077) | 25855.64(22014.25,30014.56) |  | 731046(623684,846357) | 26415.22(22535.85,30581.8) |  | 228.83(226.04,231.59) | 7.12(4.04,10.2)* |
| Egypt | 323540(274954,390203) | 7235.76(6149.16,8726.64) |  | 776772(658306,936613) | 7019.54(5948.98,8464) |  | 140.09(137.65,142.6) | -9.18(-11.3,-7.06)* |
| El Salvador | 61669(53380,72127) | 12612.34(10917.01,14751.15) |  | 138335(120532,161332) | 13527.99(11787.02,15776.93) |  | 124.32(120.19,129.08) | 22.61(20.73,24.48)* |
| Equatorial Guinea | 4197(3591,4908) | 13147.68(11250.21,15375) |  | 10613(9109,12318) | 13417.22(11516.5,15573.62) |  | 152.87(149.94,155.94) | 6.69(4.65,8.73)* |
| Eritrea | 31625(26566,38159) | 17895.44(15033.07,21592.92) |  | 83608(70547,99170) | 18975.44(16011.18,22507.5) |  | 164.37(157.56,171.81) | 19.02(16.19,21.86)* |
| Estonia | 48068(41290,56993) | 13409.07(11518.22,15898.88) |  | 68723(59544,80364) | 15708.97(13610.78,18369.92) |  | 42.97(37.99,47.95) | 51.24(46.76,55.72)* |
| Eswatini | 7823(6697,9234) | 17325.09(14831.79,20450.01) |  | 15252(12979,18091) | 16845.77(14334.71,19981.81) |  | 94.96(92.58,97.24) | -8.77(-9.55,-7.99)* |
| Ethiopia | 833086(702546,989184) | 25928.76(21865.87,30787.11) |  | 1914493(1634722,2245632) | 27966.86(23879.97,32804.13) |  | 129.81(121.46,138.03) | 23.18(18.15,28.21)* |
| Fiji | 4941(4209,5817) | 8765.85(7467.06,10320.93) |  | 11914(10139,14058) | 8712.65(7414.82,10280.55) |  | 141.13(139.64,142.55) | -1.89(-2.83,-0.94)* |
| Finland | 224395(192413,263553) | 18898.8(16205.19,22196.66) |  | 422870(367369,489484) | 21010.42(18252.85,24320.18) |  | 88.45(83.2,93.07) | 32.92(29.36,36.47)* |
| France | 2696430(2328974,3166426) | 19375.69(16735.27,22752.93) |  | 4688747(4094744,5430709) | 21206.34(18519.77,24562.09) |  | 73.89(69.95,77.48) | 29.29(28.25,30.32)* |
| Gabon | 13815(11759,16293) | 14111.36(12011.3,16642.14) |  | 23643(20093,27874) | 13226.76(11240.55,15593.68) |  | 71.14(67.19,75.07) | -20.37(-25.08,-15.66)* |
| Gambia | 11309(9613,13257) | 20420.75(17358.58,23938.8) |  | 32490(27789,37770) | 21010.28(17970.41,24424.19) |  | 187.31(184.02,190.58) | 9.02(6.79,11.25)* |
| Georgia | 142614(122983,168833) | 12839.45(11072.12,15199.94) |  | 151044(131018,177383) | 14386.94(12479.5,16895.76) |  | 5.91(3.38,8.65) | 36.88(32.74,41.02)* |
| Germany | 4027534(3470484,4731643) | 19194.09(16539.35,22549.68) |  | 6526070(5685921,7561522) | 20708.54(18042.58,23994.24) |  | 62.04(57.94,65.46) | 24.46(19.69,29.24)* |
| Ghana | 107347(91756,126583) | 10511.83(8985.13,12395.5) |  | 289448(247664,342024) | 10613.47(9081.35,12541.35) |  | 169.64(167.86,171.48) | 3.47(2.23,4.7)* |
| Greece | 485231(417652,568746) | 18148.5(15620.92,21272.1) |  | 802974(701110,920069) | 21846.97(19075.5,25032.82) |  | 65.48(58.31,72.55) | 60.42(58.49,62.36)* |
| Greenland | 282(239,335) | 5114.7(4332.58,6088) |  | 699(592,835) | 5151.56(4361.33,6147.08) |  | 148.15(145.73,150.4) | 1.96(-4.35,8.27) |
| Grenada | 2604(2250,3013) | 21589.47(18659.02,24981.05) |  | 3642(3088,4251) | 18130.42(15374.66,21164.9) |  | 39.88(33.56,46.69) | -56.36(-59.46,-53.27)* |
| Guam | 1104(934,1311) | 8562.69(7244.73,10167.96) |  | 3699(3220,4307) | 9816.74(8543.52,11429.39) |  | 235.09(220.99,254.95) | 44.3(41.78,46.82)* |
| Guatemala | 64319(54867,76891) | 11184.19(9540.61,13370.33) |  | 225466(194221,265271) | 12315.34(10608.7,14489.59) |  | 250.54(241.03,261.11) | 31.04(28.7,33.37)* |
| Guinea | 117686(100797,137146) | 21266.18(18214.33,24782.76) |  | 191478(163966,223122) | 20959.62(17948.01,24423.35) |  | 62.7(61.57,63.96) | -4.31(-5.91,-2.71)* |
| Guinea-Bissau | 12761(10818,15040) | 19990.56(16947.84,23562.15) |  | 22152(18774,26281) | 19284.02(16343.89,22879.1) |  | 73.59(70.94,76.41) | -11.65(-14.09,-9.2)* |
| Guyana | 11311(9638,13182) | 18452.16(15722.67,21504.74) |  | 19975(16959,23233) | 17737.37(15058.85,20630.32) |  | 76.6(74.24,79.21) | -12.43(-14.7,-10.15)* |
| Haiti | 93156(78519,109031) | 17271.73(14557.99,20215) |  | 204313(172705,238165) | 17240.34(14573.14,20096.84) |  | 119.32(118.15,120.51) | -0.49(-3.6,2.61) |
| Honduras | 39400(33808,46908) | 11820.5(10142.8,14073.18) |  | 123629(105801,147227) | 11755.48(10060.3,13999.38) |  | 213.78(212.13,215.39) | -2.14(-7.13,2.85) |
| Hungary | 345446(295983,411666) | 13411.67(11491.3,15982.6) |  | 482938(416349,566511) | 15186.13(13092.24,17814.13) |  | 39.8(36.06,43.73) | 40.33(36.18,44.48)* |
| Iceland | 9178(7933,10753) | 19234.44(16625.33,22536.29) |  | 19084(16584,22253) | 19595.97(17029.03,22850.14) |  | 107.94(106.29,109.5) | 5.99(4.22,7.77)* |
| India | 7068565(5988892,8348085) | 9219.57(7811.35,10888.46) |  | 19600046(16749602,22983837) | 9749.07(8331.25,11432.16) |  | 177.28(171,185.11) | 18.34(16.41,20.26)* |
| Indonesia | 2281195(1930932,2728224) | 14120.06(11952.01,16887.06) |  | 5861960(4943448,7037565) | 13997.19(11803.97,16804.3) |  | 156.97(155.87,157.89) | -2.65(-3.87,-1.42)* |
| Iran (Islamic Republic of) | 339901(286568,408807) | 7373.24(6216.32,8867.95) |  | 1089840(936078,1274002) | 8389.23(7205.62,9806.85) |  | 220.63(206.73,235.35) | 42.36(40.19,44.54)* |
| Iraq | 107078(92951,125703) | 8482.89(7363.71,9958.39) |  | 290149(248132,343543) | 7468.08(6386.61,8842.38) |  | 170.97(161.92,179.69) | -40.81(-42.75,-38.87)* |
| Ireland | 127894(109364,150851) | 18787.98(16065.88,22160.39) |  | 253891(219532,297651) | 19345.98(16727.96,22680.46) |  | 98.52(95.53,101.71) | 9.32(7.94,10.7)* |
| Israel | 225389(201090,252760) | 27807.14(24809.22,31183.94) |  | 580448(522629,647625) | 29043.54(26150.5,32404.83) |  | 157.53(154.67,160.39) | 14.11(12,16.21)* |
| Italy | 2883660(2462472,3368651) | 18947.65(16180.16,22134.38) |  | 4913549(4246664,5615705) | 21629.59(18693.94,24720.5) |  | 70.39(64.53,76.5) | 42.28(37.17,47.4)* |
| Jamaica | 61483(53011,71310) | 20875.29(17998.55,24211.8) |  | 107321(92906,123409) | 20291.68(17566.13,23333.59) |  | 74.55(70.5,79.45) | -9.02(-12.03,-6)* |
| Japan | 4254538(3661653,5013036) | 14367.01(12364.91,16928.35) |  | 10288720(8940185,11977928) | 19709.85(17126.49,22945.82) |  | 141.83(125.55,157.46) | 102.94(97.68,108.21)* |
| Jordan | 15934(13640,18774) | 7410.75(6344.03,8731.66) |  | 92711(79328,109567) | 7374.13(6309.71,8714.89) |  | 481.84(475.25,487.57) | -1.38(-5.18,2.43) |
| Kazakhstan | 265196(228352,314410) | 12680.59(10918.85,15033.78) |  | 382471(327657,452428) | 12052.23(10324.95,14256.68) |  | 44.22(41.97,46.51) | -16.81(-21.69,-11.93)* |
| Kenya | 225028(193818,266597) | 16884.2(14542.47,20003.22) |  | 605412(514989,727050) | 16184.16(13766.92,19435.81) |  | 169.04(153.88,188.82) | -13.28(-14.17,-12.38)* |
| Kiribati | 525(447,618) | 8806.15(7505.76,10364.6) |  | 1076(912,1281) | 8554.28(7250.71,10186.16) |  | 105.06(101.03,108.44) | -9.39(-10.44,-8.34)* |
| Kuwait | 6724(5749,7921) | 7353.96(6287.84,8662.23) |  | 34910(30099,41186) | 7487.79(6456.02,8833.9) |  | 419.16(411.2,428.32) | 9.97(-14.94,34.95) |
| Kyrgyzstan | 65493(56564,77343) | 12719.21(10985.1,15020.45) |  | 100023(85881,118027) | 11826.54(10154.42,13955.3) |  | 52.72(48.53,56.93) | -23.72(-29.89,-17.54)* |
| Lao People's Democratic Republic | 47662(40106,57264) | 13898.17(11695.06,16698.1) |  | 108802(91915,129774) | 14072.57(11888.36,16785.11) |  | 128.28(125.99,130.99) | 3.95(2.76,5.15)* |
| Latvia | 84537(72809,100030) | 13520.73(11645.03,15998.78) |  | 101492(87696,118774) | 15423.85(13327.32,18050.19) |  | 20.06(16.26,23.71) | 41.95(39.89,44.01)* |
| Lebanon | 29248(25138,34443) | 7714.44(6630.44,9084.66) |  | 93037(80948,109121) | 9493.29(8259.75,11134.51) |  | 218.1(200.76,236.45) | 67.94(64.3,71.58)* |
| Lesotho | 25467(21783,29863) | 18211.27(15577.04,21354.93) |  | 31051(26414,36734) | 17197.5(14629.26,20344.96) |  | 21.93(19.29,24.46) | -17.63(-21.45,-13.81)* |
| Liberia | 40034(34189,46781) | 21244.96(18143.42,24825.39) |  | 66083(56530,77244) | 20017.66(17124.05,23398.55) |  | 65.07(60.44,69.98) | -20.23(-24.74,-15.72)* |
| Libya | 25377(21987,29755) | 8158.89(7068.97,9566.65) |  | 65372(56519,76600) | 7816.69(6758.13,9159.2) |  | 157.61(155.58,159.46) | -13.72(-17.38,-10.06)* |
| Lithuania | 107098(92507,126581) | 13514.84(11673.58,15973.35) |  | 147903(128091,173100) | 15282.72(13235.5,17886.28) |  | 38.1(33.92,42.27) | 39.86(35.81,43.91)* |
| Luxembourg | 17279(14819,20273) | 18503.1(15868.89,21708.97) |  | 34304(29796,39971) | 19304.2(16767.13,22493.1) |  | 98.53(94.8,102.38) | 13.67(10.93,16.42)* |
| Madagascar | 172746(146532,201626) | 20445.58(17342.89,23863.6) |  | 349624(294863,415466) | 18932.56(15967.2,22497.98) |  | 102.39(97.03,107.68) | -24.78(-26.54,-23.02)* |
| Malawi | 126394(106444,148057) | 20095.15(16923.29,23539.29) |  | 237540(200604,276905) | 20317.71(17158.45,23684.77) |  | 87.94(86.2,89.5) | 3.98(1.63,6.33)* |
| Malaysia | 215468(184636,253781) | 14599.22(12510.18,17195.18) |  | 699230(595047,830836) | 14261.88(12136.9,16946.18) |  | 224.52(220.49,228.02) | -7.82(-8.83,-6.8)* |
| Maldives | 2110(1763,2538) | 13766.33(11498.41,16555.93) |  | 7855(6672,9293) | 14441.12(12265.16,17083.23) |  | 272.23(261.92,284.55) | 15.61(14.39,16.83)* |
| Mali | 174690(147843,206869) | 26125.47(22110.45,30937.94) |  | 382985(324928,451394) | 26460.68(22449.49,31187.14) |  | 119.24(117.67,120.88) | 4.11(2.95,5.27)* |
| Malta | 12995(11159,15271) | 17938(15403.56,21078.82) |  | 32314(27947,37771) | 20477.12(17710.09,23935.75) |  | 148.65(140.66,156.32) | 42.7(39.82,45.59)* |
| Marshall Islands | 229(195,272) | 8991.53(7665.33,10660.18) |  | 495(417,592) | 8443.05(7117.18,10104.25) |  | 115.71(106.89,123.65) | -20.71(-23.06,-18.36)* |
| Mauritania | 35151(30143,40940) | 21173.89(18157.1,24660.96) |  | 75167(64355,87515) | 20951.98(17938.16,24393.8) |  | 113.84(110.93,116.73) | -3.04(-5.63,-0.44)* |
| Mauritius | 17036(14427,20369) | 14005.32(11860.48,16745.93) |  | 48715(41640,57614) | 14398.52(12307.32,17028.92) |  | 185.96(182.57,189.8) | 8.91(7.95,9.87)* |
| Mexico | 785186(677686,921818) | 11293.19(9747.04,13258.34) |  | 2880852(2487890,3385401) | 13367.95(11544.49,15709.19) |  | 266.9(243.04,291.68) | 54.57(47.86,61.29)* |
| Micronesia (Federated States of) | 729(624,864) | 9153.83(7843.94,10851.12) |  | 1121(949,1341) | 8520.23(7209.16,10192.2) |  | 53.85(46.78,60.05) | -23.45(-24.82,-22.08)* |
| Monaco | 2315(2001,2684) | 20890.21(18056.76,24217.62) |  | 3263(2840,3776) | 20855.79(18150.54,24136.46) |  | 40.95(39.62,42.22) | -0.56(-2.35,1.24) |
| Mongolia | 21885(18784,26007) | 12629.92(10840.25,15008.37) |  | 44799(38333,53095) | 11347.1(9709.37,13448.43) |  | 104.7(96.99,112.29) | -34.71(-38.61,-30.8)* |
| Montenegro | 14145(12256,16693) | 12983.94(11249.84,15322.87) |  | 23116(19736,27543) | 13256.88(11318.48,15795.61) |  | 63.43(60.96,66.34) | 7.44(5.37,9.5)* |
| Morocco | 192385(165708,227495) | 8204.62(7066.93,9701.94) |  | 474336(407856,560795) | 7895.09(6788.56,9334.16) |  | 146.55(143.87,149.29) | -12.21(-13.08,-11.34)* |
| Mozambique | 196538(166030,230206) | 20112.67(16990.64,23558.04) |  | 353210(297461,414826) | 19741.98(16625.99,23185.85) |  | 79.72(78.68,80.74) | -5.91(-7.2,-4.62)* |
| Myanmar | 545032(458760,653310) | 13923.41(11719.49,16689.49) |  | 1192786(1012288,1419593) | 14128.98(11990.92,16815.6) |  | 118.85(116.55,121.49) | 4.76(3.75,5.78)* |
| Namibia | 18483(15745,21887) | 17033.42(14510,20170.54) |  | 39289(33616,46338) | 17388.46(14878.03,20508.4) |  | 112.57(109.15,115.87) | 6.74(4.94,8.54)* |
| Nauru | 62(53,74) | 8628.38(7271.38,10234.93) |  | 80(68,95) | 8697.49(7393.29,10244.22) |  | 28.91(26.45,31.3) | 2.36(0.57,4.16)* |
| Nepal | 145836(123547,173237) | 9474.09(8026.09,11254.1) |  | 390880(334167,458814) | 9876.05(8443.14,11592.5) |  | 168.03(162.45,174.93) | 13.52(13.26,13.78)* |
| Netherlands | 632258(543694,742911) | 19095.86(16421,22437.86) |  | 1157690(1002175,1357100) | 19786.62(17128.64,23194.84) |  | 83.1(81.01,84.9) | 11.58(8.4,14.77)* |
| New Zealand | 150678(129700,175150) | 22900.33(19712.03,26619.52) |  | 338075(292622,389986) | 23700.88(20514.39,27340.14) |  | 124.37(121.9,127.34) | 10.76(7.74,13.78)* |
| Nicaragua | 29603(25444,35156) | 11913.81(10239.94,14148.88) |  | 98453(84804,116071) | 12094.41(10417.71,14258.73) |  | 232.58(230.71,234.61) | 5.14(3.55,6.74)* |
| Niger | 108577(90911,125876) | 24704.71(20685.16,28640.87) |  | 342296(288484,395416) | 24586.4(20721.22,28401.97) |  | 215.26(212.42,218.01) | -0.41(-3.89,3.06) |
| Nigeria | 2022028(1736549,2339749) | 27755.94(23837.23,32117.22) |  | 4009952(3438049,4662381) | 26730.32(22918.01,31079.4) |  | 98.31(95.03,101.55) | -11.92(-15.78,-8.05)* |
| Niue | 37(33,44) | 10294.03(8965.36,12032.98) |  | 37(32,43) | 9394.07(8131.29,11063.83) |  | -1.95(-5.78,1.62) | -29.87(-33.4,-26.34)* |
| North Macedonia | 41195(35494,48601) | 12522.66(10789.46,14773.98) |  | 75170(63985,89820) | 12678.3(10791.71,15149.1) |  | 82.47(77.69,87.37) | 4.27(-2.62,11.16) |
| Northern Mariana Islands | 207(176,246) | 8498.96(7199.43,10088.36) |  | 829(706,984) | 8531.02(7262.27,10123.8) |  | 299.66(294.65,304.23) | 0.97(-2.83,4.76) |
| Norway | 224274(191667,259974) | 20710.02(17699.06,24006.7) |  | 331492(285163,382554) | 20440.56(17583.84,23589.17) |  | 47.81(45.93,49.96) | -4.56(-6.2,-2.93)* |
| Oman | 7850(6703,9302) | 7810.6(6669.26,9255.19) |  | 22230(18935,26335) | 7116.95(6062.13,8431.1) |  | 183.17(175.75,190.18) | -30.57(-37.47,-23.66)* |
| Pakistan | 947969(811375,1125750) | 10394.99(8897.17,12344.46) |  | 1969391(1676397,2343796) | 9933.38(8455.55,11821.83) |  | 107.75(103.53,112.12) | -14.58(-15.58,-13.57)* |
| Palau | 145(124,172) | 9048.58(7735.36,10754.45) |  | 359(305,425) | 8565.19(7282.88,10153.89) |  | 147.7(139.35,155.78) | -17.59(-20.98,-14.2)* |
| Palestine | 11588(9973,13707) | 7983.96(6870.97,9443.48) |  | 31675(27100,37449) | 7361.85(6298.51,8703.91) |  | 173.34(167.4,178.99) | -25.49(-28.26,-22.72)* |
| Panama | 30304(26104,35677) | 12507.32(10773.68,14724.84) |  | 96967(84274,113146) | 13040.86(11333.82,15216.76) |  | 219.98(216.01,224.48) | 13.33(11.76,14.9)* |
| Papua New Guinea | 25049(21096,29976) | 8465.91(7130.18,10131.42) |  | 70911(60376,83938) | 8633.96(7351.19,10220.12) |  | 183.09(179.56,187.32) | 6.33(4.97,7.7)* |
| Paraguay | 69594(59686,80755) | 19184.11(16452.85,22260.5) |  | 187881(161346,217693) | 18975.82(16295.89,21986.88) |  | 169.96(168.25,171.89) | -3.68(-4.46,-2.91)* |
| Peru | 507924(439926,591226) | 25760.52(22311.84,29985.34) |  | 1492554(1299994,1728789) | 26683.99(23241.4,30907.41) |  | 193.85(190.67,197.19) | 11.46(10.28,12.64)* |
| Philippines | 689191(585971,819741) | 14436.29(12274.17,17170.88) |  | 2013422(1710177,2392936) | 14438.23(12263.67,17159.73) |  | 192.14(189.3,194.96) | 0.07(-0.27,0.41) |
| Poland | 1039610(892695,1242108) | 13490.95(11584.45,16118.75) |  | 1817242(1564892,2147448) | 14999.5(12916.6,17725.02) |  | 74.8(71.38,78.79) | 34.58(31.78,37.37)* |
| Portugal | 438284(375364,515862) | 17977.42(15396.57,21159.5) |  | 820069(714213,946095) | 21077.97(18357.2,24317.18) |  | 87.11(79.73,94.67) | 51.53(49.88,53.19)* |
| Puerto Rico | 121517(103949,141277) | 20026.99(17131.66,23283.57) |  | 264110(228725,305197) | 22624.43(19593.32,26144.14) |  | 117.34(110.77,124.13) | 39.3(38.13,40.47)* |
| Qatar | 1058(894,1272) | 6478.17(5472.97,7785.43) |  | 9342(7772,11319) | 6098.76(5073.47,7389.24) |  | 782.83(760.42,802.46) | -18.84(-23.93,-13.75)* |
| Republic of Korea | 612963(522562,719580) | 12316.53(10500.07,14458.83) |  | 2389560(2058738,2763542) | 14288.41(12310.26,16524.64) |  | 289.84(277.19,304.26) | 47.71(43,52.41)* |
| Republic of Moldova | 94959(81200,112651) | 12300.17(10517.91,14591.87) |  | 145233(125331,171670) | 13755.27(11870.33,16259.25) |  | 52.94(49.04,57.36) | 36.09(30.91,41.26)* |
| Romania | 630057(539260,750300) | 12591.45(10776.91,14994.45) |  | 915406(788058,1073672) | 15243.18(13122.59,17878.58) |  | 45.29(38.99,51.67) | 61.99(59.05,64.92)* |
| Russian Federation | 4127579(3551237,4943990) | 13077.38(11251.36,15664.01) |  | 5978533(5161150,7088104) | 14065.41(12142.39,16675.84) |  | 44.84(42.38,47.84) | 22.55(16.14,28.97)* |
| Rwanda | 93400(78767,109897) | 19959.37(16832.38,23484.94) |  | 209908(177157,246546) | 19850.08(16752.99,23314.82) |  | 124.74(123.06,126.51) | -2.21(-14.16,9.76) |
| Saint Kitts and Nevis | 1322(1120,1553) | 20680.97(17510.32,24281.12) |  | 2191(1847,2562) | 17009.56(14342.6,19894.63) |  | 65.66(53.39,79.74) | -63.29(-67.68,-58.89)* |
| Saint Lucia | 2859(2427,3329) | 19541.3(16591.71,22758.03) |  | 8124(6991,9379) | 19305.78(16611.93,22286.55) |  | 184.18(178.72,190.24) | -3.87(-4.76,-2.98)* |
| Saint Vincent and the Grenadines | 2372(2023,2760) | 19742.31(16836.21,22973.44) |  | 4849(4152,5642) | 19070.37(16331.38,22191.5) |  | 104.42(100.49,108.73) | -10.49(-12.33,-8.64)* |
| Samoa | 1301(1114,1528) | 9026.08(7729.52,10600.91) |  | 2248(1935,2640) | 9129.97(7857.89,10722.81) |  | 72.81(71.16,74.53) | 3.66(2.76,4.56)* |
| San Marino | 1134(977,1333) | 19303.65(16634.47,22688.84) |  | 2515(2202,2899) | 21267.85(18620.3,24513.19) |  | 121.86(114.86,128.56) | 31.3(29.82,32.78)* |
| Sao Tome and Principe | 2384(2044,2766) | 21290.36(18250.62,24701.17) |  | 3707(3175,4323) | 20274.16(17362.94,23641.74) |  | 55.49(52.88,58.13) | -15.58(-19.34,-11.82)* |
| Saudi Arabia | 70184(60089,83120) | 7877.82(6744.68,9329.81) |  | 201264(170087,242576) | 6684.97(5649.4,8057.13) |  | 186.77(172.56,201.37) | -52.93(-57.36,-48.51)* |
| Senegal | 110018(94031,128625) | 20819.62(17794.4,24340.88) |  | 266868(228597,311180) | 20668.8(17704.72,24100.72) |  | 142.57(140.95,144.25) | -1.89(-3.43,-0.35)* |
| Serbia | 249097(212849,294696) | 11977.2(10234.3,14169.69) |  | 406568(349496,480488) | 14493.26(12458.78,17128.36) |  | 63.22(53.2,73.13) | 62.12(58.57,65.67)* |
| Seychelles | 1433(1230,1679) | 14938.09(12827.81,17503.62) |  | 2967(2529,3515) | 14304.81(12193,16947.46) |  | 107.08(100.14,113.61) | -13.87(-15.14,-12.6)* |
| Sierra Leone | 71869(61728,83674) | 21682.75(18623.13,25244.42) |  | 125576(107328,146409) | 20787.86(17767.03,24236.62) |  | 74.73(72.04,77.77) | -13.38(-15.77,-10.99)* |
| Singapore | 47410(40416,55433) | 13076.16(11147.16,15289) |  | 213151(184233,246342) | 14033.93(12129.95,16219.23) |  | 349.59(342.9,357.38) | 23.44(15.74,31.14)* |
| Slovakia | 139279(119520,165652) | 13425.1(11520.52,15967.13) |  | 230031(197983,272565) | 14023.9(12070.1,16617.04) |  | 65.16(63.7,66.75) | 14.34(11.87,16.81)* |
| Slovenia | 57945(50091,68623) | 13453.59(11629.97,15932.73) |  | 112549(97691,131550) | 15371.58(13342.31,17966.69) |  | 94.23(88.98,99.63) | 43.42(40.44,46.41)* |
| Solomon Islands | 1938(1639,2302) | 8571(7249.02,10182.92) |  | 4784(4080,5623) | 8818.56(7521.83,10366.7) |  | 146.86(142.12,152.1) | 9.25(8.67,9.83)* |
| Somalia | 68571(58126,81436) | 19043.87(16143.04,22616.8) |  | 176938(148096,210953) | 18762.07(15703.72,22369.01) |  | 158.03(153.98,162.45) | -4.51(-6.09,-2.93)* |
| South Africa | 636903(541048,739744) | 18868.55(16028.81,21915.27) |  | 1446633(1224933,1687179) | 18338.39(15527.99,21387.69) |  | 127.14(125.23,129.06) | -9.13(-11.5,-6.76)* |
| South Sudan | 90579(76585,106183) | 21841.95(18467.56,25604.57) |  | 124097(105384,145995) | 19928.07(16923.04,23444.6) |  | 37(32.81,41.56) | -29.47(-31.9,-27.04)* |
| Spain | 1281033(1109295,1518517) | 13399.09(11602.77,15883.07) |  | 2408618(2110065,2807777) | 15564.49(13635.25,18143.86) |  | 88.02(81.33,96.1) | 48.34(43.73,52.94)* |
| Sri Lanka | 273560(246018,302566) | 15370.48(13822.96,17000.24) |  | 742969(665457,820355) | 15426.12(13816.75,17032.88) |  | 171.59(169.28,173.77) | 1.13(0.29,1.97)* |
| Sudan | 118650(100801,141098) | 7978.93(6778.61,9488.51) |  | 244114(209703,288806) | 7839.66(6734.55,9274.93) |  | 105.74(101.74,110.02) | -5.61(-7.17,-4.05)* |
| Suriname | 8078(6934,9335) | 18561.85(15934.67,21452.29) |  | 20943(18009,24247) | 18749.09(16122.16,21706.96) |  | 159.28(156.82,161.99) | 3.16(1.79,4.52)* |
| Sweden | 495053(423452,574435) | 20852.48(17836.51,24196.18) |  | 720977(619451,827265) | 21416.39(18400.62,24573.66) |  | 45.64(43.97,47.24) | 8.62(7.26,9.97)* |
| Switzerland | 334396(289227,391452) | 19796.51(17122.46,23174.29) |  | 600728(523972,699242) | 20496.82(17877.9,23858.12) |  | 79.65(77.15,82.07) | 11.3(10.52,12.09)* |
| Syrian Arab Republic | 68573(59195,80281) | 7777.63(6713.93,9105.57) |  | 175658(150115,208319) | 7434.64(6353.53,8816.98) |  | 156.16(151.74,160.36) | -14.43(-16.32,-12.53)* |
| Taiwan (Province of China) | 162873(140044,192030) | 5872.2(5049.12,6923.42) |  | 487044(423534,568956) | 6468.1(5624.68,7555.92) |  | 199.03(187.83,211.01) | 31.12(29.59,32.65)* |
| Tajikistan | 59391(51345,69882) | 12681.77(10963.78,14922) |  | 115632(98497,137228) | 11257.65(9589.37,13360.13) |  | 94.7(86.85,102.35) | -38.38(-44.75,-32)* |
| Thailand | 838332(711289,997441) | 14126.93(11986.09,16808.12) |  | 2868416(2474569,3364602) | 14756.76(12730.58,17309.42) |  | 242.16(234.06,251.12) | 14.08(13.71,14.44)* |
| Timor-Leste | 6044(5080,7230) | 13906.17(11689.4,16636.12) |  | 20299(17200,24166) | 14292(12110.35,17014.7) |  | 235.86(220.37,253.6) | 8.48(3.83,13.14)* |
| Togo | 39410(33653,46151) | 20202.65(17251.33,23657.86) |  | 121456(103355,143275) | 19442.7(16545,22935.4) |  | 208.18(203.52,212.65) | -11.84(-13.32,-10.36)* |
| Tokelau | 22(19,26) | 9344.9(8022.76,11092.33) |  | 25(21,29) | 9808.2(8458.31,11592.41) |  | 12.1(9.56,15.04) | 15.85(11.39,20.32)* |
| Tonga | 858(736,1007) | 9043.33(7754.37,10611.19) |  | 1290(1121,1515) | 9606.06(8346.45,11278.4) |  | 50.34(46.94,54.62) | 19.72(18.36,21.08)* |
| Trinidad and Tobago | 26658(22661,30971) | 19415.74(16505.08,22556.87) |  | 66897(57208,77828) | 19204.69(16423.09,22342.52) |  | 150.95(147.31,155.28) | -3.57(-5.76,-1.37)* |
| Tunisia | 66491(56582,79236) | 7636.28(6498.28,9100) |  | 190712(164410,224937) | 8160.09(7034.68,9624.5) |  | 186.83(181.63,193.64) | 21.8(19.55,24.06)* |
| Turkey | 463634(399955,545052) | 7760.19(6694.34,9122.94) |  | 1362927(1176769,1604923) | 8249.54(7122.76,9714.29) |  | 193.97(188.59,199.77) | 19.97(17.47,22.47)* |
| Turkmenistan | 39424(33914,46580) | 12138.98(10442.34,14342.48) |  | 84099(72241,99228) | 11883.39(10207.84,14021.21) |  | 113.32(109.36,117.3) | -7.18(-11.58,-2.78)* |
| Tuvalu | 101(85,120) | 8664.16(7329.02,10300.72) |  | 164(141,194) | 9017.93(7715.51,10666.33) |  | 63.25(59.85,67.68) | 13.07(11.52,14.62)* |
| Uganda | 217648(184632,253225) | 20809.35(17652.7,24210.83) |  | 478986(406498,559721) | 20197.66(17141.03,23602.05) |  | 120.07(117.6,123.13) | -9.85(-13.11,-6.59)* |
| Ukraine | 1679072(1439859,2011970) | 13468.14(11549.38,16138.38) |  | 1950808(1678386,2318574) | 14367.35(12361.01,17075.88) |  | 16.18(14.43,18.22) | 20.38(15.87,24.89)* |
| United Arab Emirates | 4046(3453,4782) | 7208.09(6151.41,8520.14) |  | 40831(33241,50828) | 5686.09(4629.09,7078.28) |  | 909.26(811.15,999.61) | -77.56(-82.12,-73)* |
| United Kingdom | 2948554(2532661,3432614) | 19851.52(17051.46,23110.51) |  | 4313120(3732647,4984010) | 20509.18(17749,23699.31) |  | 46.28(44.18,48.39) | 10.54(7.6,13.48)* |
| United Republic of Tanzania | 374282(316581,438597) | 20745.36(17547.18,24310.15) |  | 865853(739888,1008400) | 21004.74(17948.95,24462.78) |  | 131.34(129.03,133.99) | 3.97(1.41,6.53)* |
| United States of America | 3595051(3382490,3841264) | 6852.65(6447.48,7321.96) |  | 6738241(6389525,7137692) | 6721.59(6373.73,7120.05) |  | 87.43(84.12,91.02) | -6.4(-8.12,-4.68)* |
| United States Virgin Islands | 2520(2134,2946) | 18043.73(15280.03,21094.28) |  | 6609(5644,7732) | 20530.98(17534.31,24019.81) |  | 162.23(151.23,172.95) | 42.03(38.34,45.73)* |
| Uruguay | 99091(85253,115236) | 14685.21(12634.45,17077.83) |  | 147759(127534,169198) | 16339.78(14103.21,18710.53) |  | 49.11(45.38,52.84) | 34.65(33.38,35.92)* |
| Uzbekistan | 254884(221133,299443) | 13032.94(11307.18,15311.39) |  | 537273(459323,636003) | 11557.61(9880.78,13681.45) |  | 110.79(102.8,119.28) | -39.33(-43.72,-34.93)* |
| Vanuatu | 866(734,1024) | 8690.63(7361.48,10272.35) |  | 2506(2129,2958) | 8737.9(7422.98,10315.07) |  | 189.27(186.32,192.16) | 2.04(0.98,3.1)* |
| Venezuela (Bolivarian Republic of) | 188626(162160,223547) | 12046.16(10355.95,14276.31) |  | 638314(550338,754609) | 12199.75(10518.32,14422.44) |  | 238.4(235.87,241.07) | 4.48(2.14,6.82)* |
| Viet Nam | 999156(856021,1183541) | 14330.79(12277.82,16975.4) |  | 2465224(2094171,2934135) | 14112.32(11988.21,16796.63) |  | 146.73(143.13,150.3) | -5.44(-7.04,-3.84)* |
| Yemen | 58815(49880,69865) | 7333.35(6219.32,8711.09) |  | 171502(146882,203109) | 7625.93(6531.2,9031.37) |  | 191.6(187.45,195.95) | 12.71(11.34,14.09)* |
| Zambia | 94625(80355,110700) | 20556.02(17456.18,24048.16) |  | 217334(184099,254481) | 20120.58(17043.69,23559.58) |  | 129.68(127.91,131.43) | -6.51(-8.33,-4.68)* |
| Zimbabwe | 116160(98990,137339) | 17524.91(14934.49,20720.1) |  | 186561(159042,220585) | 16848.71(14363.38,19921.47) |  | 60.61(58.2,63.5) | -12.56(-14.98,-10.14)* |

Abbeviation: UI, uncertainty interval; AAPC, average annual percent change; CI, confidence interval.

Note: * indicates statistically significant.
