# Supplementary material for: Long-term spatio-temporal trends in burden of fungal skin diseases in middle-aged and elderly people from 1990 to 2021
Source: PLoS Negl Trop Dis. 2026 Apr 1;20(4):e0014157. doi: 10.1371/journal.pntd.0014157 (PMC13065042; doi:10.1371/journal.pntd.0014157)
Supplement: S8 Table — (DOCX) [file pntd.0014157.s008.docx]

**S8 Table. Number of DALYs and DALYs rate of fungal skin diseases in middle-aged and elderly people across 204 countries and territories in 1990 and 2021, and temporal trends from 1990 to 2021.**

| Characteristic | 1990 | |  | 2021 | |  | 1990-2021 | |
| --- | --- | --- | --- | --- | --- | --- | --- | --- |
|  | DALYs | DALYs rate per 100000 |  | DALYs | DALYs rate per 100000 |  | Cases change | AAPC |
|  | No. (95% UI) | No. (95% UI) |  | No. (95% UI) | No. (95% UI) |  | % (95% UI) | (95% CI) |
| Afghanistan | 487(198,1041) | 40.05(16.31,85.69) |  | 517(214,1115) | 42.09(17.39,90.72) |  | 6.28(1.64,11.24) | 16.42(10.96,21.89)* |
| Albania | 232(96,475) | 66.97(27.75,136.96) |  | 569(235,1175) | 72.42(29.87,149.43) |  | 145.05(138.86,151.68) | 25.69(23.32,28.06)* |
| Algeria | 849(348,1805) | 40.55(16.61,86.15) |  | 2530(1030,5444) | 41.66(16.97,89.63) |  | 197.89(190.12,206.52) | 8.92(7.46,10.39)* |
| American Samoa | 2(1,4) | 47.11(18.92,99.41) |  | 4(2,9) | 47.74(19.14,100.88) |  | 134.08(128.52,139.94) | 4.61(3.11,6.11)* |
| Andorra | 9(4,20) | 94.13(38.15,200.58) |  | 27(11,56) | 100.17(40.89,211.58) |  | 185.37(177.91,193.9) | 19.94(12.22,27.66)* |
| Angola | 424(173,879) | 68.09(27.78,141.14) |  | 1318(537,2721) | 68.19(27.77,140.72) |  | 210.86(204.74,217.04) | 0.83(-1.05,2.71) |
| Antigua and Barbuda | 10(4,21) | 115.08(47.14,245.06) |  | 19(8,39) | 98.29(40.23,208.64) |  | 86.51(78.48,95.86) | -50.95(-52.47,-49.43)* |
| Argentina | 4165(1703,8698) | 74.85(30.61,156.3) |  | 7544(3080,15860) | 80.83(33,169.93) |  | 81.12(76.84,85.03) | 25.12(23.16,27.09)* |
| Armenia | 316(129,647) | 64.93(26.47,132.91) |  | 566(233,1166) | 71.9(29.67,148.22) |  | 78.96(74.42,84.11) | 32.31(27.46,37.16)* |
| Australia | 3949(1633,8057) | 120.32(49.77,245.51) |  | 9637(3990,19550) | 130.1(53.86,263.91) |  | 144.08(141.42,146.86) | 24.77(22.89,26.65)* |
| Austria | 2001(821,4191) | 102.82(42.18,215.39) |  | 3178(1288,6646) | 107.55(43.57,224.9) |  | 58.85(55.33,62.22) | 14.96(11.53,18.39)* |
| Azerbaijan | 575(237,1172) | 65.88(27.12,134.33) |  | 1177(475,2423) | 61.42(24.8,126.46) |  | 104.76(97.9,112.3) | -22.84(-26.93,-18.75)* |
| Bahamas | 25(10,54) | 102.52(41.76,218.14) |  | 69(28,148) | 96.5(39.03,205.27) |  | 175.84(168.73,183.38) | -19.67(-21.13,-18.21)* |
| Bahrain | 10(4,22) | 36.46(14.89,77.22) |  | 56(22,118) | 34.31(13.63,72.76) |  | 444.7(424.46,467.17) | -18.63(-23.7,-13.56)* |
| Bangladesh | 4071(1648,8708) | 53.74(21.76,114.96) |  | 12581(5095,27003) | 53.84(21.8,115.57) |  | 209.06(202.55,215.03) | 0.85(-1.18,2.87) |
| Barbados | 55(23,118) | 117.62(48.53,250.82) |  | 97(39,206) | 106.22(42.84,225.65) |  | 75.51(68.87,83.05) | -33.38(-35.35,-31.4)* |
| Belarus | 1609(662,3317) | 69.52(28.61,143.3) |  | 2086(862,4326) | 72.51(29.95,150.37) |  | 29.6(27.41,31.79) | 13.73(9.39,18.06)* |
| Belgium | 2636(1076,5499) | 100.53(41.04,209.75) |  | 4099(1674,8521) | 108.41(44.28,225.39) |  | 55.5(51.98,59.36) | 24.15(21.99,26.32)* |
| Belize | 16(7,35) | 107.11(43.36,226.54) |  | 49(20,104) | 98.42(39.8,208.25) |  | 199.01(191.16,207.53) | -27.23(-27.97,-26.5)* |
| Benin | 370(150,768) | 115.55(46.93,239.96) |  | 912(373,1911) | 110.3(45.07,231.06) |  | 146.76(141.41,152.07) | -14.37(-18.58,-10.16)* |
| Bermuda | 11(4,23) | 103.1(41.8,218.2) |  | 26(11,55) | 111.69(45.17,237.39) |  | 138.51(132.54,145.04) | 25.61(23.46,27.76)* |
| Bhutan | 20(8,42) | 50.08(20,106.2) |  | 55(22,119) | 55.43(22.27,119.76) |  | 177.73(166.41,190.5) | 32.83(31.48,34.19)* |
| Bolivia (Plurinational State of) | 699(290,1526) | 133.27(55.22,291.12) |  | 2074(854,4511) | 134.19(55.27,291.86) |  | 196.77(193.28,200.12) | 2.22(0.36,4.08)* |
| Bosnia and Herzegovina | 462(189,940) | 62.99(25.77,128.15) |  | 807(335,1674) | 73.67(30.63,152.86) |  | 74.62(66.53,83.24) | 51.38(47.29,55.47)* |
| Botswana | 85(34,179) | 91.27(36.42,192.9) |  | 221(87,460) | 91.05(36.05,189.67) |  | 160.15(155.93,165.18) | 0.49(-1.35,2.33) |
| Brazil | 14445(5909,30557) | 97.74(39.98,206.77) |  | 44689(18106,94595) | 103.19(41.81,218.42) |  | 209.38(204.03,214.79) | 17.47(16.58,18.36)* |
| Brunei Darussalam | 11(4,23) | 70.69(28.58,148.56) |  | 38(16,81) | 63.99(25.91,135.16) |  | 246.16(234.71,258.52) | -32.76(-39,-26.51)* |
| Bulgaria | 1505(625,3127) | 66.48(27.6,138.15) |  | 1867(775,3905) | 78.53(32.62,164.31) |  | 24.07(18.2,30.18) | 54.37(51.68,57.06)* |
| Burkina Faso | 786(324,1670) | 108.63(44.74,230.91) |  | 1669(685,3506) | 111.01(45.54,233.19) |  | 112.42(109.14,115.77) | 6.76(3.16,10.36)* |
| Burundi | 425(171,881) | 112.84(45.32,234) |  | 865(354,1805) | 108.05(44.2,225.5) |  | 103.67(96.49,111.01) | -14.03(-15.56,-12.5)* |
| Cabo Verde | 49(20,103) | 125.28(50.38,261.34) |  | 87(35,182) | 113.88(45.99,236.93) |  | 76.53(68.76,85.38) | -30.4(-34.44,-26.35)* |
| Cambodia | 553(221,1178) | 74.61(29.89,159.05) |  | 1618(641,3441) | 74.43(29.48,158.29) |  | 192.66(187.82,197.75) | -0.68(-1.39,0.02) |
| Cameroon | 728(291,1541) | 98.27(39.31,208.08) |  | 2006(801,4231) | 98.53(39.33,207.84) |  | 175.62(172.08,179.35) | 1.32(-0.68,3.32) |
| Canada | 1863(753,3919) | 34.13(13.79,71.79) |  | 4396(1800,9156) | 35.82(14.66,74.62) |  | 135.95(129.62,141.69) | 15.2(11.83,18.57)* |
| Central African Republic | 126(51,259) | 66.53(26.95,137.1) |  | 235(95,484) | 65.62(26.59,135.36) |  | 86.84(83.46,90.59) | -4.47(-6.71,-2.23)* |
| Chad | 533(216,1113) | 114.95(46.57,240.09) |  | 1006(414,2117) | 108.35(44.63,227.96) |  | 88.87(83.75,94.29) | -19.38(-24.06,-14.7)* |
| Chile | 1235(506,2585) | 73.44(30.06,153.74) |  | 3466(1411,7261) | 77.4(31.5,162.16) |  | 180.61(175.06,186.29) | 17.01(15.22,18.79)* |
| China | 53346(21398,110798) | 37.17(14.91,77.2) |  | 146642(59739,304183) | 38.7(15.76,80.27) |  | 174.89(168.8,182.08) | 13.13(11.51,14.76)* |
| Colombia | 1817(732,3900) | 63.1(25.44,135.45) |  | 6501(2638,14012) | 67.98(27.58,146.52) |  | 257.9(248.91,267.29) | 24.07(22.96,25.18)* |
| Comoros | 35(14,73) | 108.72(44.03,225.31) |  | 90(36,186) | 111.96(44.95,230.39) |  | 156.5(151.04,162.16) | 9.94(6.15,13.74)* |
| Congo | 125(51,259) | 70.47(28.75,145.28) |  | 301(123,622) | 69.31(28.32,143.25) |  | 140.1(135.29,145.56) | -5.4(-7.73,-3.06)* |
| Cook Islands | 1(0,2) | 48.56(19.59,102.45) |  | 2(1,5) | 50.36(20.41,105.56) |  | 129.02(122.33,136.81) | 11.34(8.33,14.35)* |
| Costa Rica | 190(76,405) | 66.75(26.82,142.56) |  | 642(259,1379) | 67.04(27.01,143.99) |  | 238.09(232.11,244.25) | 1.13(-0.6,2.87) |
| Croatia | 734(302,1499) | 65.92(27.17,134.66) |  | 1178(492,2449) | 78.97(32.97,164.19) |  | 60.47(50.93,69.02) | 59.2(54.42,63.99)* |
| Cuba | 1825(743,3893) | 107.68(43.82,229.73) |  | 3671(1492,7762) | 107.42(43.65,227.14) |  | 101.14(97.93,105.2) | -0.73(-2.57,1.12) |
| Cyprus | 130(53,278) | 93.11(37.94,198.9) |  | 350(143,740) | 100.17(41.01,211.69) |  | 169.15(162.27,176.66) | 23.61(22.72,24.5)* |
| Czechia | 1701(700,3512) | 71.85(29.58,148.3) |  | 2772(1162,5796) | 79.35(33.25,165.9) |  | 62.93(58.48,67.84) | 32(28.94,35.07)* |
| CÔTE D'IVOIRE | 648(265,1379) | 101.11(41.4,215.16) |  | 1888(774,3985) | 105.29(43.14,222.24) |  | 191.35(185.48,196.84) | 13.22(11.76,14.68)* |
| Democratic People's Republic of Korea | 894(352,1905) | 33.57(13.24,71.57) |  | 1995(792,4230) | 35.41(14.05,75.05) |  | 123.32(115.14,132.52) | 17.21(15.64,18.77)* |
| Democratic Republic of the Congo | 1782(725,3667) | 67.49(27.47,138.87) |  | 4148(1674,8546) | 68.67(27.72,141.49) |  | 132.73(128.06,137.96) | 5.57(2.73,8.42)* |
| Denmark | 1383(569,2908) | 105.97(43.58,222.92) |  | 2083(852,4386) | 108.19(44.27,227.82) |  | 50.65(48.48,52.54) | 7.21(2.58,11.85)* |
| Djibouti | 22(9,47) | 103.5(42.19,217.5) |  | 109(45,229) | 105.35(43.08,220.8) |  | 391.98(384.47,399.26) | 5.82(4.24,7.4)* |
| Dominica | 11(4,23) | 109.14(44.78,232.92) |  | 15(6,32) | 100.25(40.83,213.04) |  | 41.11(36.1,46.62) | -27.33(-28.5,-26.15)* |
| Dominican Republic | 612(249,1307) | 100.12(40.66,213.74) |  | 1745(706,3710) | 104.41(42.24,222.01) |  | 185.03(179.97,190.7) | 13.47(11,15.94)* |
| Ecuador | 1193(488,2577) | 138.79(56.81,299.75) |  | 3895(1603,8362) | 140.75(57.93,302.14) |  | 226.4(222.34,230.78) | 5.83(4.8,6.87)* |
| Egypt | 1736(689,3599) | 38.83(15.42,80.49) |  | 4144(1647,8567) | 37.45(14.88,77.42) |  | 138.65(132.41,144.1) | -11.52(-13.8,-9.25)* |
| El Salvador | 328(132,701) | 67(27,143.29) |  | 729(299,1575) | 71.3(29.25,154.05) |  | 122.56(117.41,128.16) | 19.99(18.28,21.71)* |
| Equatorial Guinea | 22(9,46) | 69.46(28.22,142.97) |  | 56(23,117) | 71.18(29.15,147.84) |  | 153.92(149.36,159) | 8.13(5.76,10.5)* |
| Eritrea | 170(69,357) | 96.43(39.1,202.28) |  | 447(181,919) | 101.43(41.04,208.6) |  | 162.25(154.58,170.66) | 16.68(13.72,19.65)* |
| Estonia | 255(105,525) | 71.11(29.42,146.49) |  | 363(151,756) | 82.89(34.54,172.87) |  | 42.27(37.44,47.12) | 49.81(45.31,54.31)* |
| Eswatini | 42(17,87) | 93.12(36.99,193.02) |  | 81(32,167) | 89.3(35.31,184.58) |  | 92.27(88.82,95.72) | -13.12(-14.08,-12.17)* |
| Ethiopia | 4442(1836,9643) | 138.26(57.14,300.14) |  | 10209(4188,21481) | 149.13(61.18,313.79) |  | 129.8(121.21,137.91) | 22.86(19.21,26.51)* |
| Fiji | 27(11,56) | 47.08(18.89,98.56) |  | 63(25,134) | 46.39(18.55,98.18) |  | 139.08(133.97,144.71) | -4.51(-6.72,-2.3)* |
| Finland | 1186(486,2493) | 99.92(40.96,209.96) |  | 2225(913,4651) | 110.56(45.34,231.07) |  | 87.56(82.37,92.94) | 32.23(28.09,36.38)* |
| France | 14252(5792,29810) | 102.41(41.62,214.21) |  | 24684(10075,51355) | 111.64(45.57,232.27) |  | 73.19(69.46,77.14) | 27.89(26.4,29.38)* |
| Gabon | 74(30,153) | 75.09(30.69,155.78) |  | 126(51,262) | 70.4(28.41,146.83) |  | 71.19(66.48,75.77) | -20.12(-24.54,-15.71)* |
| Gambia | 61(25,129) | 109.86(45.14,232.28) |  | 174(71,362) | 112.26(45.86,234.06) |  | 185.34(180.65,190.06) | 6.93(5.09,8.77)* |
| Georgia | 765(314,1573) | 68.83(28.24,141.65) |  | 799(331,1647) | 76.09(31.53,156.87) |  | 4.49(1.63,7.57) | 32.83(27.83,37.83)* |
| Germany | 21407(8777,45031) | 102.02(41.83,214.61) |  | 34422(14037,72196) | 109.23(44.54,229.09) |  | 60.8(56.91,64.74) | 21.94(17.41,26.48)* |
| Ghana | 576(238,1231) | 56.41(23.27,120.59) |  | 1554(633,3331) | 56.99(23.23,122.15) |  | 169.8(165.11,174.95) | 3.51(1.04,5.97)* |
| Greece | 2596(1056,5493) | 97.09(39.48,205.46) |  | 4256(1735,8895) | 115.81(47.2,242.02) |  | 63.97(56.61,70.74) | 57.48(55.15,59.81)* |
| Greenland | 2(1,3) | 27.28(10.79,58.92) |  | 4(1,8) | 27.61(10.93,59.3) |  | 149.36(142.3,156.26) | 3.25(-2.8,9.3) |
| Grenada | 14(6,29) | 115.1(47.08,244.27) |  | 19(8,41) | 96.69(39.25,206.1) |  | 39.93(33.41,46.78) | -56.12(-58.97,-53.27)* |
| Guam | 6(2,13) | 46.55(18.69,99.51) |  | 20(8,41) | 52.46(21.28,109.8) |  | 229.37(214.05,248.27) | 38.69(36.14,41.24)* |
| Guatemala | 343(137,729) | 59.63(23.84,126.83) |  | 1187(479,2549) | 64.85(26.17,139.21) |  | 246.2(235.89,256.68) | 27.19(25.51,28.87)* |
| Guinea | 630(257,1325) | 113.82(46.39,239.49) |  | 1027(417,2157) | 112.39(45.65,236.09) |  | 63.02(60.75,65.19) | -3.76(-5.88,-1.63)* |
| Guinea-Bissau | 68(28,145) | 107.03(44.24,226.99) |  | 119(49,254) | 103.73(42.52,220.89) |  | 74.41(70.92,77.78) | -10.22(-12.85,-7.59)* |
| Guyana | 60(24,127) | 97.92(39.7,207.07) |  | 106(43,224) | 93.73(37.79,199.04) |  | 75.86(72.78,79.3) | -13.62(-15.94,-11.29)* |
| Haiti | 498(199,1056) | 92.42(36.95,195.81) |  | 1089(438,2314) | 91.85(36.97,195.23) |  | 118.38(115.74,121.46) | -1.93(-4.16,0.31) |
| Honduras | 211(84,449) | 63.18(25.35,134.62) |  | 656(265,1408) | 62.39(25.2,133.91) |  | 211.56(206.31,217.07) | -4.41(-9.12,0.31) |
| Hungary | 1811(743,3737) | 70.32(28.83,145.08) |  | 2540(1052,5296) | 79.87(33.07,166.52) |  | 40.23(36.07,44.59) | 41.47(36.13,46.82)* |
| Iceland | 49(20,102) | 102.71(41.84,214.8) |  | 102(41,212) | 104.67(42.57,217.79) |  | 107.99(105.13,110.8) | 6.06(4.15,7.96)* |
| India | 37199(14887,76946) | 48.52(19.42,100.36) |  | 102988(41498,215963) | 51.23(20.64,107.42) |  | 176.86(170.5,184.28) | 17.49(15.11,19.86)* |
| Indonesia | 12300(4849,26841) | 76.13(30.01,166.14) |  | 31663(12502,69230) | 75.61(29.85,165.31) |  | 157.43(155.55,159) | -2.2(-3.27,-1.13)* |
| Iran (Islamic Republic of) | 1817(723,3817) | 39.42(15.69,82.8) |  | 5746(2308,11915) | 44.23(17.77,91.72) |  | 216.21(202.06,231.16) | 37.71(35.79,39.63)* |
| Iraq | 565(230,1221) | 44.76(18.21,96.75) |  | 1522(625,3282) | 39.17(16.09,84.49) |  | 169.35(159.08,179) | -42.85(-45.89,-39.81)* |
| Ireland | 684(281,1441) | 100.44(41.27,211.74) |  | 1356(550,2850) | 103.29(41.93,217.15) |  | 98.26(94.88,102.05) | 8.62(6.88,10.35)* |
| Israel | 1214(496,2486) | 149.76(61.2,306.75) |  | 3109(1275,6369) | 155.56(63.8,318.7) |  | 156.13(152.87,159.48) | 12.77(9.15,16.39)* |
| Italy | 15264(6168,32224) | 100.29(40.53,211.73) |  | 25901(10542,54354) | 114.02(46.41,239.27) |  | 69.69(63.47,75.8) | 40.82(35.74,45.9)* |
| Jamaica | 330(134,702) | 112.1(45.66,238.4) |  | 572(231,1211) | 108.23(43.72,229.03) |  | 73.37(69.15,78.68) | -11.22(-14.19,-8.25)* |
| Japan | 22774(9257,46714) | 76.91(31.26,157.75) |  | 54424(22499,110929) | 104.26(43.1,212.5) |  | 138.97(122.97,154.18) | 98.92(92.78,105.06)* |
| Jordan | 85(35,181) | 39.61(16.14,84.1) |  | 491(201,1052) | 39.05(16,83.68) |  | 476.57(463.78,491.92) | -4.27(-9.02,0.48) |
| Kazakhstan | 1411(584,2892) | 67.49(27.95,138.28) |  | 2037(835,4153) | 64.18(26.31,130.87) |  | 44.29(40.99,47.15) | -16.57(-21.59,-11.54)* |
| Kenya | 1199(488,2537) | 89.94(36.59,190.35) |  | 3234(1296,6793) | 86.45(34.64,181.58) |  | 169.77(154.42,189.45) | -12.46(-13.46,-11.46)* |
| Kiribati | 3(1,6) | 46.99(18.91,98.71) |  | 6(2,12) | 45.81(17.98,96.47) |  | 105.79(100.24,111.08) | -8.3(-9.83,-6.77)* |
| Kuwait | 36(15,77) | 39.42(16.09,84.13) |  | 184(75,389) | 39.57(16.06,83.5) |  | 411.77(399.4,427.23) | 5.09(-17.75,27.99) |
| Kyrgyzstan | 351(144,723) | 68.08(28.06,140.51) |  | 538(219,1097) | 63.61(25.88,129.74) |  | 53.45(49.05,58.18) | -21.86(-27.79,-15.92)* |
| Lao People's Democratic Republic | 258(103,548) | 75.23(29.94,159.87) |  | 589(235,1253) | 76.19(30.45,162.06) |  | 128.33(124.17,132.8) | 4.3(3.23,5.37)* |
| Latvia | 446(185,925) | 71.41(29.64,147.98) |  | 534(223,1114) | 81.2(33.91,169.32) |  | 19.68(15.41,23.48) | 40.94(38.85,43.03)* |
| Lebanon | 155(64,332) | 40.93(16.83,87.52) |  | 483(199,1026) | 49.29(20.3,104.69) |  | 211.3(193,231.02) | 61.02(57.44,64.6)* |
| Lesotho | 137(55,282) | 97.67(39.45,201.61) |  | 164(65,341) | 90.97(36.21,188.72) |  | 20.26(17.54,23.2) | -22.59(-25.91,-19.27)* |
| Liberia | 212(87,443) | 112.56(46.33,234.99) |  | 351(144,741) | 106.21(43.5,224.51) |  | 65.32(60.57,70.68) | -19.47(-23.54,-15.4)* |
| Libya | 135(55,291) | 43.43(17.69,93.59) |  | 344(141,735) | 41.13(16.87,87.89) |  | 154.7(149.61,160.37) | -17.42(-21.06,-13.79)* |
| Lithuania | 567(235,1176) | 71.57(29.65,148.39) |  | 778(324,1622) | 80.41(33.5,167.56) |  | 37.21(32.69,41.81) | 38.07(35.56,40.58)* |
| Luxembourg | 92(38,194) | 98.68(40.35,208.11) |  | 183(74,382) | 102.75(41.88,215.11) |  | 98.14(93.77,102.49) | 13(11.93,14.06)* |
| Madagascar | 926(374,1921) | 109.58(44.23,227.3) |  | 1888(770,3940) | 102.26(41.71,213.37) |  | 103.98(98.18,109.84) | -22.25(-23.97,-20.54)* |
| Malawi | 678(277,1405) | 107.72(43.98,223.35) |  | 1276(518,2648) | 109.12(44.3,226.5) |  | 88.29(85.51,91.35) | 4.71(2.31,7.11)* |
| Malaysia | 1155(466,2423) | 78.26(31.54,164.15) |  | 3750(1501,7877) | 76.48(30.62,160.67) |  | 224.65(219.24,230.11) | -7.59(-8.67,-6.52)* |
| Maldives | 11(5,25) | 74.85(29.81,162.46) |  | 42(17,90) | 78.05(31.29,165.46) |  | 269.99(259.7,283.77) | 13.59(11.83,15.35)* |
| Mali | 935(380,2011) | 139.9(56.76,300.77) |  | 2051(831,4382) | 141.69(57.41,302.79) |  | 119.22(116.21,121.99) | 4.04(2.68,5.41)* |
| Malta | 70(28,147) | 96.37(38.99,202.57) |  | 172(71,364) | 109.18(44.73,230.69) |  | 146.79(138.68,155.02) | 40.4(37.44,43.37)* |
| Marshall Islands | 1(0,3) | 48.18(19.42,101.95) |  | 3(1,6) | 45.11(17.9,94.65) |  | 115.08(106.34,124.25) | -21.28(-22.99,-19.57)* |
| Mauritania | 189(77,398) | 113.64(46.28,239.99) |  | 405(166,849) | 112.77(46.28,236.55) |  | 114.45(110.66,117.98) | -1.64(-4.28,1.01) |
| Mauritius | 92(36,193) | 75.23(29.97,158.87) |  | 259(104,543) | 76.59(30.85,160.54) |  | 183.18(177.73,189.26) | 5.68(4.6,6.76)* |
| Mexico | 4147(1700,9113) | 59.64(24.45,131.08) |  | 15238(6157,31399) | 70.71(28.57,145.7) |  | 267.47(243.44,292.41) | 54.37(49.49,59.25)* |
| Micronesia (Federated States of) | 4(2,8) | 49.05(19.86,102.58) |  | 6(2,13) | 45.69(18.03,96.85) |  | 53.98(46.3,60.75) | -23.07(-25.71,-20.43)* |
| Monaco | 12(5,26) | 111.84(45.85,235.18) |  | 17(7,37) | 111.28(45.54,234.9) |  | 40.47(38.59,42.37) | -1.65(-3.65,0.35) |
| Mongolia | 117(49,242) | 67.67(28.1,139.94) |  | 241(98,493) | 60.92(24.78,124.75) |  | 105.12(96.86,112.87) | -34.11(-37.59,-30.64)* |
| Montenegro | 75(31,154) | 69.23(28.31,141.66) |  | 123(51,253) | 70.38(29.15,145.2) |  | 62.72(59.17,66.56) | 6.01(3.67,8.34)* |
| Morocco | 1024(420,2207) | 43.68(17.91,94.14) |  | 2497(1024,5310) | 41.56(17.04,88.39) |  | 143.8(138.29,149.43) | -16.25(-18.06,-14.44)* |
| Mozambique | 1044(425,2157) | 106.87(43.54,220.77) |  | 1880(768,3899) | 105.08(42.94,217.92) |  | 80.01(77.61,82.48) | -5.4(-7.64,-3.16)* |
| Myanmar | 2935(1164,6268) | 74.98(29.75,160.12) |  | 6404(2538,13560) | 75.85(30.06,160.62) |  | 118.16(114,122.58) | 3.92(2.81,5.03)* |
| Namibia | 100(40,209) | 91.82(36.7,192.89) |  | 210(84,438) | 92.9(37.17,193.94) |  | 110.67(106.05,115.28) | 3.78(2.61,4.95)* |
| Nauru | 0(0,1) | 46.56(18.55,98.75) |  | 0(0,1) | 46.37(18.74,97.14) |  | 27.35(24.21,31.06) | -1.49(-2.99,0) |
| Nepal | 772(310,1649) | 50.13(20.14,107.11) |  | 2068(834,4488) | 52.25(21.07,113.39) |  | 168(159.81,176.86) | 13.42(12.2,14.63)* |
| Netherlands | 3379(1383,7109) | 102.05(41.77,214.7) |  | 6153(2506,12907) | 105.16(42.83,220.6) |  | 82.11(79.58,84.54) | 10.02(7.65,12.39)* |
| New Zealand | 794(326,1619) | 120.68(49.48,246.08) |  | 1786(731,3645) | 125.19(51.22,255.55) |  | 124.9(121.98,128.3) | 11.73(8.49,14.97)* |
| Nicaragua | 158(63,339) | 63.47(25.46,136.48) |  | 521(210,1116) | 64(25.74,137.1) |  | 230.35(225.06,236.07) | 2.84(0.48,5.21)* |
| Niger | 584(236,1262) | 132.8(53.72,287.06) |  | 1845(746,3940) | 132.52(53.58,283) |  | 216.1(211.48,220.76) | 0.8(-2.65,4.24) |
| Nigeria | 10765(4456,22433) | 147.77(61.17,307.94) |  | 21485(8910,44939) | 143.22(59.39,299.56) |  | 99.58(96.19,102.84) | -9.65(-14.45,-4.84)* |
| Niue | 0(0,0) | 54.58(22.17,115.09) |  | 0(0,0) | 49.83(20.11,104.27) |  | -1.91(-6.32,2.09) | -29.57(-33.32,-25.81)* |
| North Macedonia | 219(90,449) | 66.58(27.45,136.34) |  | 399(166,828) | 67.26(27.96,139.72) |  | 82.07(76.46,87.63) | 4.05(-1.31,9.41) |
| Northern Mariana Islands | 1(0,2) | 46.2(18.16,98.23) |  | 4(2,10) | 46.07(18.41,97.86) |  | 297.04(285.87,307.81) | -0.87(-4,2.27) |
| Norway | 1181(481,2490) | 109.08(44.45,229.95) |  | 1754(712,3677) | 108.17(43.92,226.74) |  | 48.5(46.49,50.65) | -2.99(-4.43,-1.54)* |
| Oman | 42(17,89) | 41.31(16.97,88.51) |  | 118(48,249) | 37.8(15.26,79.81) |  | 184.35(174.06,193.63) | -28.92(-37.11,-20.72)* |
| Pakistan | 5031(2041,10746) | 55.16(22.38,117.83) |  | 10442(4173,22123) | 52.67(21.05,111.59) |  | 107.58(102.45,112.74) | -14.8(-16.01,-13.6)* |
| Palau | 1(0,2) | 48.49(19.47,102.42) |  | 2(1,4) | 45.75(18.39,96.88) |  | 146.9(137.38,156.77) | -18.5(-21.68,-15.32)* |
| Palestine | 62(25,132) | 42.49(17.35,91.07) |  | 168(69,359) | 38.97(15.94,83.54) |  | 171.88(163.66,180.47) | -26.86(-29.84,-23.88)* |
| Panama | 162(65,347) | 66.66(26.72,143.19) |  | 513(208,1100) | 68.99(27.94,147.91) |  | 217.6(211.08,223.84) | 11.09(9.58,12.6)* |
| Papua New Guinea | 135(53,286) | 45.5(17.99,96.82) |  | 380(150,798) | 46.3(18.24,97.11) |  | 182.47(175.93,189.83) | 6.12(2.94,9.3)* |
| Paraguay | 371(151,789) | 102.36(41.6,217.41) |  | 997(406,2119) | 100.74(40.96,214.01) |  | 168.59(165.05,172) | -5.06(-6.41,-3.71)* |
| Peru | 2729(1100,5808) | 138.41(55.8,294.58) |  | 7993(3256,16892) | 142.91(58.2,301.99) |  | 192.89(188.5,196.96) | 10.42(9.91,10.93)* |
| Philippines | 3690(1504,7855) | 77.3(31.5,164.53) |  | 10802(4375,22945) | 77.46(31.38,164.54) |  | 192.7(189.5,195.48) | 0.73(0.12,1.35)* |
| Poland | 5460(2224,11356) | 70.85(28.86,147.37) |  | 9547(3886,20006) | 78.8(32.08,165.13) |  | 74.86(71.39,78.78) | 34.89(32.54,37.24)* |
| Portugal | 2340(956,4913) | 95.98(39.21,201.52) |  | 4349(1772,9167) | 111.79(45.53,235.62) |  | 85.87(78.5,93.19) | 49.57(47.82,51.32)* |
| Puerto Rico | 651(266,1383) | 107.29(43.79,227.97) |  | 1394(573,2949) | 119.43(49.06,252.62) |  | 114.17(107.54,121.72) | 34.71(32.45,36.97)* |
| Qatar | 6(2,12) | 34.76(13.97,73.51) |  | 50(20,108) | 32.51(12.93,70.26) |  | 777.15(742.54,808.65) | -19.19(-22.5,-15.87)* |
| Republic of Korea | 3283(1336,6876) | 65.96(26.84,138.17) |  | 12713(5179,26580) | 76.02(30.97,158.94) |  | 287.28(273.2,302.68) | 45.79(41.92,49.66)* |
| Republic of Moldova | 506(211,1037) | 65.54(27.32,134.26) |  | 770(318,1582) | 72.94(30.11,149.87) |  | 52.21(47.49,57.71) | 34.71(29.56,39.88)* |
| Romania | 3358(1380,6942) | 67.1(27.58,138.74) |  | 4857(2026,10148) | 80.88(33.73,168.98) |  | 44.66(38.19,50.79) | 60.64(57.21,64.07)* |
| Russian Federation | 21816(8914,44988) | 69.12(28.24,142.54) |  | 31441(12843,65447) | 73.97(30.21,153.97) |  | 44.12(41.68,47.07) | 21.26(14.99,27.53)* |
| Rwanda | 502(204,1043) | 107.32(43.67,222.91) |  | 1128(458,2337) | 106.7(43.31,220.99) |  | 124.67(121.67,127.95) | -1.63(-6.27,3.01) |
| Saint Kitts and Nevis | 7(3,15) | 110.44(45.82,237.63) |  | 12(5,25) | 90.93(36.24,192.68) |  | 65.83(53.02,80.39) | -62.94(-67.54,-58.34)* |
| Saint Lucia | 15(6,32) | 104.25(42.75,221.93) |  | 43(17,91) | 102.5(41.41,217.35) |  | 182.8(176.34,190.23) | -5.42(-6.43,-4.4)* |
| Saint Vincent and the Grenadines | 13(5,27) | 105.65(43.12,224.95) |  | 26(10,55) | 101.4(41.16,215.14) |  | 103.11(98.82,108.12) | -12.55(-14.41,-10.68)* |
| Samoa | 7(3,15) | 48.41(19.5,101.58) |  | 12(5,25) | 48.65(19.42,101.71) |  | 71.71(68.13,75.46) | 1.47(-0.09,3.02) |
| San Marino | 6(2,13) | 103.09(42.07,216.44) |  | 13(5,28) | 112.67(46.15,234.21) |  | 120.09(113.28,127.19) | 28.71(27.41,30.02)* |
| Sao Tome and Principe | 13(5,27) | 114.33(46.52,239.29) |  | 20(8,42) | 108.87(44.6,229.74) |  | 55.49(52.56,58.86) | -16.49(-21.25,-11.72)* |
| Saudi Arabia | 369(150,790) | 41.43(16.89,88.67) |  | 1066(431,2241) | 35.42(14.31,74.42) |  | 188.87(173.74,204.67) | -50.4(-53.82,-46.98)* |
| Senegal | 588(242,1236) | 111.34(45.77,233.85) |  | 1426(583,3007) | 110.46(45.13,232.91) |  | 142.41(139.15,145.73) | -1.7(-3.16,-0.23)* |
| Serbia | 1333(545,2718) | 64.08(26.21,130.69) |  | 2146(888,4462) | 76.52(31.65,159.05) |  | 61.07(50.69,71.43) | 57.76(53.52,62.01)* |
| Seychelles | 8(3,16) | 80.26(32.19,166) |  | 16(6,34) | 76.5(30.65,161.61) |  | 106.1(98.76,113.27) | -15.67(-18.14,-13.19)* |
| Sierra Leone | 383(155,799) | 115.53(46.63,240.92) |  | 672(274,1402) | 111.18(45.39,232.06) |  | 75.39(72.09,78.77) | -11.94(-14.01,-9.86)* |
| Singapore | 256(104,539) | 70.55(28.75,148.66) |  | 1150(469,2430) | 75.74(30.89,160) |  | 349.73(340.21,359.5) | 23.62(16.03,31.22)* |
| Slovakia | 734(302,1507) | 70.79(29.1,145.28) |  | 1214(499,2509) | 74.01(30.41,152.97) |  | 65.29(62.88,68.06) | 14.73(11.71,17.75)* |
| Slovenia | 304(126,624) | 70.65(29.19,144.88) |  | 589(245,1220) | 80.51(33.51,166.58) |  | 93.71(87.77,99.35) | 42.3(39.72,44.88)* |
| Solomon Islands | 10(4,22) | 46.39(18.35,99.35) |  | 26(10,54) | 47.36(18.97,99.18) |  | 144.94(138.84,151.61) | 6.68(5.78,7.57)* |
| Somalia | 368(150,766) | 102.33(41.7,212.61) |  | 950(387,1989) | 100.72(41.05,210.93) |  | 157.8(152.28,163.4) | -4.59(-6.42,-2.76)* |
| South Africa | 3383(1372,7076) | 100.23(40.63,209.62) |  | 7634(3109,16013) | 96.77(39.41,203) |  | 125.64(123.49,127.71) | -11.32(-13.5,-9.15)* |
| South Sudan | 479(194,996) | 115.56(46.81,240.08) |  | 658(267,1361) | 105.69(42.85,218.56) |  | 37.34(32.63,42.16) | -28.64(-30.05,-27.23)* |
| Spain | 6826(2834,14568) | 71.4(29.64,152.38) |  | 12725(5296,26690) | 82.23(34.22,172.47) |  | 86.41(79.32,93.84) | 45.6(40.94,50.26)* |
| Sri Lanka | 1476(603,3107) | 82.92(33.87,174.59) |  | 3964(1631,8384) | 82.31(33.87,174.07) |  | 168.63(163.74,173.51) | -2.2(-3.39,-1)* |
| Sudan | 632(258,1356) | 42.49(17.35,91.21) |  | 1297(528,2755) | 41.64(16.96,88.49) |  | 105.24(99.71,111.55) | -6.46(-8.08,-4.83)* |
| Suriname | 43(17,92) | 99.54(40.17,210.95) |  | 111(45,235) | 99.54(40.4,209.97) |  | 156.67(152.69,160.62) | 0.24(-1.09,1.58) |
| Sweden | 2632(1074,5580) | 110.87(45.24,235.05) |  | 3817(1555,8044) | 113.39(46.18,238.95) |  | 45.02(43.02,46.91) | 7.25(5.74,8.76)* |
| Switzerland | 1767(722,3703) | 104.59(42.77,219.23) |  | 3168(1293,6629) | 108.1(44.11,226.19) |  | 79.33(76.18,82.56) | 10.78(9.69,11.87)* |
| Syrian Arab Republic | 366(149,782) | 41.48(16.91,88.67) |  | 935(384,1995) | 39.57(16.25,84.43) |  | 155.62(149.06,162.11) | -15.1(-17.59,-12.61)* |
| Taiwan (Province of China) | 883(356,1880) | 31.85(12.84,67.78) |  | 2608(1056,5514) | 34.64(14.02,73.23) |  | 195.24(183.81,209.93) | 26.87(25.1,28.64)* |
| Tajikistan | 319(131,659) | 68.12(28.01,140.67) |  | 624(253,1287) | 60.78(24.67,125.33) |  | 95.7(87.41,104.09) | -36.47(-42.04,-30.9)* |
| Thailand | 4513(1807,9562) | 76.04(30.44,161.13) |  | 15367(6188,32212) | 79.06(31.83,165.72) |  | 240.54(231.15,250.13) | 12.65(11.76,13.53)* |
| Timor-Leste | 33(13,71) | 75(29.9,163) |  | 108(44,227) | 76.36(30.8,159.92) |  | 232.73(215.27,251) | 6.22(0.29,12.15)* |
| Togo | 212(87,447) | 108.42(44.84,229.18) |  | 655(269,1400) | 104.82(43.13,224.07) |  | 209.59(204.3,215.85) | -10.96(-13.31,-8.61)* |
| Tokelau | 0(0,0) | 50.03(20.2,106.21) |  | 0(0,0) | 51.86(21.02,109.22) |  | 10.7(7.41,14.17) | 11.7(7.57,15.82)* |
| Tonga | 5(2,10) | 48.7(19.66,103.02) |  | 7(3,14) | 51.15(20.74,107.4) |  | 48.63(44.28,53.44) | 15.9(14.67,17.13)* |
| Trinidad and Tobago | 143(58,305) | 103.81(42.44,222.13) |  | 355(145,756) | 101.96(41.6,216.99) |  | 149.19(144.71,154.71) | -5.77(-8.28,-3.26)* |
| Tunisia | 356(145,760) | 40.88(16.64,87.33) |  | 1005(410,2177) | 42.99(17.55,93.14) |  | 182.3(174.84,190.69) | 17.12(13.71,20.53)* |
| Turkey | 2481(1009,5286) | 41.53(16.89,88.48) |  | 7219(2934,15681) | 43.69(17.76,94.91) |  | 190.98(183.66,199.89) | 16.97(13.95,19.99)* |
| Turkmenistan | 212(88,435) | 65.29(26.95,133.84) |  | 452(184,922) | 63.91(25.99,130.31) |  | 113.27(107.78,118.92) | -6.82(-10.57,-3.07)* |
| Tuvalu | 1(0,1) | 46.81(18.77,99.25) |  | 1(0,2) | 48.29(19.47,101.68) |  | 61.82(57.63,66.49) | 9.79(7.81,11.77)* |
| Uganda | 1162(470,2405) | 111.06(44.93,229.94) |  | 2565(1037,5291) | 108.15(43.72,223.1) |  | 120.81(117.1,124.75) | -8.8(-11.97,-5.63)* |
| Ukraine | 8863(3607,18360) | 71.09(28.94,147.27) |  | 10284(4208,21273) | 75.74(30.99,156.67) |  | 16.04(13.62,18.41) | 20.04(16.71,23.37)* |
| United Arab Emirates | 22(9,46) | 38.41(15.63,81.18) |  | 221(88,475) | 30.72(12.26,66.17) |  | 923.19(825.56,1020.44) | -72.92(-77.45,-68.39)* |
| United Kingdom | 15712(6389,33159) | 105.79(43.02,223.25) |  | 22828(9273,47884) | 108.55(44.1,227.69) |  | 45.28(43.21,47.31) | 8.31(6.41,10.2)* |
| United Republic of Tanzania | 2004(824,4292) | 111.08(45.7,237.88) |  | 4643(1888,9973) | 112.64(45.79,241.93) |  | 131.68(127.74,135.61) | 4.42(2.08,6.75)* |
| United States of America | 18906(7826,37887) | 36.04(14.92,72.22) |  | 34877(14639,69161) | 34.79(14.6,68.99) |  | 84.47(81.05,88.32) | -11.59(-13.27,-9.91)* |
| United States Virgin Islands | 14(6,29) | 97.22(39.51,206.26) |  | 35(14,75) | 108.91(44.85,232.09) |  | 158.16(146.88,169.29) | 36.68(33.36,40)* |
| Uruguay | 529(217,1101) | 78.36(32.17,163.11) |  | 780(320,1632) | 86.31(35.39,180.5) |  | 47.6(43.71,51.8) | 31.4(29.71,33.08)* |
| Uzbekistan | 1366(559,2807) | 69.86(28.6,143.54) |  | 2887(1175,5953) | 62.1(25.27,128.06) |  | 111.28(102.94,120.13) | -38.47(-41.82,-35.12)* |
| Vanuatu | 5(2,10) | 47.01(18.85,99.86) |  | 13(5,28) | 47.04(18.92,99.37) |  | 187.93(181.92,194.45) | 0.42(-1.16,2) |
| Venezuela (Bolivarian Republic of) | 1006(404,2149) | 64.24(25.8,137.21) |  | 3391(1369,7271) | 64.8(26.16,138.98) |  | 237.09(231.99,242.66) | 2.89(0.6,5.18)* |
| Viet Nam | 5393(2147,11371) | 77.35(30.79,163.1) |  | 13340(5358,28290) | 76.36(30.67,161.95) |  | 147.37(142.6,152.12) | -4.32(-5.74,-2.9)* |
| Yemen | 314(128,673) | 39.11(15.92,83.89) |  | 911(375,1928) | 40.5(16.66,85.75) |  | 190.33(182.98,197.21) | 11.44(9.85,13.03)* |
| Zambia | 507(205,1051) | 110.18(44.51,228.37) |  | 1161(472,2407) | 107.51(43.72,222.87) |  | 128.95(125.85,132.3) | -7.56(-8.93,-6.19)* |
| Zimbabwe | 625(251,1309) | 94.33(37.91,197.56) |  | 997(395,2087) | 90.06(35.65,188.5) |  | 59.49(56.37,62.97) | -14.75(-17.3,-12.21)* |

Abbreviation: DALYs, disability-adjusted life-years; UI, uncertainty interval; AAPC, average annual percent change; CI, confidence interval.

Note: * indicates statistically significant.
